# Salivary microbiome profiling reveals a dysbiotic schizophrenia-associated microbiota

**DOI:** 10.1038/s41537-021-00180-1

**Published:** 2021-10-28

**Authors:** Ying Qing, Lihua Xu, Gaoping Cui, Liya Sun, Xiaowen Hu, Xuhan Yang, Jie Jiang, Juan Zhang, Tianhong Zhang, Tao Wang, Lin He, Jijun Wang, Chunling Wan

**Affiliations:** 1 Bio-X Institutes, Key Laboratory for the Genetics of Developmental and Neuropsychiatric Disorders, Ministry of Education, Shanghai Jiao Tong University, Shanghai, China; 2 Shanghai Mental Health Centre, Shanghai Jiao Tong University School of Medicine, Shanghai Key Laboratory of Psychotic Disorders, Shanghai, China; 3 Department of Bioinformatics and Biostatistics, Shanghai Jiao Tong University, Shanghai, China; 4 SJTU-Yale Joint Center for Biostatistics and Data Science, Shanghai Jiao Tong University, Shanghai, China

**Keywords:** Microbiology, Schizophrenia, Biomarkers

## Abstract

Schizophrenia is a debilitating mental disorder and often has a prodromal period, referred to as clinical high risk (CHR) for psychosis, prior to the first episode. The etiology and pathogenesis of schizophrenia remain unclear. Despite the human gut microbiome being associated with schizophrenia, the role of the oral microbiome, which is a vital player in the mouth–body connection, is not well understood. To address this, we performed 16S rRNA gene sequencing to investigate the salivary microbiome in 85 patients with drug-naïve first-episode schizophrenia (FES), 43 individuals at CHR, and 80 healthy controls (HCs). The salivary microbiome of FES patients was characterized by higher α-diversity and lower β-diversity heterogeneity than those of CHR subjects and HCs. Proteobacteria, the predominant phylum, was depleted, while Firmicutes and the Firmicutes/Proteobacteria ratio was enriched, in a stepwise manner from HC to CHR to FES. H_2_S-producing bacteria exhibited disease-stage-specific enrichment and could be potential diagnostic biomarkers for FES and CHR. Certain salivary microbiota exhibited disease-specific correlation patterns with symptomatic severities, peripheral pro-inflammatory cytokines, thioredoxin, and S100B in FES. Furthermore, the metabolic functions from inferred metagenomes of the salivary microbiome were disrupted in FES, especially amino acid metabolism, carbohydrate metabolism, and xenobiotic degradation. This study has established a link between salivary microbiome alterations and disease initiation and provided the hypothesis of how the oral microbiota could influence schizophrenia.

## Introduction

Schizophrenia is a severe, complex, debilitating mental illness; is one of the top 15 principal causes of global disability in 2016; and is a heavy burden on society, the economy and public health^1^. As the putative prodrome of schizophrenia, the clinical high-risk (CHR) state is characterized by attenuated psychotic symptoms, recent functional deterioration and/or genetic risks, with a transition rate of 29% over 2 years^2,3^. Accordingly, attention should be paid to CHR cohorts when studying schizophrenia, especially first-episode schizophrenia (FES). Despite persistent investigation into this disease for more than one hundred years, the etiology and pathogenesis of schizophrenia remain unknown. Extensive research has focused mainly on genetic factors to determine the pathogenesis of the disease. However, the identified associations possibly account for only a small portion of the genetic susceptibility, suggesting that the etiology of schizophrenia is multifactorial. Recently, a body of evidence has implicated the human microbiome in the pathophysiology and etiology of schizophrenia^4–19^. Accordingly, it is necessary to identify the role of microorganisms residing on or within the human body in schizophrenia initiation and progression.

More than 700 microbes inhabit the human oral cavity, which is not only the entry point into the body for exogenous microorganisms but also the beginning of the respiratory and digestive tracts^20,21^. As a vital player in the mouth–body connection, oral microbiota performs an essential protective function against invasion and colonization by external microbes that can affect the host’s health. Dysbiosis of the oral microbiome can lead to oral diseases and whole-body systemic diseases, such as caries, periodontitis, inflammatory bowel disease, and Alzheimer’s disease^22^. It has been hypothesized that oral microorganisms can reach the brain by various direct and indirect means, suggesting possible involvement of the oral microbiome in the pathophysiology of brain disorders^23,24^. Recently, several studies have reported associations between the salivary microbiome and anxiety and depression symptoms^25^ and autism spectrum disorder (ASD)^26–28^, highlighting the links between the oral microbiome and mental disorders. Nevertheless, research on the oral microbiome’s associations with mental disorders is still in its infancy. Thus, the characteristics and function of oral microbes implicated in psychotic disorders, such as schizophrenia, require further investigation.

To date, studies on the human microbiome in schizophrenia have only reported alterations in the gut^4,6–8,10,12–16,19^ as well as oropharyngeal^5,17,18^ microbiota and have noted that transplantation of fecal bacteria from patients with schizophrenia can lead to schizophrenia-like behaviors in mice through the “microbiota–gut–brain” axis^10,11^. Notably, the oropharynx tends to have higher numbers of gastrointestinal organisms than do the dental plaque or saliva. Therefore, the role of oral microbiota in schizophrenia remains largely unknown. Given that saliva acts as an important reservoir of microorganisms from all the distinct ecological niches of the mouth and plays an important role in promoting the relationship between the resident oral microbes and host health^29,30^, it is crucial to identify the role of salivary microbes in schizophrenia.

Hence, this study aimed to comprehensively investigate the salivary microbiome in the context of schizophrenia, to characterize the microbial profiles at different clinical stages of the disease, and to gain understanding of the function of salivary microbes in the initiation of schizophrenia. The insights gained should shed new light on how the human microbiome influences schizophrenia, providing new possibilities for therapeutic interventions, and thereby contributing to reducing the burden of this serious disease.

## Results

### The saliva microbial community exhibits an overall structural change following the onset of schizophrenia

**Fig. 1:**
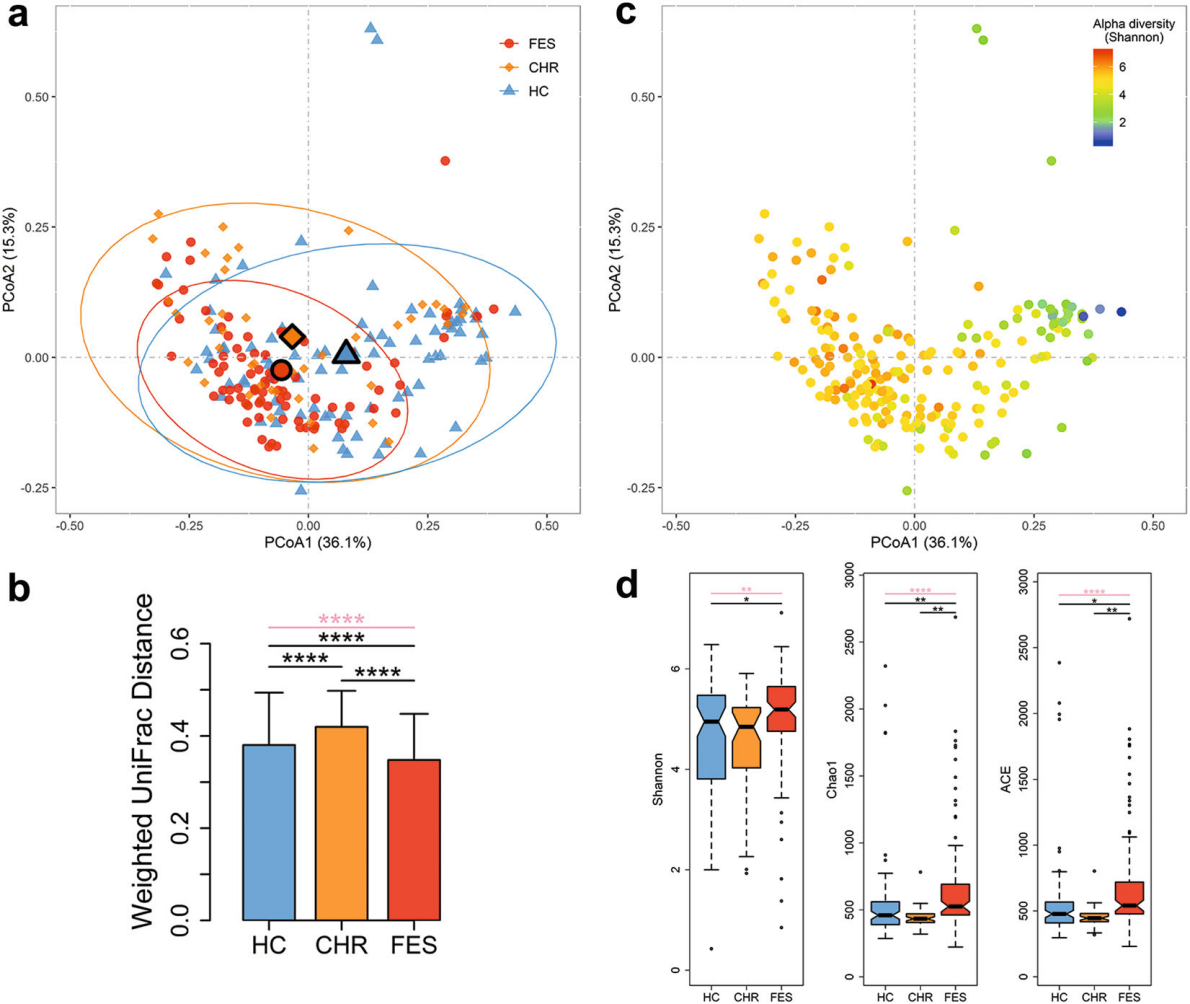
The overall salivary microbiome composition differs according to different clinical stages of schizophrenia (FES, CHR and HC) in 208 subjects. The principal coordinate analysis was conducted based on the weighted UniFrac distances, with 95% confidence ellipses drawn and centroids representing the coordinate mean of the first and second axes. Each sample is colored either by the disease phenotype (**a**) or the Shannon diversity index (**c**). **b** Comparison of within-group distances among the three groups. The bar plots show median values for each group and error bars show interquartile range. **d** Comparison of three α-diversity indices among the three groups. Center lines of box plots show median values, box hinges indicate first and third quartiles, and whisker represent the furthest data points within 1.5 interquartile ranges of the hinges. The comparisons among the three groups were performed by the Kruskal–Wallis test and the comparisons between the two groups were conducted by the quantile regression, adjusting for age, gender, and education level. *p < 0.05; **p < 0.01; ***p < 0.001; ****p < 0.0001. Pink lines represent comparisons among the three groups.

To investigate whether the overall microbiome composition differed according to the different clinical stages of schizophrenia, we carried out β-diversity analysis, i.e., PCoA analysis based on weighted UniFrac phylogenetic distances, and found a significant difference in composition among the FES, CHR, and HC groups (*p* = 0.0035; Fig. 1a). After controlling for age, gender, and education level, the result of β-diversity analysis remained statistically significant (*p* = 0.008), indicating that the relationship between β-diversity and group status was not greatly influenced by these potentially confounding factors. As shown in Fig. 1a, the sample distributions of the FES and HC groups were more concentrated than that of the CHR group. To further support this finding, we compared the within-group distances among the three groups and observed that the FES group had the lowest β-diversity heterogeneity, while the CHR group had the highest β-diversity heterogeneity (Fig. 1b). We also found that the overall diversity in microbial composition tended to be differentiated by the microbial α-diversity, i.e., the Shannon index (Fig. 1c). To verify this finding, we next conducted within-sample α-diversity analysis to examine salivary bacterial community variations at different stages of schizophrenia. The microbial diversity index (Shannon) and richness indices (Chao1 and Ace) were all higher in FES patients than in either the CHR or HC group, excluding outliers and adjusting for age, gender, and education level (Fig. 1d). These findings suggest that the salivary microbial compositions of patients suffering from FES were characterized by high α-diversity and low β-diversity heterogeneity and that the CHR and HC groups were very similar.

### The salivary microbiota is altered in schizophrenia, especially the H_2_S-producing bacteria enriched

**Fig. 2:**
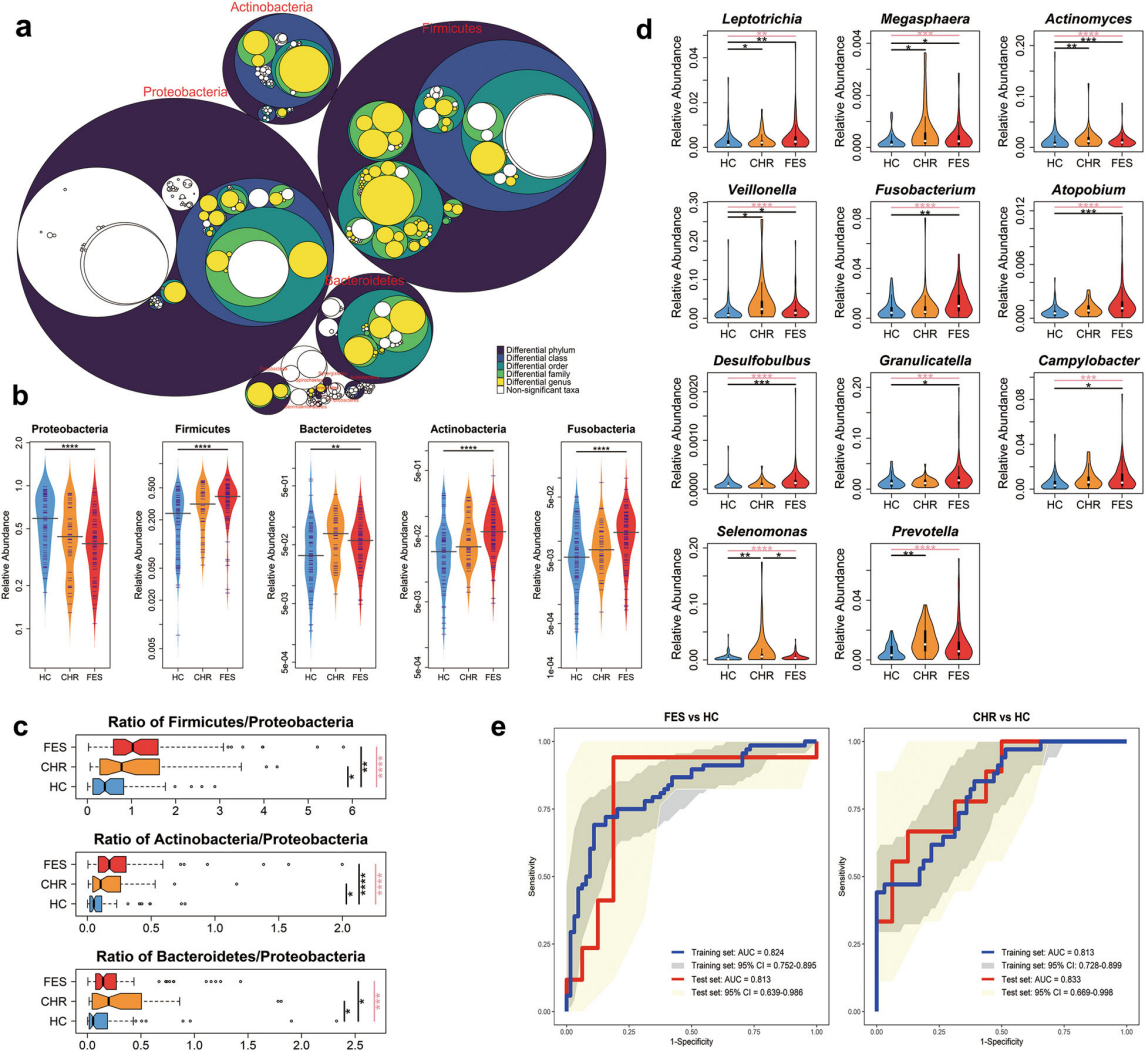
Differential abundances of salivary bacterial communities during initiation of schizophrenia. **a** Differentially abundant taxa between the FES, CHR, and HC groups are colored. The largest circles represent the phylum level, and the inner circles represent class, order, family, and genus. **b** The leading five abundant phyla differed in abundances among the three groups. Center lines of bean plots represent median values. **c** The ratios of Firmicutes/Proteobacteria, Actinobacteria/Proteobacteria, and Bacteroidetes/Proteobacteria were higher in FES and CHR patients than in HCs. Center lines of box plots show median values, box hinges indicate first and third quartiles, and whisker represent the furthest data points within 1.5 interquartile ranges of the hinges. **d** H_2_S-producing bacteria were enriched in either the FES or the CHR group than in HCs. **e** Receiver operating characteristic (ROC) curves for the logistic regression models. The area under the curve (AUC) values for distinguishing FES from HCs in the training and test sets were 0.824 (sensitivity: 0.691; specificity: 0.891) and 0.813 (sensitivity: 0.941; specificity: 0.813), respectively. AUCs for distinguishing CHR from HCs in the training and test sets were 0.813 (sensitivity: 0.853; specificity: 0.609) and 0.833 (sensitivity: 0.667; specificity: 0.875), respectively. The comparisons among the three groups were performed by the Kruskal–Wallis test and the *q* values were corrected with FDR; the comparisons between the two groups were conducted by the quantile regression, adjusting for age, gender and education level and correcting with FDR. *q < 0.05; **q < 0.01; ***q < 0.001; ****q < 0.0001. Pink lines represent comparisons among the three groups.

To further identify the salivary taxa responsible for the specific microbiome composition at different clinical stages of schizophrenia, we performed the Kruskal–Wallis test, which was verified by the Jonckheere–Terpstra test, to compare the relative abundances of taxa between the FES, CHR, and HC groups and found that ten phyla exhibited marked changes across the three groups (Fig. 2a and Supplementary Table 1). Of the leading five differentially abundant phyla, Proteobacteria and Firmicutes, which largely dominated the microbial communities in saliva, displayed inverse changes, where Proteobacteria was gradually depleted and Firmicutes was progressively enriched in a stepwise manner from HC to CHR to FES (Fig. 2b). Moreover, the change trends of Actinobacteria and Fusobacteria were parallel with that of Firmicutes in the three groups (Fig. 2b). In addition, the Firmicutes/Proteobacteria, Actinobacteria/Proteobacteria, and Bacteroidetes/Proteobacteria ratios differed significantly between the three groups, being higher in the FES and CHR groups than in HCs (Fig. 2c), excluding outliers and adjusting for confounders. Out of the 612 taxa included in the pairwise comparison analyses, 114 were altered in FES, while only 24 were altered in CHR, and only 10 altered taxa overlapped between the FES and CHR groups relative to HCs (Supplementary Figs. 1 and 2). These findings revealed that the salivary microbiota exhibited disease-stage-specific alterations and changed increasingly dramatically with the initiation of schizophrenia. Salivary microbial alterations might indicate development of the disease.

Of these differentially abundant taxa, H_2_S-producing bacteria stood out. The nine genera *Leptotrichia*, *Megasphaera*, *Actinomyces*, *Veillonella*, *Fusobacterium*, *Atopobium*, *Desulfobulbus*, *Granulicatella,* and *Campylobacter* were noticeably enriched in FES, while *Leptotrichia*, *Megasphaera*, *Actinomyces*, *Veillonella*, *Selenomonas*, and *Prevotella* were more abundant in CHR, after correction for confounders and multiple comparisons (Fig. 2d). Furthermore, the whole dataset was split into training and test sets with a split ratio of 4:1. And the differentially abundant H_2_S-producing bacteria between disease and HC groups in the training set were selected for model training. Seven H_2_S-producing bacteria, including *Leptotrichia*, *Actinomyces*, *Veillonella*, *Fusobacterium*, *Atopobium*, *Desulfobulbus,* and *Granulicatella*, were capable of discriminating FES patients from HCs with AUCs of 0.824 and 0.813 in training and test sets, respectively. Four H_2_S-producing bacteria, which were *Leptotrichia*, *Actinomyces*, *Selenomonas*, and *Prevotella*, were able to distinguish CHR subjects from HCs with AUCs of 0.813 and 0.833 in training and test sets, respectively (Fig. 2e). These findings indicated that enrichment of H_2_S-producing bacteria in saliva was, to some extent, correlated with increased risk of initiation of schizophrenia and thus represented a promising classifier for the auxiliary diagnosis of the disease. Detailed results of comparative taxonomic analyses are described in Supplementary Note 1 and Supplementary Fig. 3.

### The saliva microbiome is associated with the clinical characteristics of schizophrenia and the prodromal psychosis stage

**Fig. 3:**
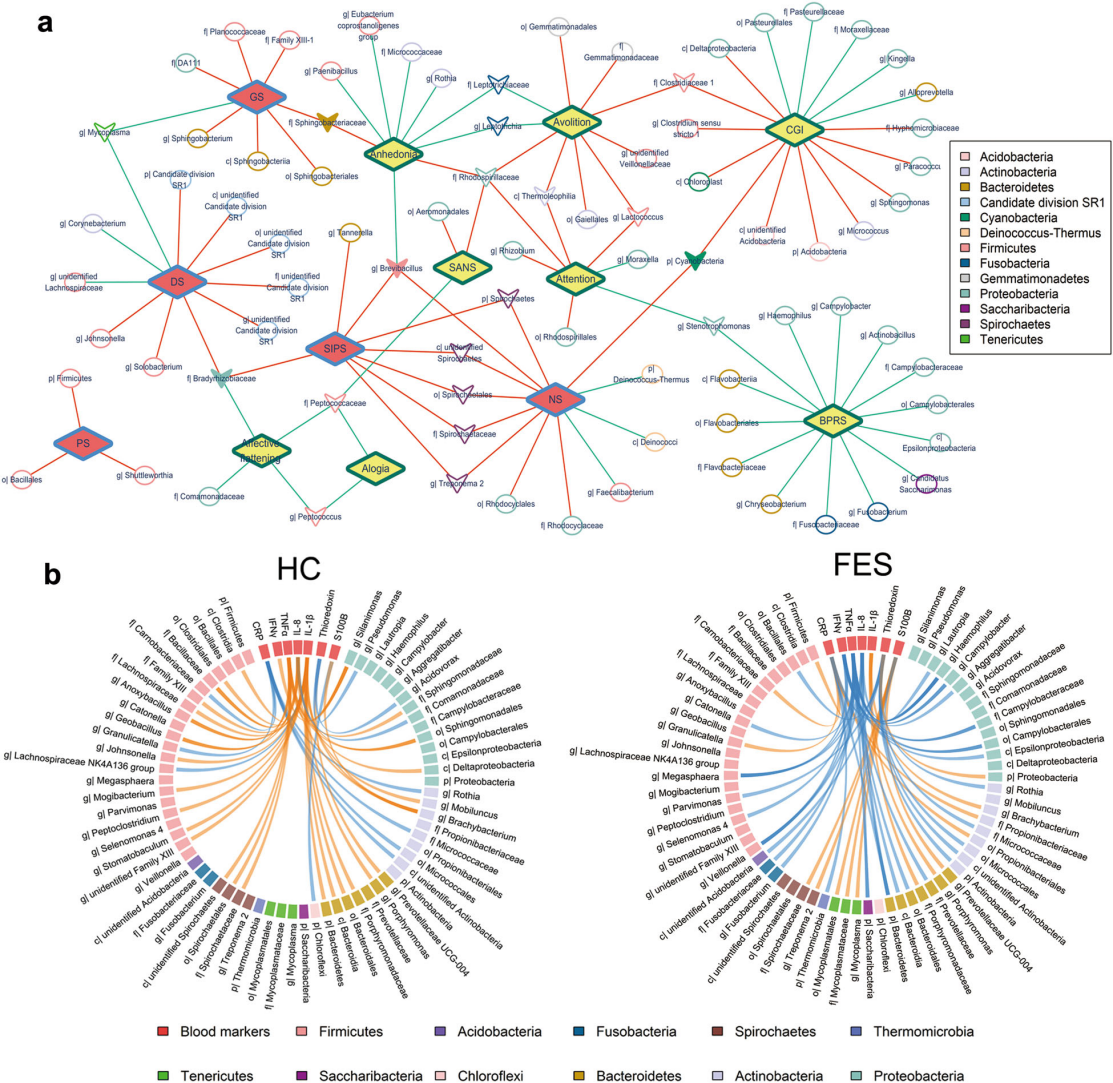
Salivary taxa are correlated with symptomatic severities and blood markers relevant to inflammation in schizophrenia. **a** The correlation network of salivary microbiota with symptoms of two disease statuses. Red lines denote positive correlations, while green lines denote negative correlations. Yellow diamonds represent symptoms of schizophrenia, while red diamonds represent CHR symptoms. Ellipses denote taxa relevant to a single symptom of either schizophrenia or CHR, and hollow inverted triangles indicate taxa associated with more than one symptom of either schizophrenia or CHR, while solid inverted triangles represent taxa related to symptoms of both schizophrenia and CHR. **b** The Circos plot showed distinct relationships of salivary taxa with blood markers (CRP, IFNγ, TNFα, IL-8, IL-1β, thioredoxin, and S100B) in the FES group relative to HCs. Orange curves denote positive correlations, while blue curves denote negative correlations. GS general symptom, DS disorganized symptom, NS negative symptoms, PS positive symptoms, SIPS structured interview of prodrome syndromes, BPRS brief psychiatric rating scale, CGI-S clinical global impressions severity scale, SANS scale for the assessment of negative symptoms, p phylum, c class, o order, f family, g genus.

To determine whether salivary taxa were related to the severity of schizophrenic and pre-psychotic symptoms, we carried out Spearman’s partial correlation analyses, adjusting for confounders (Supplementary Table 2). As shown in Fig. 3a and Supplementary Fig. 4a, several taxa displayed unique relationships with BPRS, CGI, or SANS in FES patients. For example, two H_2_S-producing bacteria, *Campylobacter* and *Fusobacterium*, were both negatively correlated with BPRS, suggesting that enrichment of these two H_2_S-producing bacteria was likely associated with attenuated psychosis. Likewise, Firmicutes, its class Bacillales and its genus *Shuttleworthia* showed exclusively positive relationships with the positive symptoms of CHR. These results implied that salivary taxa might have a distinct impact on different clinical manifestations of the different stages of schizophrenia.

To further evaluate whether the salivary microbiome was connected with the peripheral inflammatory response, we performed Spearman’s partial correlation analyses between the altered salivary taxa and seven important blood markers, adjusting for confounders (Supplementary Table 3). Somewhat surprisingly, the correlation patterns of salivary microbes with these markers in the FES group were almost completely distinct from those in HCs (Fig. 3b, Supplementary Note 2 and Supplementary Fig. 4b). In the FES group, Bacteroidetes, Thermomicrobia, and *Haemophilus* were negatively correlated with inflammatory marker C-reactive protein (CRP). Similarly, the pathogenic bacteria *Aggregatibacter*, *Campylobacter*, *Fusobacterium*, *Haemophilus*, *Veillonella* as well as 12 other taxa had significant inverse relationships with pro-inflammatory cytokines interferonγ (IFNγ), interleukin-8 (IL-8), and/or tumor necrosis factor-α (TNFα) in the FES group. Actinobacteria and its subtaxa exhibited markedly negative correlations with the redox indicator thioredoxin and certain salivary taxa, such as *Mycoplasma*, its family Mycoplasmataceae and its order Mycoplasmatales, also displayed specific relationships with S100B, an indicator for brain damage and blood–brain barrier disruption. In contrast, these patterns were not observed in HCs. Interestingly, IL-1β, unlike the other six blood markers, was significantly correlated with 14 salivary taxa in only HCs and not in the FES group. These disease-specific correlations between the salivary microbiome and peripheral blood markers provided clues for connections between the oral cavity, peripheral circulation, and the brain.

### The metabolic functions of the salivary microbiome were disturbed in schizophrenia

**Fig. 4:**
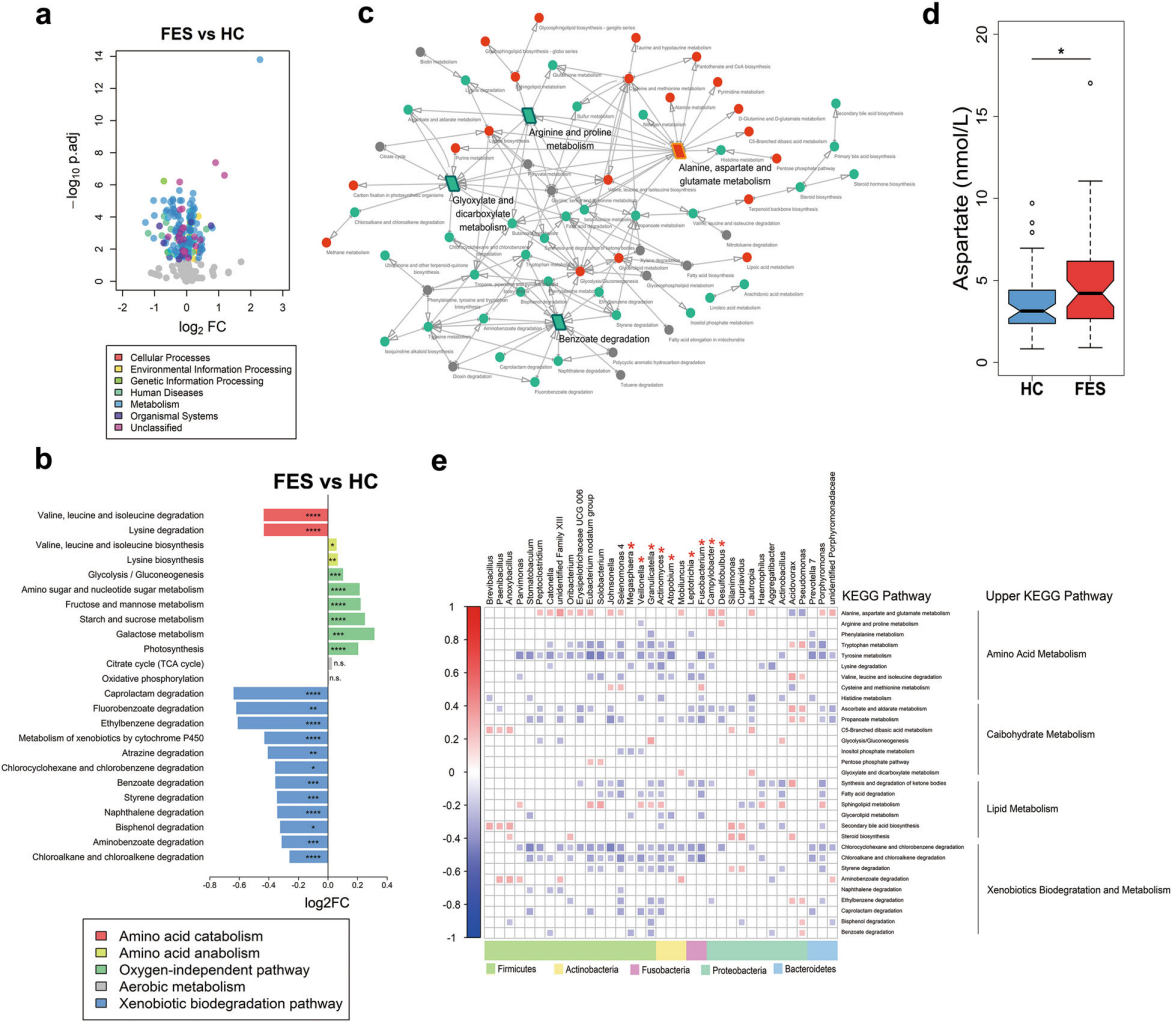
The functions of the salivary microbiota were dysregulated in the FES and CHR groups, especially those of metabolism related pathways. **a** A volcano plot shows the differentially abundant KEGG pathways in FES versus HCs. **b** Selected metabolic pathways associated with amino acid catabolism and anabolism, oxygen-independent pathway and xenobiotic biodegradation pathway were differentially abundant in the FES group relative to HCs. False discovery rate adjusted *q* values were calculated based on *p* values estimated by DESeq2, adjusting for age, gender, and education level. *q < 0.05; **q < 0.01; ***q < 0.001; ****q < 0.0001; n.s. indicates no significance. **c** Correlation network of KEGG pathways classified as the term metabolism based on their upstream/downstream relationships. Parallelograms indicate trigger pathways or main terminal pathways. Red dots/parallelograms represent pathways enriched in the FES group compared to HCs, while green represents depleted pathways, and gray denotes pathways with no significance. **d** Levels of plasma l-aspartate were increased in the FES group relative to HCs, controlling for confounders. Center lines of box plots show median values, box hinges indicate first and third quartiles, and whisker represent the furthest data points within 1.5 interquartile ranges of the hinges. *P* values were calculated by quantile regression, adjusting for age, gender, and education level. *p < 0.05. **e** The heatmap shows correlations of the selected genera of interest with certain KEGG pathways. Only statistically significant correlations (*p* < 0.05) are shown. Red asterisk indicates H_2_S-producing bacteria. KEGG Kyoto Encyclopedia of Gene and Genomes, FC fold change.

To explore the functional composition of the saliva-associated microbiota across different stages of schizophrenia, we conducted pairwise comparisons based on inferred metagenomes, adjusting for confounders. Interestingly, pathways corresponding to the term “metabolism” exhibited drastic changes in the FES group relative to HCs (Fig. 4a, Supplementary Table 4 and Supplementary Note 3). CHR resembled FES in the functional changes of salivary microbiota compared to HCs (Supplementary Figs. 5 and 6). In the FES group, pathways related to amino acid catabolism (valine, leucine, and isoleucine degradation and lysine degradation) were depleted, whereas anabolism of these amino acids was enriched (Fig. 4b), suggesting a tendency to accumulate branched-chain amino acids (BCAAs) and lysine. Moreover, oxygen-independent pathways were enriched in FES, whereas aerobic metabolism was not altered (Fig. 4b), implying a preference for a facultative anaerobic oral environment in schizophrenia. Furthermore, xenobiotic biodegradation pathways were significantly depleted in the FES group (Fig. 4b), indicating that some fundamental function was compromised in the disease. Based on the upstream/downstream relationships of the differential pathways, a core network of the Kyoto Encyclopedia of Gene and Genomes (KEGG) metabolic pathways was established in FES, where the enriched “alanine, aspartate, and glutamate metabolism” and depleted “arginine and proline metabolism” pathways played the central role as trigger pathways, and the reduced “benzoate degradation” and “glyoxylate and dicarboxylate metabolism” pathways acted as the main terminals of the cascade of changes in the pathways (Fig. 4c). Interestingly, the plasma levels of l-aspartate, an important amino acid involved in “alanine, aspartate, and glutamate metabolism”, were significantly higher in the FES group than in HCs, which might to some extent be due to the disturbances in microbial amino acid metabolic functions (Fig. 4d). In addition, bacterial genera altered in the FES group relative to HCs were mainly related to multiple pathways classified as amino acid metabolism, carbohydrate metabolism, lipid metabolism, and xenobiotics biodegradation and metabolism (Fig. 4e), suggesting that altered salivary microbial communities might influence the host’s disease status through changing these metabolic pathways.

## Discussion

A growing body of evidence indicates that the human microbiome plays a vital role in brain development^31^ and participates in the pathophysiology of schizophrenia^10,11^. The current study provides unprecedented insight into the identity, quantity, and functions of microbes in the saliva of patients with schizophrenia and provides a unique framework for understanding oral microbial dysbiosis in this disease.

We identified a noticeable difference in the β-diversity of the salivary microbiome between the FES, CHR, and HC groups and revealed a lower heterogeneity of β-diversity and a higher α-diversity of the microbial composition in FES patients than the other two groups. That is, although the saliva of each patient with schizophrenia was ecologically rich, members of the FES group shared similar microorganisms. This is a striking and intriguing finding for this disease, and further exploration is required to determine whether this feature is part of the etiology of schizophrenia or whether it is a consequence of disease status. Consistent with our result showing high microbial α-diversity in saliva, a study by Loohuis et al.^32^ reported increased microbial α-diversity in whole-blood samples of patients with schizophrenia. The blood microbiome is mainly attributed to the translocation from the oral cavity and the gut^33^. Despite this, studies on the gut microbiome of patients with schizophrenia have revealed inconsistent changes in α-diversity^9,10,12,16^. Considering that saliva, whole blood and the gut are three distinct niches, it seems reasonable that there would be discrepancies in differences to the microbial α-diversity associated with schizophrenia.

The two dominant bacterial phyla in the present study were Proteobacteria and Firmicutes, which is in accordance with the predominant salivary microbes in humans reported previously^34^. These phyla exhibited completely divergent change trends in a stepwise manner from HC to CHR to FES, suggesting that Firmicutes could have a competitive advantage over Proteobacteria and may synergize with Actinobacteria, Fusobacteria, and Acidobacteria in niche occupancy during initiation of schizophrenia. Because this was an observational study, we could not determine which of the altered phyla were directly affected by schizophrenia or indirectly affected through microbe–microbe interactions. Interestingly, the enrichment of Firmicutes and depletion of Proteobacteria observed in FES patients in the present study has also been reported in the salivary microbiome of patients with primary Sjögren’s syndrome, which is a systemic autoimmune disease involving chronic inflammation of the salivary and lacrimal glands^35^. Notably, schizophrenia is also characterized by chronic low-level inflammation^36^, so it seems that the disease-associated microbial ratios may be indicative of the inflammatory response implicated in schizophrenia. Moreover, certain salivary microbes identified in the present study displayed parallel alterations with the gut microbiota reported previously in patients with schizophrenia, such as depleted *Haemophilus*^16^, and enriched *Megasphaera*^12,14^ and *Fusobacterium*^14^, inferring possible microbial communication between the oral cavity and the gut. *Haemophilus* is adjusted to survive in oxidative stress environments^37^ and has been reported to contribute to oral dysbiosis in patients with inflammatory bowel disease^38^.

In the present study, H_2_S-producing bacteria mainly referred to sulfate-reducing bacteria (SRB), which attracted our attention due to their marked enrichment in the disease status. SRBs are associated with inflammatory diseases, e.g. inflammatory bowel disease^39^ and periodontitis^40^, which are the result of interactions between the microbiota and the host’s immune system. The enrichment of SRBs in the FES and CHR groups hints that SRBs might affect schizophrenia through the low-grade inflammation response mentioned above. Cysteine metabolism produces taurine and sulfate, which SRB catabolize into H_2_S. We identified an upregulated pathway of cysteine and methionine metabolism (Supplementary Table 4) in the present study, which supports the observed enrichment of SRBs in FES and CHR groups compared to in HCs. Furthermore, as the main final product of sulfate-reducing bacterial metabolism, H_2_S can protect neurons from oxidative stress^41^. However, excess H_2_S and polysulfide production is implicated in the pathophysiology of schizophrenia^42^. This means that H_2_S-producing bacteria and their product H_2_S are associated with schizophrenia, but a causal relationship with the disease needs to be further clarified. Notably, the enrichment of H_2_S-producing bacteria might precede the onset of schizophrenia, as *Leptotrichia* and *Actinomyces* were enriched in the CHR group, and could therefore predict CHR and disease status. Given that it can be collected in an easy, non-invasive and safe manner as well as that it exhibits long-term stability, saliva offers an attractive source of microbes with diagnostic and prognostic value.

Specific salivary microbes indicate not only the single-symptomatic severity of schizophrenia or CHR but also multiple symptoms of either condition. For example, *Brevibacillus*, involved in the induction of local inflammation in mice^43^, was correlated with negative symptoms of both FES and CHR groups. In addition, salivary microbes exhibit disease-specific relationships with essential peripheral markers, extending the scope of the inflammation hypothesis for schizophrenia. Gram-negative bacteria *Campylobacter*, *Fusobacterium*, and *Haemophilus* were inversely associated with BPRS in addition to pro-inflammatory markers in the FES group, reminding that disturbances of specific Gram-negative bacteria could have an influence on the severity of schizophrenia through the production of LPS, which causes inflammation. The positive correlation of Propionibacteriaceae with CRP in FES might indicate an anti-inflammatory response due to Propionibacteriaceae’s potential anti-inflammatory effect^44^. Most Actinobacteria bacteria are obligate aerobes with the capacity to scavenge ROS for survival^45^. Therefore, our finding of enriched actinobacterial taxa related to thioredoxin in FES could reflect the role of Actinobacteria taxa as redox sensors in response to host oxidative stress in schizophrenia.

Our analysis of inferred metagenomes revealed a switch towards production of BCAA and lysine in FES, while *Staphylococcus* and *Megasphaera* were enriched in FES, which has been previously reported to favor BCAA and lysine biosynthesis^46^. This could infer the accumulation of BCAAs and lysine in saliva, which might contribute to the elevated levels of serum BCAAs previously reported in patients with schizophrenia^47^. Additionally, our findings of enriched oxygen-independent metabolic pathways and increased abundances of aerobic bacteria such as *Actinomyces*, *Fusobacterium*, *Porphyromonas,* and *Veillonella* in FES are suggestive of an oral environment favouring facultative anaerobes over strict aerobes related to schizophrenia. Furthermore, as compounds associated with xenobiotic biodegradation pathways can be obtained from food, a reduced ability of oral microbes to degrade these substances may have detrimental effects in schizophrenia. Intriguingly, a multitude of altered salivary bacteria were strongly related to the enriched trigger pathway of alanine, aspartate and glutamate metabolism in FES, possibly because amino acids implicated in this pathway are the substrates and/or metabolites of these bacteria^48^. As l-arginine can destabilize oral multispecies biofilms in human saliva^49^, the depleted trigger pathway of arginine and proline metabolism in FES might in turn benefit bacterial co-aggregation during disease. Metabolic functional dysbiosis showed parallel alterations between the CHR and FES groups, hinting at probable microbial functions with general deleterious effects in schizophrenia and its putative prodrome.

This study provides a new interpretative frame for understanding the microbial dysbiosis associated with schizophrenia. We hypothesize that dysbiotic oral microbiota will lead to the disturbance of microbial metabolites, and the abnormal metabolites might reach the brain via possible different routes, such as the olfactory tract and the systemic circulation through the blood–brain barrier, causing a chemical and/or redox imbalance in the brain, and finally promote the initiation of schizophrenia. Therefore, we suggest that alterations in the oral microbiota could be considered as interventional and therapeutic strategies for treating or preventing schizophrenia. The strengths of this study include the recruitment of patients with FES and CHR and the control of confounders. Nevertheless, the present study is limited in that it cannot prove the causal relationship between the salivary microbiome and schizophrenia, it lacks metagenomic data to determine the actual microbial gene content in the salivary microbiome, and that the dental status and oral hygiene of the participants were not sufficiently considered. Based on these promising results, multi-omics investigations of the human microbiome in patients with schizophrenia are encouraged to determine the causality between the oral microbiome and the disease, the interactions between the oral and gut microbiota, and the mutual interplay of the microbiome, bacteriophages, and mycobiome. In addition, it is important to employ statistical methods^50–52^ accounting for the large number of zeros and the compositionality inherent to marker gene sequencing data, and to use emerging versatile machine learning (ML) methods^53^ for microbial biomarker discovery to avoid common ML pitfalls^54^ in future research.

## Methods

### Study design

This study was approved by the Research Ethics Committee at the Shanghai Mental Health Centre (No. 2013-32R1) and was performed in accordance with the Declaration of Helsinki. All participants signed written informed consent forms before any procedure was carried out. Participants younger than 18 years of age had consent provided by their parents, also assented to the procedures. In total, 85 FES, 43 CHR and 80 healthy control (HC) subjects were recruited from the Shanghai Mental Health Centre early psychosis program. The FES participants met the criteria for schizophrenia or schizophreniform disorder based on the Structured Clinical Interview for DSM-IVR, were experiencing a first episode of psychosis, and did not meet the criteria for any other axis I disorder. The FES participants were administered the Brief Psychiatric Rating Scale (BPRS), Clinical Global Impressions Severity Scale (CGI-S), and Scale for the Assessment of Negative Symptoms (SANS) tests. Participants with CHR were recruited from clinic-wide questionnaire screening using the Structured Interview of Prodrome Syndromes (SIPS)^55^. The CHR participants met at least one of the prodromal syndrome criteria-brief intermittent psychotic syndrome, attenuated positive symptom syndrome as well as genetic risk and deterioration syndrome, and did not meet the criteria for severe somatic diseases, mental retardation, or dementia^56^. HCs were assessed using the Structured Clinical Interview for DSM-IV (non-patient version) to exclude any axis I disorder. Eighty-five FES patients and 35 CHR subjects were psychotropic medication naïve and all participants were free of substance abuse, suicidal ideation, and unstable medical illness. Detailed demographic information is shown in Table 1 and Supplementary Note 4.

**Table 1:** Demographic characteristics of the study populations.

Variables	Total no.	FES	Total no.	CHR	Total no.	HC	*P* value
Gender (M/F)	85	49/36	43	31/12	80	39/41	0.044^a^
Age, mean (SD), years	85	25.66 (7.35)	43	17.40 (2.34)	80	22.88 (6.45)	<0.001^b^
Education level, mean (SD), years	85	12.76 (2.89)	43	10.44 (3.17)	80	12.01 (2.46)	<0.001^c^
Tobacco use, *n* (%)	85	0	43	0	80	3 (4)	0.165^d^
Alcohol use, *n* (%)	85	0	43	0	80	1 (1)	0.591^d^
CRP, mean (SD), pg/mL	47	8074.54 (1770.59)	NA	NA	50	7715.98 (1515.8)	0.291^e^
IFNγ, mean (SD), pg/mL	47	100.32 (53.96)	NA	NA	50	152.37 (238.16)	0.148^e^
IL-1β, mean (SD), pg/mL	47	154.87 (202.29)	NA	NA	50	243.97 (421.39)	0.197^e^
IL-8, mean (SD), pg/mL	47	71.63 (34.50)	NA	NA	50	67.24 (24.90)	0.477^e^
TNFα, mean (SD), pg/mL	47	93.04 (19.35)	NA	NA	50	95.73 (49.52)	0.731^e^
S100B, mean (SD), pg/mL	47	197.70 (189.00)	NA	NA	50	255.90 (430.21)	0.401^e^
Thioredoxin, mean (SD), pg/mL	47	700.22 (1507.19)	NA	NA	50	1142.43 (2629.57)	0.321^e^
Symptoms							
BPRS							
Total score, mean (SD)	61	46.36 (9.99)	NA	NA	NA	NA	NA
CGI							
Severity of illness score, mean (SD)	80	5.11 (0.69)	NA	NA	NA	NA	NA
SANS							
Composite score, mean (SD)	61	25.77 (20.25)	NA	NA	NA	NA	NA
Affective flattening or blunting score, mean (SD)	61	1.11 (1.30)	NA	NA	NA	NA	NA
Alogia, mean (SD)	61	0.98 (1.20)	NA	NA	NA	NA	NA
Avolition—apathy, mean (SD)	61	1.74 (1.40)	NA	NA	NA	NA	NA
Anhedonia—asociality, mean (SD)	61	1.74 (1.34)	NA	NA	NA	NA	NA
Attention, mean (SD)	61	0.70 (1.01)	NA	NA	NA	NA	NA
SIPS							
Total score, mean (SD)	NA	NA	43	37.09 (11.49)	NA	NA	NA
Positive symptom score, mean (SD)	NA	NA	43	8.58 (3.53)	NA	NA	NA
Negative symptom score, mean (SD)	NA	NA	43	13.21 (6.47)	NA	NA	NA
Disorganized symptom score, mean (SD)	NA	NA	43	6.65 (3.37)	NA	NA	NA
General symptom score, mean (SD)	NA	NA	43	8.65 (3.25)	NA	NA	NA

Notes: ^a^Chi-square analysis. ^b^Kruskal–Wallis test. ^c^Analysis of variance, ANOVA. ^d^ Fisher’s exact test. ^e^Student’s *t* test.

### Sample collection

Participants were asked to collect saliva samples using a sterile collection tube in the morning, while refraining from eating, drinking, and brushing their teeth 1 h prior to collection. Whole-blood samples were collected following saliva collection after overnight fasting. Plasma samples were separated by centrifugation at 10,600 × *g* for 10 min. Saliva and plasma samples were stored at −80 °C until further analysis.

### DNA extraction and 16S rRNA gene amplicon sequencing

Genomic DNA was extracted using the Gentra Puregene Blood Kit (Qiagen, Valencia, CA, USA) according to the manufacturer’s instructions. 16S rRNA gene amplicon sequencing libraries of the V4 region were constructed using primers 515F and 806R, and pools of amplicons were sequenced on a HiSeq 2500 sequencing system (Illumina, San Diego, CA, USA) using 2 × 250 base pair chemistry.

### 16S rRNA gene sequence analysis

Paired-end reads from the original DNA fragments were merged using FLASH v1.2.7^57^. Sequences were analyzed using Quantitative Insights Into Microbial Ecology software package (QIIME) v1.7.0^58^. Reads were truncated if more than three consecutive bases did not reach the minimum Phred quality score of 19 for Q20, and reads were discarded if the post-trimming read length dropped to less than 0.75 of the initial length, or if they were shorter than 50 bp, containing ambiguous base calls or barcode/primer errors. Chimeric sequences were checked by UCHIME v4.2^59^ and removed from subsequent analyses. The resulting high-quality sequences were assigned to the same OTUs at 97% similarity using Uparse v7.0.1001^60^. Taxonomy was assigned to each OTU representative sequence using the RDP classifier v2.7^61^. The final dataset retained 14,322,556 sequences (mean ± s.d.: 68,858 ± 6,357 sequences per sample; minimum/maximum = 48,993/79,793) and contained 6,892 OTUs. To evaluate the adequacy of sequencing depth, rarefaction analysis was performed based on the number of sequences and OTUs for each sample, and the sequencing depth used for rarefaction was 48,682. Alpha diversity was assessed using the species diversity indices (Shannon) and species richness indices (Ace and Chao1). Beta diversity was assessed using the weighted UniFrac distances. The phylogenetic tree was built by the QIIME script (make_phylogeny.py). Bacterial metagenome content was predicted from 16S rRNA gene-based microbial compositions, and functional annotations were made from the KEGG catalog^62^ using the PICRUSt algorithm^63^.

### Measurement for peripheral blood markers and L-aspartate

The human acute phase protein CRP, and cytokines IFNγ, IL-1β, IL-8, TNFα, and S100B, were quantified using the customized human cytokine antibody array (RayBiotech, Norcross, GA, USA). The signals were detected using a GenePix 4000B system (Axon Instruments, Foster City, CA, USA) and analyzed using GenePix Pro 6.0 software (Axon Instruments, Foster City, CA, USA). Thioredoxin was measured using ELISA kits (MyBioSource, San Diego, CA, USA). l-Aspartate was measured using a specific kit (BioVision, San Francisco, CA, USA). All tests were performed according to the manufacturer’s instructions.

### Statistical analysis

Permutational MANOVA (PERMANOVA, ‘Adonis’ function, vegan package, R) of the weighted UniFrac distances was used to test differences in overall salivary microbiome composition^64^. A taxon was included in the analysis if it was present in more than 10% of samples at an abundance of at least 0.001%. Relative abundances of bacterial taxa and α diversity indices were compared between FES, CHR, and HC groups using the Kruskal–Wallis and Jonckheere–Terpstra tests. Sequence count data of bacterial taxa were compared between the three groups using ANCOM-BC^50^, controlling for age, gender, and education level. Quantile regression was performed for the pairwise comparisons of relative abundances of taxa between two groups, adjusting for age, gender, and education level. Spearman’s partial correlation coefficient was used to test the associations between taxa and symptomatic severity, blood markers and predicted KEGG pathways. The “DESeq” function in DESeq2 was used to test for differentially abundant KEGG pathways. Logistic regression models were constructed based on the arcsine square root transformed abundance of the genera^65^ and an area under the curve (AUC) was calculated to evaluate the performance of the fitted logistic regression models. A *p* value from PERMANOVA or Spearman’s partial correlation analysis of <0.05 was considered statistically significant, and a false discovery rate (FDR)-adjusted *q* value from the Kruskal–Wallis test, quantile regression, or DESeq2 < 0.05 was considered statistically significant. All analyses were carried out using SPSS 24 and R 3.4.4.

### Reporting summary

Further information on research design is available in the Nature Research Reporting Summary linked to this article.

## Supplementary information

Supplementary Information

Reporting Summary

## Data Availability

All sequencing data associated with this study are publicly available in the NCBI SRA database (accession number PRJNA647054). All relevant data are available from the authors upon reasonable request. The codes that support the findings of this study are available from the corresponding author upon reasonable request.

## References

[1] Disease GBD, Injury I, Prevalence C (2017). Global, regional, and national incidence, prevalence, and years lived with disability for 328 diseases and injuries for 195 countries, 1990-2016: a systematic analysis for the Global Burden of Disease Study 2016. Lancet.

[2] Tseng HH (2018). Nigral stress-induced dopamine release in clinical high risk and antipsychotic-naive schizophrenia. Schizophr. Bull..

[3] Fusar-Poli P (2012). Predicting psychosis meta-analysis of transition outcomes in individuals at high clinical risk. Arch. Gen. Psychiatry.

[4] Nguyen TT (2021). Gut microbiome in schizophrenia: altered functional pathways related to immune modulation and atherosclerotic risk. Brain Behav. Immun..

[5] Yolken R, Prandovszky E, Severance EG, Hatfield G, Dickerson F (2020). The oropharyngeal microbiome is altered in individuals with schizophrenia and mania. Schizophrenia Res..

[6] Li S (2020). Altered gut microbiota associated with symptom severity in schizophrenia. PeerJ.

[7] Pan R (2020). Analysis of the diversity of intestinal microbiome and its potential value as a biomarker in patients with schizophrenia: a cohort study. Psychiatry Res..

[8] Chen X (2021). Profiling the differences of gut microbial structure between schizophrenia patients with and without violent behaviors based on 16S rRNA gene sequencing. Int. J. Leg. Med..

[9] Zhu F (2020). Metagenome-wide association of gut microbiome features for schizophrenia. Nat. Commun..

[10] Zheng P (2019). The gut microbiome from patients with schizophrenia modulates the glutamate-glutamine-GABA cycle and schizophrenia-relevant behaviors in mice. Sci. Adv..

[11] Zhu F (2020). Transplantation of microbiota from drug-free patients with schizophrenia causes schizophrenia-like abnormal behaviors and dysregulated kynurenine metabolism in mice. Mol. Psychiatry.

[12] Xu R (2020). Altered gut microbiota and mucosal immunity in patients with schizophrenia. Brain Behav. Immun..

[13] Yuan X (2018). Changes in metabolism and microbiota after 24-week risperidone treatment in drug naive, normal weight patients with first episode schizophrenia. Schizophr. Res..

[14] Shen Y (2018). Analysis of gut microbiota diversity and auxiliary diagnosis as a biomarker in patients with schizophrenia: a cross-sectional study. Schizophr. Res..

[15] Schwarz E (2018). Analysis of microbiota in first episode psychosis identifies preliminary associations with symptom severity and treatment response. Schizophr. Res..

[16] Nguyen TT (2019). Differences in gut microbiome composition between persons with chronic schizophrenia and healthy comparison subjects. Schizophr. Res..

[17] Yolken RH (2015). Metagenomic sequencing indicates that the oropharyngeal phageome of individuals with schizophrenia differs from that of controls. Schizophr. Bull..

[18] Castro-Nallar E (2015). Composition, taxonomy and functional diversity of the oropharynx microbiome in individuals with schizophrenia and controls. PeerJ.

[19] Li S (2021). The gut microbiome is associated with brain structure and function in schizophrenia. Sci. Rep..

[20] Escapa, I. F. et al. New insights into human nostril microbiome from the Expanded Human Oral Microbiome Database (eHOMD): a resource for the microbiome of the human aerodigestive tract. *mSystems* **3**, 10.1128/mSystems.00187-18 (2018). 10.1128/mSystems.00187-18 PMC6280432 30534599

[21] Human Microbiome Project, C. (2012). Structure, function and diversity of the healthy human microbiome. Nature.

[22] Gao L (2018). Oral microbiomes: more and more importance in oral cavity and whole body. Protein Cell.

[23] Olsen I, Singhrao SK (2015). Can oral infection be a risk factor for Alzheimer’s disease?. J. Oral. Microbiol.

[24] Ranjan R, Abhinay A, Mishra M (2018). Can oral microbial infections be a risk factor for neurodegeneration? A review of the literature. Neurol. India.

[25] Simpson, C. A. et al. Oral microbiome composition, but not diversity, is associated with adolescent anxiety and depression symptoms. *Physiol. Behav.* **226**, 10.1016/j.physbeh.2020.113126 (2020). 10.1016/j.physbeh.2020.113126 32777312

[26] Qiao, Y. A. et al. Alterations of oral microbiota distinguish children with autism spectrum disorders from healthy controls. *Sci. Rep.* **8**, 10.1038/s41598-018-19982-y (2018). 10.1038/s41598-018-19982-y PMC5785483 29371629

[27] Kong, X. J. et al. New and preliminary evidence on altered oral and gut microbiota in individuals with autism spectrum disorder (ASD): implications for ASD diagnosis and subtyping based on microbial biomarkers. *Nutrients* **11**, 10.3390/nu11092128 (2019). 10.3390/nu11092128 PMC6770733 31489949

[28] Hicks SD (2018). Oral microbiome activity in children with autism spectrum disorder. Autism Res..

[29] Belstrom D (2016). Temporal stability of the salivary microbiota in oral health. PLoS ONE.

[30] Marsh PD, Do T, Beighton D, Devine DA (2016). Influence of saliva on the oral microbiota. Periodontol 2000.

[31] Sharon G, Sampson TR, Geschwind DH, Mazmanian SK (2016). The central nervous system and the gut microbiome. Cell.

[32] Olde Loohuis LM (2018). Transcriptome analysis in whole blood reveals increased microbial diversity in schizophrenia. Transl. Psychiatry.

[33] Potgieter M, Bester J, Kell DB, Pretorius E (2015). The dormant blood microbiome in chronic, inflammatory diseases. FEMS Microbiol. Rev..

[34] Mosaddad SA (2019). Oral microbial biofilms: an update. Eur. J. Clin. Microbiol. Infect. Dis..

[35] Zhou S, Cai Y, Wang M, Yang WD, Duan N (2018). Oral microbial flora of patients with Sicca syndrome. Mol. Med. Rep..

[36] Muller N (2018). Inflammation in schizophrenia: pathogenetic aspects and therapeutic considerations. Schizophr. Bull..

[37] Harrison A, Bakaletz LO, Munson RS (2012). Haemophilus influenzae and oxidative stress. Front. Cell Infect. Microbiol..

[38] Said HS (2014). Dysbiosis of salivary microbiota in inflammatory bowel disease and its association with oral immunological biomarkers. DNA Res..

[39] Dordevic D, Jancikova S, Vitezova M, Kushkevych I (2021). Hydrogen sulfide toxicity in the gut environment: meta-analysis of sulfate-reducing and lactic acid bacteria in inflammatory processes. J. Adv. Res..

[40] Kushkevych, I., Coufalova, M., Vitezova, M. & Rittmann, S. K. M. R. Sulfate-reducing bacteria of the oral cavity and their relation with periodontitis-recent advances. *J. Clin. Med*. **9**, 10.3390/jcm9082347 (2020). 10.3390/jcm9082347 PMC7464432 32717883

[41] Kimura Y, Kimura H (2004). Hydrogen sulfide protects neurons from oxidative stress. FASEB J..

[42] Ide, M. et al. Excess hydrogen sulfide and polysulfides production underlies a schizophrenia pathophysiology. *EMBO Mol. Med.* **11**, 10.15252/emmm.201910695 (2019). 10.15252/emmm.201910695 PMC6895609 31657521

[43] Monteon V, May-Gil I, Nunez-Oreza L, Lopez R (2018). Feces from wild Triatoma dimidiata induces local inflammation and specific immune response in a murine model. Ann. Parasitol..

[44] Rogers MAM, Aronoff DM (2016). The influence of non-steroidal anti-inflammatory drugs on the gut microbiome. Clin. Microbiol. Infect..

[45] den Hengst CD, Buttner MJ (2008). Redox control in actinobacteria. Biochim. Biophys. Acta.

[46] Dai ZL, Wu GY, Zhu WY (2011). Amino acid metabolism in intestinal bacteria: links between gut ecology and host health. Front. Biosci. Landmark.

[47] Oresic, M. et al. Metabolome in schizophrenia and other psychotic disorders: a general population-based study. *Genome Med* **3**, 10.1186/gm233 (2011). 10.1186/gm233 PMC3092104 21429189

[48] Neis EPJG, Dejong CHC, Rensen SS (2015). The role of microbial amino acid metabolism in host metabolism. Nutrients.

[49] Kolderman, E. et al. L-arginine destabilizes oral multi-species biofilm communities developed in human saliva. *PLoS ONE* **10**, 10.1371/journal.pone.0121835 (2015). 10.1371/journal.pone.0121835 PMC4422691 25946040

[50] Lin, H. & Das Peddada, S. Analysis of compositions of microbiomes with bias correction. *Nat. Commun.* **11**, 10.1038/s41467-020-17041-7 (2020). 10.1038/s41467-020-17041-7 PMC7360769 32665548

[51] Mandal S (2015). Analysis of composition of microbiomes: a novel method for studying microbial composition. Micro. Ecol. Health Dis..

[52] Weiss, S. et al. Normalization and microbial differential abundance strategies depend upon data characteristics. *Microbiome* **5**, 10.1186/s40168-017-0237-y (2017). 10.1186/s40168-017-0237-y PMC5335496 28253908

[53] Wirbel J (2021). Microbiome meta-analysis and cross-disease comparison enabled by the SIAMCAT machine learning toolbox. Genome Biol..

[54] Quinn, T. P. Stool studies don’t pass the sniff test: a systematic review of human gut microbiome research suggests widespread misuse of machine learning. Preprint at https://arxiv.org/abs/2107.03611 (2021).

[55] Miller TJ (2003). Prodromal assessment with the structured interview for prodromal syndromes and the scale of prodromal symptoms: predictive validity, interrater reliability, and training to reliability. Schizophr. Bull..

[56] Zhang TH (2017). Two-year follow-up of a Chinese sample at clinical high risk for psychosis: timeline of symptoms, help-seeking and conversion. Epidemiol. Psychiatr. Sci..

[57] Magoc T, Salzberg SL (2011). FLASH: fast length adjustment of short reads to improve genome assemblies. Bioinformatics.

[58] Caporaso JG (2010). QIIME allows analysis of high-throughput community sequencing data. Nat. Methods.

[59] Edgar RC, Haas BJ, Clemente JC, Quince C, Knight R (2011). UCHIME improves sensitivity and speed of chimera detection. Bioinformatics.

[60] Edgar RC (2013). UPARSE: highly accurate OTU sequences from microbial amplicon reads. Nat. Methods.

[61] Wang Q, Garrity GM, Tiedje JM, Cole JR (2007). Naive Bayesian classifier for rapid assignment of rRNA sequences into the new bacterial taxonomy. Appl Environ. Microbiol.

[62] Kanehisa M, Goto S, Sato Y, Furumichi M, Tanabe M (2012). KEGG for integration and interpretation of large-scale molecular data sets. Nucleic Acids Res..

[63] Langille MGI (2013). Predictive functional profiling of microbial communities using 16S rRNA marker gene sequences. Nat. Biotechnol..

[64] McArdle BH, Anderson MJ (2001). Fitting multivariate models to community data: a comment on distance-based redundancy analysis. Ecology.

[65] Ruhlemann MC (2017). Faecal microbiota profiles as diagnostic biomarkers in primary sclerosing cholangitis. Gut.

