# Supplementary material for: Salivary microbiome profiling reveals a dysbiotic schizophrenia-associated microbiota
Source: NPJ Schizophr. 2021 Oct 28;7:51. doi: 10.1038/s41537-021-00180-1 (PMC8553823; doi:10.1038/s41537-021-00180-1)
Supplement: Supplementary file 1 — Supplementary Information [file 41537_2021_180_MOESM1_ESM.pdf]

Salivary microbiome profiling reveals a dysbiotic schizophrenia-associated microbiota

Authors

Ying Qing[1]; Lihua Xu[2]; Gaoping Cui[1]; Liya Sun[1]; Xiaowen Hu[1]; Xuhan Yang[1]; Jie Jiang[1]; Juan Zhang[1]; Tianhong Zhang[2]; Tao Wang[3,4]; Lin He[1]; Jijun Wang[2,*]; Chunling Wan[1,*].

[*] Corresponding authors: Chunling Wan and Jijun Wang.

Author information

[1] Bio-X Institutes, Key Laboratory for the Genetics of Developmental and Neuropsychiatric Disorders, Ministry of Education, Shanghai Jiao Tong University, Shanghai, China.

[2] Shanghai Mental Health Centre, Shanghai Jiao Tong University School of Medicine, Shanghai Key Laboratory of Psychotic Disorders, Shanghai, China.

[3] Department of Bioinformatics and Biostatistics, Shanghai Jiao Tong University, Shanghai, China.

[4] SJTU-Yale Joint Center for Biostatistics and Data Science, Shanghai Jiao Tong University, Shanghai, China.

Supplementary material:

Supplementary Notes 1-4

Supplementary Tables 1-4

Supplementary Figures 1-6

Supplementary Notes

Supplementary Note 1. The salivary microbiota displays stage-specific alterations in schizophrenia

Most noticeably, Proteobacteria and Firmicutes, which largely dominated the microbial communities in saliva, differed significantly following disease onset and displayed the reverse alterations in a stepwise manner from HC to CHR to FES (Figure 2b, Supplementary Table 1). Proteobacteria was gradually depleted in the FES group relative to the CHR group (10.7%) and HCs (33.4%), whereas Firmicutes was progressively enriched in the FES group compared to the CHR group (24.4%) and HCs (62.6%). Bacteroidetes, as the third most abundant phylum, manifested a unique change trend in the FES group, exhibiting depletion relative to the CHR group (23.5%) but enrichment relative to HCs (80.4%) (Figure 2b). At the genus level, the salivary microbiota was dominated by *Neisseria* and *Streptococcus* (Supplementary Figure 3a), and was mainly composed of gram-negative bacteria (Supplementary Figure 3b), but no significant difference in the ratio of gram-positive bacteria to gram-negative bacteria was observed in the FES group compare to HCs (data not shown). The results of comparative taxonomic analyses among the three groups are shown in Supplementary Table 1.

For the CHR group, the alterations in salivary bacterial abundances were greatly different from those of the FES group, compared to HCs, illustrating the stage-specific microbial changes associated with progression to schizophrenia.

Supplementary Note 2. The ratios of Actinobacteria/Proteobacteria and Bacteroidetes/Proteobacteria are associated with the clinical characteristics of schizophrenia and the prodromal psychosis stage

The Actinobacteria/Proteobacteria ratio was negatively related to CRP in HCs but not in the FES group (Supplementary Figure 4b). In addition, we also found that the Bacteroidetes/Proteobacteria ratio was negatively correlated with TNFα in the FES group rather than the HCs (Supplementary Figure 4b). Moreover, the Actinobacteria/Proteobacteria ratio was negatively correlated with thioredoxin in the FES group (Supplementary Figure 4b).

Supplementary Note 3. The functional dysbiosis of "metabolism" in the salivary microbiome is particularly predominant in schizophrenia

A total of 296 non-human KEGG pathways were included in the functional analysis. One hundred and eighty-one out of 296 (61.1%) KEGG pathways differed in abundance when the FES group was compared to HCs, among which 57.5% of the total differentially abundant pathways corresponded to the term "metabolism" (Figure 4a and Supplementary Table 4). In addition, there were 119 differentially abundant pathways in CHR versus HC (Supplementary Figure 5), among which 101, mainly classified as "metabolism", overlapped between the FES and CHR groups compared to the HCs (Supplementary Figure 6), reflecting similar changes in salivary microbial metabolic functions between psychotic and pre-psychotic status. Moreover, differentially abundant KEGG pathways between CHR and HC and FES and CHR are shown in Supplementary Table 4.

Supplementary Note 4. Demographic and clinical information of recruited subjects

In total, 208 individuals from 14 to 46 years of age were included in this case-control study and categorized into 3 groups: 85 subjects with a well-established diagnosis of schizophrenia, 43 subjects at the clinical high-risk (CHR) state and 80 healthy controls (HCs). All 85 patients with schizophrenia were in their first episode and were drug-naïve, and 35 CHR subjects were anti-psychotic naïve, while the other 8 CHR subjects were treated from 2 days to 3 months (1 for 2 days, 2 for 1week and 1 for 2/3/4 weeks and 2/3 months). All 208 subjects completed saliva collection, among which 47 FES patients and 50 HCs completed blood draw. Data of the CGI-S scores were collected on 80 FES patients, while BPRS and SANS scores on 61 FES patients and SIPS scores on 43 CHR subjects. Considering the differences in age, gender and education level among the three groups (Table 1), all statistical tests were adjusted for these confounders.

Supplementary Tables

Supplementary Table 1. Q-values for comparisons of median relative abundances of salivary taxa in FES, CHR and HC groups.

Taxa	Taxonomic Assignment	Relative abundance			Three groups			FES vs HC		CHR vs HC		FES vs CHR	
		FES	CHR	HC	q-value[1]	q-value[2]	q-value[3]	q-value[4]	FC	q-value[4]	FC	q-value[4]	FC
Acidobacteria	p__Acidobacteria;	0.000205	0.000144	0.000123	8.53E-03	1.55E-03	4.18E-02	1.85E-02	1.67	3.35E-01	1.17	9.31E-02	1.43
Holophagae	p__Acidobacteria;c__Holophagae;	4.11E-05	2.05E-05	4.11E-05	1.45E-01	9.43E-01	1	9.22E-01	1.00	1	0.50	1	2.00
Subgroup 10	p__Acidobacteria;c__Holophagae;o__Subgroup_10;	6.16E-05	2.05E-05	4.11E-05	1.48E-01	6.33E-01	1	1	1.50	1	0.50	1	3.00
ABS-19	p__Acidobacteria;c__Holophagae;o__Subgroup_10;f__ABS-19;	6.16E-05	2.05E-05	4.11E-05	3.88E-01	7.11E-01	1	1	1.50	1	0.50	8.35E-01	3.00
Subgroup 7	p__Acidobacteria;c__Holophagae;o__Subgroup_7;	6.16E-05	2.05E-05	4.11E-05	4.47E-01	8.32E-01	1	9.19E-01	1.50	1	0.50	1	3.00
Subgroup 22	p__Acidobacteria;c__Subgroup_22;	3.08E-05	2.05E-05	4.11E-05	3.71E-01	5.77E-01	0	1	0.75	NA	0.50	NA	1.50
unidentified Acidobacteria	p__Acidobacteria;c__unidentified_Acidobacteria;	0.000175	0.000144	0.000123	1.03E-02	2.28E-03	6.15E-03	4.63E-03	1.42	1.47E-01	1.17	1.89E-01	1.21
Acidobacteriales	p__Acidobacteria;c__unidentified_Acidobacteria;o__Acidobacteriales;	8.22E-05	8.22E-05	4.11E-05	1.93E-01	2.66E-01	2.21E-03	2.11E-01	2.00	6.32E-01	2.00	8.03E-01	1.00
Acidobacteriaceae Subgroup 1	p__Acidobacteria;c__unidentified_Acidobacteria;o__Acido	8.22E-05	8.22E-05	4.11E-05	1.86E-01	2.76E-01	5.27E-02	1.60E-01	2.00	6.17E-01	2.00	7.92E-01	1.00

	bacteriales;f__Acidobacteriaceae_.Subgroup_1.;												
Acidobacterium	p__Acidobacteria;c__unidentified_Acidobacteria;o__Acidobacteriales;f__Acidobacteriaceae_.Subgroup_1.;g__Acidobacterium;	4.11E-05	2.05E-05	2.05E-05	3.07E-01	5.15E-01	5.82E-01	6.75E-01	2.00	1	1.00	8.62E-01	2.00
Candidatus Koribacter	p__Acidobacteria;c__unidentified_Acidobacteria;o__Acidobacteriales;f__Acidobacteriaceae_.Subgroup_1.;g__Candidatus_Koribacter;	4.11E-05	3.08E-05	2.05E-05	5.73E-01	4.02E-01	0	1	2.00	1	1.50	1	1.33
Subgroup 17	p__Acidobacteria;c__unidentified_Acidobacteria;o__Subgroup_17;	4.11E-05	2.05E-05	6.16E-05	3.03E-01	3.76E-01	0	1	0.67	NA	0.33	NA	2.00
Subgroup 2	p__Acidobacteria;c__unidentified_Acidobacteria;o__Subgroup_2;	4.11E-05	2.05E-05	4.11E-05	1.71E-01	5.32E-01	7.80E-02	1	1.00	8.00E-01	0.50	7.43E-01	2.00
Subgroup 3	p__Acidobacteria;c__unidentified_Acidobacteria;o__Subgroup_3;	6.16E-05	6.16E-05	4.11E-05	4.17E-01	2.66E-01	1	5.11E-01	1.50	1	1.50	1	1.00
unidentified Acidobacteria	p__Acidobacteria;c__unidentified_Acidobacteria;o__Subgroup_3;f__unidentified_Acidobacteria;	6.16E-05	4.11E-05	4.11E-05	2.52E-01	1.92E-01	1	2.85E-01	1.50	1	1.00	1	1.50

Bryobacter	p__Acidobacteria;c__unidentified_Acidobacteria;o__Subgroup_3;f__unidentified_Acidobacteria;g__Bryobacter;	6.16E-05	4.11E-05	4.11E-05	4.17E-01	3.31E-01	1	1	1.50	1	1.00	1	1.50
Candidatus Solibacter	p__Acidobacteria;c__unidentified_Acidobacteria;o__Subgroup_3;f__unidentified_Acidobacteria;g__Candidatus_Solibacter;	5.14E-05	3.08E-05	4.11E-05	3.94E-01	4.49E-01	1	1	1.25	1	0.75	1	1.67
Subgroup 4	p__Acidobacteria;c__unidentified_Acidobacteria;o__Subgroup_4;	4.11E-05	2.05E-05	4.11E-05	1.93E-01	2.79E-01	1	6.65E-01	1.00	7.43E-01	0.50	9.99E-01	2.00
11-24	p__Acidobacteria;c__unidentified_Acidobacteria;o__Subgroup_4;f__11-24;	4.11E-05	2.05E-05	4.11E-05	1.86E-01	7.10E-01	0	1	1.00	NA	0.50	NA	2.00
RB41	p__Acidobacteria;c__unidentified_Acidobacteria;o__Subgroup_4;f__RB41;	6.16E-05	2.05E-05	3.08E-05	1.86E-01	2.96E-01	1	4.59E-01	2.00	1	0.67	2.82E-01	3.00
unidentified Acidobacteria-2	p__Acidobacteria;c__unidentified_Acidobacteria;o__Subgroup_4;f__unidentified_Acidobacteria;	4.11E-05	4.11E-05	2.05E-05	8.31E-01	6.98E-01	1	1	2.00	1	2.00	7.72E-01	1.00
Blastocatella	p__Acidobacteria;c__unidentified_Acidobacteria;o__Subgroup_4;f__unidentified_Acidobacteria;g__Blastocatella;	4.11E-05	4.11E-05	2.05E-05	8.29E-01	6.24E-01	1	9.53E-01	2.00	1	2.00	7.40E-01	1.00

Subgroup 5	p__Acidobacteria;c__unidentified_Acidobacteria;o__Subgroup_5;	4.11E-05	2.05E-05	0.000103	1.82E-01	5.32E-01	0	1	0.40	NA	0.20	NA	2.00
Subgroup 6	p__Acidobacteria;c__unidentified_Acidobacteria;o__Subgroup_6;	7.19E-05	2.05E-05	4.11E-05	1.78E-02	1.19E-01	7.80E-02	6.16E-01	1.75	1	0.50	8.03E-01	3.51
unidentified Acidobacteria-1	p__Acidobacteria;c__unidentified_Acidobacteria;o__Subgroup_6;f__unidentified_Acidobacteria;	4.11E-05	2.05E-05	6.16E-05	4.23E-01	6.98E-01	0	1	0.67	NA	0.33	NA	2.00
unidentified Acidobacteria	p__Acidobacteria;c__unidentified_Acidobacteria;o__Subgroup_6;f__unidentified_Acidobacteria;g__unidentified_Acidobacteria;	4.11E-05	2.05E-05	6.16E-05	4.03E-01	6.25E-01	0	1	0.67	NA	0.33	NA	2.00
Actinobacteria	p__Actinobacteria;	0.05821	0.034384	0.028971	1.00E-05	1.65E-06	9.11E-04	1.58E-03	2.01	1.75E-01	1.19	4.39E-01	1.69
Acidimicrobiia	p__Actinobacteria;c__Acidimicrobiia;	6.16E-05	2.05E-05	4.11E-05	9.00E-03	1.04E-01	5.83E-01	2.52E-01	1.50	1	0.50	8.45E-01	3.00
Acidimicrobiales	p__Actinobacteria;c__Acidimicrobiia;o__Acidimicrobiales;	6.16E-05	2.05E-05	4.11E-05	8.49E-03	1.29E-01	6.98E-01	3.83E-01	1.50	7.14E-01	0.50	7.91E-01	3.00
Acidimicrobiaceae	p__Actinobacteria;c__Acidimicrobiia;o__Acidimicrobiales;f__Acidimicrobiaceae;	4.11E-05	4.11E-05	4.11E-05	9.75E-01	9.02E-01	0	1	1.00	1	1.00	1	1.00
CL500-29 marine group	p__Actinobacteria;c__Acidimicrobiia;o__Acidimicrobiales;	7.19E-05	0.000411	2.05E-05	3.23E-01	5.15E-01	0	1	3.51	NA	20.04	NA	0.18

	f__Acidimicrobiaceae;g__CL500-29_marine_group;												
Iamiaceae	p__Actinobacteria;c__Acidimicrobiia;o__Acidimicrobiales;f__Iamiaceae;	4.11E-05	0	2.05E-05	8.52E-01	9.01E-01	0	1	2.00	NA	NA	NA	NA
Iamia	p__Actinobacteria;c__Acidimicrobiia;o__Acidimicrobiales;f__Iamiaceae;g__Iamia;	4.11E-05	0	2.05E-05	8.54E-01	9.00E-01	0	1	2.00	NA	NA	NA	NA
Coriobacteriia	p__Actinobacteria;c__Coriobacteriia;	0.001356	0.000986	0.000637	3.34E-08	5.33E-09	3.94E-07	6.44E-06	2.13	1.47E-01	1.55	2.65E-01	1.37
Coriobacteriales	p__Actinobacteria;c__Coriobacteriia;o__Coriobacteriales;	0.001356	0.000986	0.000637	4.20E-08	1.01E-08	2.39E-07	1.61E-05	2.13	1.17E-01	1.55	3.87E-01	1.37
Coriobacteriaceae	p__Actinobacteria;c__Coriobacteriia;o__Coriobacteriales;f__Coriobacteriaceae;	0.001356	0.000986	0.000637	3.45E-08	8.25E-09	7.98E-07	9.33E-06	2.13	2.57E-01	1.55	3.04E-01	1.37
Atopobium	p__Actinobacteria;c__Coriobacteriia;o__Coriobacteriales;f__Coriobacteriaceae;g__Atopobium;	0.00113	0.000863	0.000555	1.35E-07	5.81E-08	4.78E-07	1.83E-04	2.04	1.89E-01	1.56	9.91E-01	1.31
Cryptobacterium	p__Actinobacteria;c__Coriobacteriia;o__Coriobacteriales;f__Coriobacteriaceae;g__Cryptobacterium;	2.05E-05	2.05E-05	2.05E-05	8.63E-01	1	1	1	1.00	1	1.00	1	1.00
Olsenella	p__Actinobacteria;c__Coriobacteriia;o__Coriobacteriales;f	6.16E-05	6.16E-05	6.16E-05	3.38E-01	2.08E-01	1	3.03E-01	1.00	9.73E-01	1.00	1	1.00

	__Coriobacteriaceae;g__Olsenella;												
Slackia	p__Actinobacteria;c__Coriobacteriia;o__Coriobacteriales;f__Coriobacteriaceae;g__Slackia;	4.11E-05	4.11E-05	2.05E-05	2.46E-01	1.66E-01	4.00E-02	1.79E-01	2.00	1	2.00	1	1.00
MB-A2-108	p__Actinobacteria;c__MB-A2-108;	4.11E-05	0	4.11E-05	4.66E-01	5.68E-01	0	1	1.00	NA	NA	NA	NA
Rubrobacteria	p__Actinobacteria;c__Rubrobacteria;	4.11E-05	4.11E-05	4.11E-05	8.14E-01	6.25E-01	1	9.22E-01	1.00	1	1.00	1	1.00
Rubrobacterales	p__Actinobacteria;c__Rubrobacteria;o__Rubrobacterales;	4.11E-05	4.11E-05	4.11E-05	8.26E-01	7.14E-01	1	9.29E-01	1.00	8.87E-01	1.00	1	1.00
Rubrobacteriaceae	p__Actinobacteria;c__Rubrobacteria;o__Rubrobacterales;f__Rubrobacteriaceae;	4.11E-05	4.11E-05	4.11E-05	8.31E-01	6.98E-01	1	9.63E-01	1.00	9.70E-01	1.00	1	1.00
Rubrobacter	p__Actinobacteria;c__Rubrobacteria;o__Rubrobacterales;f__Rubrobacteriaceae;g__Rubrobacter;	4.11E-05	4.11E-05	4.11E-05	8.29E-01	6.23E-01	1	9.61E-01	1.00	9.47E-01	1.00	1	1.00
Thermoleophilia	p__Actinobacteria;c__Thermoleophilia;	0.000103	4.11E-05	6.16E-05	1.59E-03	2.86E-02	4.98E-04	7.76E-02	1.67	1	0.67	4.83E-02	2.50
Gaiellales	p__Actinobacteria;c__Thermoleophilia;o__Gaiellales;	6.16E-05	2.05E-05	6.16E-05	3.03E-02	7.44E-01	4.53E-01	8.71E-01	1.00	7.83E-01	0.33	7.91E-01	3.00
Gaiellaceae	p__Actinobacteria;c__Thermoleophilia;o__Gaiellales;f__Gaiellaceae;	4.11E-05	2.05E-05	5.14E-05	2.29E-01	9.18E-01	1	1	0.80	1	0.40	1	2.00

Gaiella	p__Actinobacteria;c__Thermoleophilia;o__Gaiellales;f__Gaiellaceae;g__Gaiella;	4.11E-05	2.05E-05	5.14E-05	2.08E-01	9.33E-01	1	1	0.80	1	0.40	1	2.00
unidentified Gaiellales	p__Actinobacteria;c__Thermoleophilia;o__Gaiellales;f__unidentified_Gaiellales;	4.11E-05	2.05E-05	2.05E-05	1.90E-01	3.73E-01	1	1	2.00	1	1.00	1	2.00
unidentified Gaiellales	p__Actinobacteria;c__Thermoleophilia;o__Gaiellales;f__unidentified_Gaiellales;g__unidentified_Gaiellales;	4.11E-05	2.05E-05	2.05E-05	1.77E-01	3.31E-01	1	1	2.00	1	1.00	1	2.00
Solirubrobacterales	p__Actinobacteria;c__Thermoleophilia;o__Solirubrobacterales;	8.22E-05	4.11E-05	6.16E-05	4.86E-02	1.69E-01	1.96E-04	2.11E-01	1.33	7.74E-01	0.67	2.51E-01	2.00
0319-6M6	p__Actinobacteria;c__Thermoleophilia;o__Solirubrobacterales;f__0319-6M6;	6.16E-05	2.05E-05	4.11E-05	3.87E-01	4.32E-01	0	1	1.50	NA	0.50	NA	3.00
288-2	p__Actinobacteria;c__Thermoleophilia;o__Solirubrobacterales;f__288-2;	4.11E-05	5.14E-05	2.05E-05	5.99E-01	6.84E-01	0	1	2.00	1	2.50	1	0.80
480-2	p__Actinobacteria;c__Thermoleophilia;o__Solirubrobacterales;f__480-2;	5.14E-05	4.11E-05	4.11E-05	9.68E-01	8.32E-01	1	1	1.25	NA	1.00	NA	1.25
Patulibacteraceae	p__Actinobacteria;c__Thermoleophilia;o__Solirubrobacterales;f__Patulibacteraceae;	5.14E-05	2.05E-05	6.16E-05	5.77E-01	7.89E-01	0	1	0.83	NA	0.33	NA	2.50

Patulibacter	p__Actinobacteria;c__Thermoleophilia;o__Solirubrobacterales;f__Patulibacteraceae;g__Patulibacter;	5.14E-05	2.05E-05	6.16E-05	5.70E-01	7.39E-01	0	9.68E-01	0.83	NA	0.33	NA	2.50
Solirubrobacteraceae	p__Actinobacteria;c__Thermoleophilia;o__Solirubrobacterales;f__Solirubrobacteraceae;	4.11E-05	4.11E-05	4.11E-05	9.90E-01	9.57E-01	8.34E-01	1	1.00	1	1.00	1	1.00
Solirubrobacter	p__Actinobacteria;c__Thermoleophilia;o__Solirubrobacterales;f__Solirubrobacteraceae;g__Solirubrobacter;	4.11E-05	4.11E-05	4.11E-05	9.94E-01	9.78E-01	1	9.65E-01	1.00	1	1.00	1	1.00
YNPFFP1	p__Actinobacteria;c__Thermoleophilia;o__Solirubrobacterales;f__YNPFFP1;	4.11E-05	2.05E-05	4.11E-05	2.99E-01	7.85E-01	1	1	1.00	1	0.50	8.35E-01	2.00
unidentified Actinobacteria	p__Actinobacteria;c__unidentified_Actinobacteria;	0.054841	0.032699	0.02588	9.30E-06	1.59E-06	2.20E-04	5.33E-04	2.12	1.47E-01	1.26	4.53E-01	1.68
Actinomycetales	p__Actinobacteria;c__unidentified_Actinobacteria;o__Actinomycetales;	0.013659	0.01333	0.005607	1.08E-05	2.02E-05	3.76E-05	4.40E-04	2.44	3.67E-03	2.38	1	1.02
Actinomycetaceae	p__Actinobacteria;c__unidentified_Actinobacteria;o__Actinomycetales;f__Actinomycetaceae;	0.013659	0.01333	0.005607	8.74E-06	2.06E-05	2.52E-04	7.64E-04	2.44	8.22E-03	2.38	1	1.02
Actinomyces	p__Actinobacteria;c__unidentified_Actinobacteria;o__Acti	0.009736	0.012262	0.005351	6.47E-05	1.33E-03	1.57E-04	4.23E-04	1.82	4.82E-03	2.29	3.07E-01	0.79

	nomycetales;f__Actinomycetaceae;g__Actinomyces;												
Mobiluncus	p__Actinobacteria;c__unidentified_Actinobacteria;o__Actinomycetales;f__Actinomycetaceae;g__Mobiluncus;	0.001191	0.000226	0.000298	2.91E-09	2.84E-07	4.14E-11	2.88E-02	4.00	1	0.76	3.16E-01	5.27
Bifidobacteriales	p__Actinobacteria;c__unidentified_Actinobacteria;o__Bifidobacteriales;	0.000123	0.000113	9.24E-05	2.21E-01	1.29E-01	1	2.66E-01	1.33	7.14E-01	1.22	8.20E-01	1.09
Bifidobacteriaceae	p__Actinobacteria;c__unidentified_Actinobacteria;o__Bifidobacteriales;f__Bifidobacteriaceae;	0.000123	0.000113	9.24E-05	2.18E-01	1.24E-01	1	2.65E-01	1.33	7.76E-01	1.22	8.35E-01	1.09
Bifidobacterium	p__Actinobacteria;c__unidentified_Actinobacteria;o__Bifidobacteriales;f__Bifidobacteriaceae;g__Bifidobacterium;	4.11E-05	4.11E-05	4.11E-05	7.80E-01	9.95E-01	1	1	1.00	1	1.00	1	1.00
Scardovia	p__Actinobacteria;c__unidentified_Actinobacteria;o__Bifidobacteriales;f__Bifidobacteriaceae;g__Scardovia;	6.16E-05	8.22E-05	6.16E-05	2.56E-01	5.88E-01	4.65E-02	1	1.00	3.62E-01	1.33	1	0.75
Corynebacteriales	p__Actinobacteria;c__unidentified_Actinobacteria;o__Corynebacteriales;	0.001315	0.001294	0.001119	2.65E-01	1.49E-01	1	7.12E-01	1.17	1.85E-01	1.16	8.20E-01	1.02
Corynebacteriaceae	p__Actinobacteria;c__unidentified_Actinobacteria;o__Cory	0.001212	0.001171	0.000996	2.68E-01	1.60E-01	1	5.49E-01	1.22	4.75E-01	1.18	7.92E-01	1.04

	nebacteriales;f__Corynebacteriaceae;												
Corynebacterium	p__Actinobacteria;c__unidentified_Actinobacteria;o__Corynebacteriales;f__Corynebacteriaceae;g__Corynebacterium;	0.001089	0.000822	0.00075	5.60E-02	3.54E-02	1	2.32E-01	1.45	1	1.10	3.08E-01	1.33
Corynebacterium 1	p__Actinobacteria;c__unidentified_Actinobacteria;o__Corynebacteriales;f__Corynebacteriaceae;g__Corynebacterium_1;	4.11E-05	5.14E-05	4.11E-05	8.58E-01	9.78E-01	1	1	1.00	1	1.25	1	0.80
Mycobacteriaceae	p__Actinobacteria;c__unidentified_Actinobacteria;o__Corynebacteriales;f__Mycobacteriaceae;	6.16E-05	4.11E-05	4.11E-05	6.72E-01	5.81E-01	1	1	1.50	1	1.00	1	1.50
Mycobacterium	p__Actinobacteria;c__unidentified_Actinobacteria;o__Corynebacteriales;f__Mycobacteriaceae;g__Mycobacterium;	6.16E-05	4.11E-05	4.11E-05	6.73E-01	5.15E-01	1	1	1.50	1	1.00	1	1.50
Nocardiaceae	p__Actinobacteria;c__unidentified_Actinobacteria;o__Corynebacteriales;f__Nocardiaceae;	4.11E-05	4.11E-05	4.11E-05	4.85E-01	3.51E-01	1	1	1.00	1	1.00	1	1.00
Rhodococcus	p__Actinobacteria;c__unidentified_Actinobacteria;o__Cory	4.11E-05	3.08E-05	4.11E-05	9.41E-01	9.67E-01	1	1	1.00	1	0.75	3.73E-01	1.33

	nebacteriales;f__Nocardiaceae;g__Rhodococcus;												
Frankiales	p__Actinobacteria;c__unidentified_Actinobacteria;o__Frankiales;	6.16E-05	4.11E-05	6.16E-05	3.70E-01	9.17E-01	4.50E-01	1	1.00	8.87E-01	0.67	7.91E-01	1.50
Acidothermaceae	p__Actinobacteria;c__unidentified_Actinobacteria;o__Frankiales;f__Acidothermaceae;	4.11E-05	4.11E-05	2.05E-05	2.01E-01	1.59E-01	1	1	2.00	1	2.00	1	1.00
Acidothermus	p__Actinobacteria;c__unidentified_Actinobacteria;o__Frankiales;f__Acidothermaceae;g__Acidothermus;	4.11E-05	4.11E-05	2.05E-05	1.85E-01	1.26E-01	1	9.65E-01	2.00	1	2.00	1	1.00
Frankiaceae	p__Actinobacteria;c__unidentified_Actinobacteria;o__Frankiales;f__Frankiaceae;	4.11E-05	6.16E-05	6.16E-05	9.41E-01	9.54E-01	0	1	0.67	1	1.00	1	0.67
Jatrophihabitans	p__Actinobacteria;c__unidentified_Actinobacteria;o__Frankiales;f__Frankiaceae;g__Jatrophihabitans;	6.17E-05	6.16E-05	4.11E-05	7.03E-01	4.83E-01	0	1	1.50	1	1.50	8.63E-01	1.00
Geodermatophilaceae	p__Actinobacteria;c__unidentified_Actinobacteria;o__Frankiales;f__Geodermatophilaceae;	6.16E-05	4.11E-05	4.11E-05	5.96E-01	7.85E-01	1	1	1.50	9.49E-01	1.00	8.35E-01	1.50
Blastococcus	p__Actinobacteria;c__unidentified_Actinobacteria;o__Fran	4.11E-05	4.11E-05	6.16E-05	4.77E-01	9.78E-01	1	1	0.67	1	0.67	9.66E-01	1.00

	kiales;f__Geodermatophilaceae;g__Blastococcus;												
Sporichthyaceae	p__Actinobacteria;c__unidentified_Actinobacteria;o__Frankiales;f__Sporichthyaceae;	5.14E-05	0.002526	4.11E-05	2.52E-01	4.34E-01	0	1	1.25	NA	61.47	NA	0.02
Kineosporiales	p__Actinobacteria;c__unidentified_Actinobacteria;o__Kineosporiales;	3.08E-05	4.11E-05	6.16E-05	8.30E-01	9.80E-01	4.02E-02	1	0.50	1	0.67	9.99E-01	0.75
Kineosporiaceae	p__Actinobacteria;c__unidentified_Actinobacteria;o__Kineosporiales;f__Kineosporiaceae;	3.08E-05	4.11E-05	6.16E-05	8.31E-01	9.57E-01	1	1	0.50	1	0.67	1	0.75
Micrococcales	p__Actinobacteria;c__unidentified_Actinobacteria;o__Micrococcales;	0.031015	0.0213	0.013875	3.41E-05	1.15E-05	9.13E-04	3.65E-04	2.24	1.32E-01	1.54	3.70E-01	1.46
Brevibacteriaceae	p__Actinobacteria;c__unidentified_Actinobacteria;o__Micrococcales;f__Brevibacteriaceae;	2.05E-05	4.11E-05	4.11E-05	8.31E-01	6.98E-01	1	7.46E-01	0.50	1	1.00	1	0.50
Brevibacterium	p__Actinobacteria;c__unidentified_Actinobacteria;o__Micrococcales;f__Brevibacteriaceae;g__Brevibacterium;	2.05E-05	4.11E-05	4.11E-05	8.24E-01	6.25E-01	1	4.86E-01	0.50	1	1.00	1	0.50
Dermabacteraceae	p__Actinobacteria;c__unidentified_Actinobacteria;o__Micr	4.11E-05	3.08E-05	2.05E-05	2.45E-01	1.67E-01	1	2.17E-01	2.00	1	1.50	7.92E-01	1.33

	ococcales;f__Dermabacteraceae;												
Brachybacterium	p__Actinobacteria;c__unidentified_Actinobacteria;o__Micrococcales;f__Dermabacteraceae;g__Brachybacterium;	4.11E-05	3.08E-05	2.05E-05	2.04E-01	1.23E-01	1	4.48E-02	2.00	1	1.50	9.66E-01	1.33
Dermacoccaceae	p__Actinobacteria;c__unidentified_Actinobacteria;o__Micrococcales;f__Dermacoccaceae;	4.11E-05	2.05E-05	4.11E-05	5.05E-01	7.90E-01	1	1	1.00	1	0.50	9.74E-01	2.00
Kytococcus	p__Actinobacteria;c__unidentified_Actinobacteria;o__Micrococcales;f__Dermacoccaceae;g__Kytococcus;	4.11E-05	2.05E-05	4.11E-05	4.84E-01	7.51E-01	1	9.65E-01	1.00	1	0.50	9.91E-01	2.00
Intrasporangiaceae	p__Actinobacteria;c__unidentified_Actinobacteria;o__Micrococcales;f__Intrasporangiaceae;	6.16E-05	2.05E-05	4.11E-05	9.55E-02	1.49E-01	1	8.06E-01	1.50	1	0.50	1	3.00
Oryzihumus	p__Actinobacteria;c__unidentified_Actinobacteria;o__Micrococcales;f__Intrasporangiaceae;g__Oryzihumus;	4.11E-05	6.16E-05	8.21E-05	8.24E-01	5.88E-01	0	1	0.50	NA	0.75	NA	0.67
Microbacteriaceae	p__Actinobacteria;c__unidentified_Actinobacteria;o__Micrococcales;f__Microbacteriaceae;	6.16E-05	4.11E-05	8.22E-05	5.49E-02	7.11E-01	1	8.51E-01	0.75	2.69E-01	0.50	2.19E-01	1.50

Candidatus Aquiluna	p__Actinobacteria;c__unidentified_Actinobacteria;o__Micrococcales;f__Microbacteriaceae;g__Candidatus_Aquiluna;	2.05E-05	4.11E-05	4.11E-05	7.98E-01	6.24E-01	0	9.24E-01	0.50	1	1.00	9.89E-01	0.50
Leifsonia	p__Actinobacteria;c__unidentified_Actinobacteria;o__Micrococcales;f__Microbacteriaceae;g__Leifsonia;	4.11E-05	5.14E-05	6.16E-05	8.39E-01	8.52E-01	1	1	0.67	1	0.83	9.84E-01	0.80
Microbacterium	p__Actinobacteria;c__unidentified_Actinobacteria;o__Micrococcales;f__Microbacteriaceae;g__Microbacterium;	2.05E-05	4.11E-05	2.05E-05	9.98E-01	9.78E-01	1	9.53E-01	1.00	1	2.00	1	0.50
ML602J-51	p__Actinobacteria;c__unidentified_Actinobacteria;o__Micrococcales;f__Microbacteriaceae;g__ML602J-51;	6.16E-05	4.11E-05	4.11E-05	3.53E-01	2.28E-01	0	6.87E-01	1.50	1	1.00	1	1.50
Micrococcaceae	p__Actinobacteria;c__unidentified_Actinobacteria;o__Micrococcales;f__Micrococcaceae;	0.030728	0.021259	0.013772	2.94E-05	1.19E-05	4.50E-03	7.64E-04	2.23	1.67E-01	1.54	3.04E-01	1.45
Arthrobacter	p__Actinobacteria;c__unidentified_Actinobacteria;o__Micrococcales;f__Micrococcaceae;g__Arthrobacter;	6.16E-05	6.16E-05	4.11E-05	4.42E-01	2.87E-01	1	1	1.50	1	1.50	9.14E-01	1.00
Kocuria	p__Actinobacteria;c__unidentified_Actinobacteria;o__Micr	4.11E-05	2.05E-05	3.08E-05	6.16E-01	4.85E-01	6.50E-01	1	1.33	1	0.67	5.05E-01	2.00

	ococcales;f__Micrococcaceae;g__Kocuria;												
Micrococcus	p__Actinobacteria;c__unidentified_Actinobacteria;o__Micrococcales;f__Micrococcaceae;g__Micrococcus;	4.11E-05	4.11E-05	4.11E-05	6.02E-01	5.23E-01	1	6.12E-01	1.00	1	1.00	1	1.00
Rothia	p__Actinobacteria;c__unidentified_Actinobacteria;o__Micrococcales;f__Micrococcaceae;g__Rothia;	0.030625	0.021218	0.013382	2.98E-05	8.99E-06	2.54E-03	6.28E-05	2.29	8.48E-02	1.59	2.33E-01	1.44
Micromonosporales	p__Actinobacteria;c__unidentified_Actinobacteria;o__Micromonosporales;	4.11E-05	3.08E-05	6.16E-05	7.54E-01	9.95E-01	1	1	0.67	9.62E-01	0.50	1	1.33
Micromonosporaceae	p__Actinobacteria;c__unidentified_Actinobacteria;o__Micromonosporales;f__Micromonosporaceae;	4.11E-05	3.08E-05	6.16E-05	7.62E-01	9.92E-01	1	1	0.67	1	0.50	1	1.33
Propionibacteriales	p__Actinobacteria;c__unidentified_Actinobacteria;o__Propionibacteriales;	0.000267	8.22E-05	8.22E-05	1.90E-05	9.48E-05	1.07E-05	4.07E-03	3.25	1	1.00	1.44E-01	3.25
Nocardioidaceae	p__Actinobacteria;c__unidentified_Actinobacteria;o__Propionibacteriales;f__Nocardioidaceae;	6.16E-05	4.11E-05	4.11E-05	5.45E-01	6.11E-01	1	3.46E-01	1.50	1	1.00	3.62E-01	1.50
Kribbella	p__Actinobacteria;c__unidentified_Actinobacteria;o__Propi	4.11E-05	0	3.08E-05	5.58E-01	6.23E-01	0	1	1.33	NA	NA	NA	NA

	onibacteriales;f__Nocardioidaceae;g__Kribbella;												
Marmoricola	p__Actinobacteria;c__unidentified_Actinobacteria;o__Propionibacteriales;f__Nocardioidaceae;g__Marmoricola;	4.11E-05	2.05E-05	4.11E-05	6.27E-01	9.78E-01	1	1	1.00	1	0.50	1	2.00
Nocardioides	p__Actinobacteria;c__unidentified_Actinobacteria;o__Propionibacteriales;f__Nocardioidaceae;g__Nocardioides;	6.16E-05	4.11E-05	4.11E-05	1.79E-01	1.64E-01	1	5.21E-01	1.50	1	1.00	9.91E-01	1.50
Propionibacteriaceae	p__Actinobacteria;c__unidentified_Actinobacteria;o__Propionibacteriales;f__Propionibacteriaceae;	0.000216	6.16E-05	8.22E-05	1.26E-06	1.19E-05	1.45E-02	1.40E-02	2.62	1	0.75	2.38E-01	3.50
Propionibacterium	p__Actinobacteria;c__unidentified_Actinobacteria;o__Propionibacteriales;f__Propionibacteriaceae;g__Propionibacterium;	6.16E-05	4.11E-05	6.16E-05	1.72E-01	1.06E-01	3.25E-01	2.32E-01	1.00	1	0.67	5.75E-01	1.50
Pseudonocardiales	p__Actinobacteria;c__unidentified_Actinobacteria;o__Pseudonocardiales;	4.11E-05	3.08E-05	2.05E-05	3.24E-01	2.55E-01	7.80E-02	3.14E-01	2.00	1	1.50	1	1.33
Pseudonocardiaceae	p__Actinobacteria;c__unidentified_Actinobacteria;o__Pseudonocardiales;f__Pseudonocardiaceae;	4.11E-05	3.08E-05	2.05E-05	3.31E-01	2.57E-01	3.10E-01	2.32E-01	2.00	1	1.50	1	1.33

Pseudonocardia	p__Actinobacteria;c__unidentified_Actinobacteria;o__Pseudonocardiales;f__Pseudonocardiaceae;g__Pseudonocardia;	4.11E-05	6.16E-05	2.05E-05	3.07E-01	2.39E-01	0	1	2.00	1	3.00	1	0.67
Streptomycetales	p__Actinobacteria;c__unidentified_Actinobacteria;o__Streptomycetales;	4.11E-05	4.11E-05	4.11E-05	9.60E-01	9.61E-01	1	1	1.00	6.69E-01	1.00	1	1.00
Streptomycetaceae	p__Actinobacteria;c__unidentified_Actinobacteria;o__Streptomycetales;f__Streptomycetaceae;	4.11E-05	4.11E-05	4.11E-05	9.68E-01	9.18E-01	1	1	1.00	6.84E-01	1.00	1	1.00
Streptomyces	p__Actinobacteria;c__unidentified_Actinobacteria;o__Streptomycetales;f__Streptomycetaceae;g__Streptomyces;	4.11E-05	4.11E-05	4.11E-05	9.61E-01	9.29E-01	1	1	1.00	6.01E-01	1.00	1	1.00
Streptosporangiales	p__Actinobacteria;c__unidentified_Actinobacteria;o__Streptosporangiales;	4.11E-05	2.05E-05	7.19E-05	2.56E-01	9.80E-01	0	1	0.57	NA	0.29	NA	2.00
Armatimonadetes	p__Armatimonadetes;	6.16E-05	4.11E-05	4.11E-05	3.54E-01	4.26E-01	1	9.75E-01	1.50	1	1.00	1	1.50
Bacteroidetes	p__Bacteroidetes;	0.05897	0.077045	0.032689	4.61E-03	3.87E-02	1.31E-01	8.48E-02	1.80	7.01E-02	2.36	7.85E-01	0.77
Bacteroidia	p__Bacteroidetes;c__Bacteroidia;	0.053178	0.06125	0.022101	5.97E-04	4.16E-03	1.19E-03	1.89E-02	2.41	8.60E-02	2.77	1	0.87
Bacteroidales	p__Bacteroidetes;c__Bacteroidia;o__Bacteroidales;	0.053178	0.06125	0.022101	6.11E-04	5.10E-03	1.68E-03	2.05E-02	2.41	7.49E-02	2.77	8.82E-01	0.87

Bacteroidaceae	p__Bacteroidetes;c__Bacteroidia;o__Bacteroidales;f__Bacteroidaceae;	0.000246	0.000288	0.000144	3.43E-04	6.18E-02	3.30E-10	1.59E-01	1.71	4.16E-02	2.00	8.68E-01	0.86
Bacteroides	p__Bacteroidetes;c__Bacteroidia;o__Bacteroidales;f__Bacteroidaceae;g__Bacteroides;	0.000246	0.000288	0.000144	3.48E-04	4.97E-02	7.87E-11	1.21E-01	1.71	2.27E-02	2.00	9.59E-01	0.86
Bacteroidales BS11 gut group	p__Bacteroidetes;c__Bacteroidia;o__Bacteroidales;f__Bacteroidales_BS11_gut_group;	0.000246	2.05E-05	4.11E-05	1.79E-02	1.23E-02	0	7.28E-01	6.00	NA	0.50	NA	12.02
Bacteroidales RF16 group	p__Bacteroidetes;c__Bacteroidia;o__Bacteroidales;f__Bacteroidales_RF16_group;	7.19E-05	4.11E-05	6.16E-05	9.75E-01	9.72E-01	0	NA	1.17	NA	0.67	NA	1.75
Bacteroidales S24-7 group	p__Bacteroidetes;c__Bacteroidia;o__Bacteroidales;f__Bacteroidales_S24-7_group;	8.22E-05	6.16E-05	0.000103	8.31E-01	9.18E-01	4.32E-07	1	0.80	7.76E-01	0.60	8.68E-01	1.33
Porphyromonadaceae	p__Bacteroidetes;c__Bacteroidia;o__Bacteroidales;f__Porphyromonadaceae;	0.012324	0.007107	0.006244	6.48E-04	2.07E-04	5.09E-01	1.40E-02	1.97	1	1.14	6.58E-01	1.73
Odoribacter	p__Bacteroidetes;c__Bacteroidia;o__Bacteroidales;f__Porphyromonadaceae;g__Odoribacter;	4.11E-05	0	2.05E-05	1.59E-01	2.25E-01	0	2.39E-01	2.00	NA	NA	NA	NA
Parabacteroides	p__Bacteroidetes;c__Bacteroidia;o__Bacteroidales;f__Porphyromonadaceae;g__Parabacteroides;	4.11E-05	2.05E-05	4.11E-05	3.23E-01	8.86E-01	1	1	1.00	1	0.50	4.01E-01	2.00

Porphyromonas	p__Bacteroidetes;c__Bacteroidia;o__Bacteroidales;f__Porphyromonadaceae;g__Porphyromonas;	0.012036	0.007004	0.005833	1.05E-03	3.71E-04	5.44E-01	2.99E-02	2.06	1	1.20	6.92E-01	1.72
Tannerella	p__Bacteroidetes;c__Bacteroidia;o__Bacteroidales;f__Porphyromonadaceae;g__Tannerella;	0.000298	0.000123	0.000144	5.31E-06	1.16E-03	1.34E-01	8.07E-02	2.07	1	0.86	1.14E-01	2.42
unidentified Porphyromonadaceae	p__Bacteroidetes;c__Bacteroidia;o__Bacteroidales;f__Porphyromonadaceae;g__unidentified_Porphyromonadaceae;	0.000134	4.11E-05	8.22E-05	8.86E-05	6.30E-03	1.91E-02	3.61E-02	1.62	1	0.50	2.53E-02	3.25
Prevotellaceae	p__Bacteroidetes;c__Bacteroidia;o__Bacteroidales;f__Prevotellaceae;	0.029331	0.044017	0.013988	8.20E-05	7.85E-03	2.86E-04	1.40E-02	2.10	4.50E-02	3.15	3.19E-01	0.67
Alloprevotella	p__Bacteroidetes;c__Bacteroidia;o__Bacteroidales;f__Prevotellaceae;g__Alloprevotella;	0.002485	0.001992	0.001356	1.59E-01	8.60E-02	1	3.29E-01	1.83	7.68E-01	1.47	1	1.25
Prevotella	p__Bacteroidetes;c__Bacteroidia;o__Bacteroidales;f__Prevotellaceae;g__Prevotella;	0.005874	0.010496	0.003143	2.18E-06	9.06E-03	1.52E-07	3.18E-01	1.87	3.70E-03	3.34	1.43E-01	0.56
Prevotella 1	p__Bacteroidetes;c__Bacteroidia;o__Bacteroidales;f__Prevotellaceae;g__Prevotella_1;	0.000113	4.11E-05	2.05E-05	1.28E-01	6.07E-02	0	5.60E-01	5.51	NA	2.00	NA	2.75

Prevotella 2	p__Bacteroidetes;c__Bacteroidia;o__Bacteroidales;f__Prevotellaceae;g__Prevotella_2;	0.00037	0.000401	0.000277	1.58E-01	4.67E-01	2.56E-02	9.65E-01	1.33	3.62E-01	1.44	1	0.92
Prevotella 6	p__Bacteroidetes;c__Bacteroidia;o__Bacteroidales;f__Prevotellaceae;g__Prevotella_6;	0.001232	0.001222	0.00076	1.51E-01	4.40E-01	4.40E-01	2.22E-01	1.62	4.78E-01	1.61	9.91E-01	1.01
Prevotella 7	p__Bacteroidetes;c__Bacteroidia;o__Bacteroidales;f__Prevotellaceae;g__Prevotella_7;	0.014111	0.020622	0.005833	4.93E-05	1.21E-02	3.42E-05	2.88E-02	2.42	3.79E-01	3.54	8.63E-01	0.68
Prevotella 9	p__Bacteroidetes;c__Bacteroidia;o__Bacteroidales;f__Prevotellaceae;g__Prevotella_9;	4.11E-05	5.14E-05	4.11E-05	3.12E-01	2.49E-01	1.56E-01	7.51E-01	1.00	1	1.25	1.86E-01	0.80
Prevotellaceae UCG-003	p__Bacteroidetes;c__Bacteroidia;o__Bacteroidales;f__Prevotellaceae;g__Prevotellaceae_UCG-003;	0.000123	0	2.05E-05	7.26E-02	9.95E-02	0	5.31E-01	6.01	NA	NA	NA	NA
Prevotellaceae UCG-004	p__Bacteroidetes;c__Bacteroidia;o__Bacteroidales;f__Prevotellaceae;g__Prevotellaceae_UCG-004;	6.16E-05	2.05E-05	3.08E-05	3.24E-03	2.87E-03	0	2.32E-02	2.00	1	0.67	4.56E-01	3.00
unidentified Prevotellaceae	p__Bacteroidetes;c__Bacteroidia;o__Bacteroidales;f__Prevotellaceae;g__unidentified_Prevotellaceae;	4.11E-05	2.05E-05	4.11E-05	3.62E-01	9.93E-01	1	1	1.00	3.62E-01	0.50	4.01E-01	2.00

Rikenellaceae	p__Bacteroidetes;c__Bacteroidia;o__Bacteroidales;f__Rikenellaceae;	0.000123	4.11E-05	0.000103	4.14E-06	2.53E-01	1.80E-02	1	1.20	2.57E-01	0.40	1.97E-01	3.00
Alistipes	p__Bacteroidetes;c__Bacteroidia;o__Bacteroidales;f__Rikenellaceae;g__Alistipes;	5.14E-05	2.05E-05	6.16E-05	1.10E-02	5.15E-01	1	1	0.83	6.48E-01	0.33	1.39E-01	2.50
Rikenellaceae RC9 gut group	p__Bacteroidetes;c__Bacteroidia;o__Bacteroidales;f__Rikenellaceae;g__Rikenellaceae_RC9_gut_group;	0.000113	4.11E-05	6.16E-05	1.92E-03	1.06E-01	1.07E-03	1.76E-01	1.83	9.96E-01	0.67	7.40E-01	2.75
unidentified Bacteroidales	p__Bacteroidetes;c__Bacteroidia;o__Bacteroidales;f__unidentified_Bacteroidales;	0.000123	3.08E-05	0.000103	9.90E-03	8.99E-01	4.32E-04	1	1.20	7.76E-01	0.30	6.19E-01	4.00
Phocaeicola	p__Bacteroidetes;c__Bacteroidia;o__Bacteroidales;f__unidentified_Bacteroidales;g__Phocaeicola;	0.000123	3.08E-05	0.000103	9.32E-03	8.91E-01	6.35E-04	1	1.20	7.69E-01	0.30	7.41E-01	4.00
Cytophagia	p__Bacteroidetes;c__Cytophagia;	0.000123	4.11E-05	5.14E-05	3.06E-02	7.51E-02	3.12E-04	7.76E-02	2.40	1	0.80	2.47E-01	3.00
Cytophagales	p__Bacteroidetes;c__Cytophagia;o__Cytophagales;	0.000103	4.11E-05	4.11E-05	4.19E-02	7.84E-02	1.71E-04	1.96E-01	2.50	1	1.00	6.41E-01	2.50
Cytophagaceae	p__Bacteroidetes;c__Cytophagia;o__Cytophagales;f__Cytophagaceae;	0.000103	4.11E-05	4.11E-05	4.39E-02	7.30E-02	5.61E-04	8.12E-02	2.50	1	1.00	3.46E-01	2.50

Fibrella	p__Bacteroidetes;c__Cytophagia;o__Cytophagales;f__Cytophagaceae;g__Fibrella;	6.16E-05	4.11E-05	4.11E-05	7.79E-01	5.41E-01	0	8.02E-01	1.50	1	1.00	9.59E-01	1.50
Flavobacteriia	p__Bacteroidetes;c__Flavobacteriia;	0.003369	0.004046	0.003666	5.39E-01	5.68E-01	1	9.36E-01	0.92	1	1.10	9.24E-01	0.83
Flavobacteriales	p__Bacteroidetes;c__Flavobacteriia;o__Flavobacteriales;	0.003369	0.004046	0.003666	4.77E-01	5.44E-01	1	9.29E-01	0.92	1	1.10	8.82E-01	0.83
Flavobacteriaceae	p__Bacteroidetes;c__Flavobacteriia;o__Flavobacteriales;f__Flavobacteriaceae;	0.003369	0.003985	0.003646	4.72E-01	5.15E-01	1	1	0.92	1	1.09	8.68E-01	0.85
Bergeyella	p__Bacteroidetes;c__Flavobacteriia;o__Flavobacteriales;f__Flavobacteriaceae;g__Bergeyella;	0.000411	0.000277	0.000298	2.39E-02	1.05E-01	1	2.02E-01	1.38	7.69E-01	0.93	2.73E-02	1.48
Capnocytophaga	p__Bacteroidetes;c__Flavobacteriia;o__Flavobacteriales;f__Flavobacteriaceae;g__Capnocytophaga;	0.001951	0.002177	0.001273	1.52E-02	6.07E-02	1	6.92E-02	1.53	1	1.71	1	0.90
Chryseobacterium	p__Bacteroidetes;c__Flavobacteriia;o__Flavobacteriales;f__Flavobacteriaceae;g__Chryseobacterium;	0.000226	0.000144	0.000688	3.14E-04	1.46E-03	2.30E-01	7.24E-02	0.33	2.65E-01	0.21	1	1.57
Flavobacterium	p__Bacteroidetes;c__Flavobacteriia;o__Flavobacteriales;f__Flavobacteriaceae;g__Flavobacterium;	7.19E-05	4.11E-05	0.000411	1.98E-01	1.06E-01	1.63E-03	5.21E-01	0.18	1	0.10	1	1.75

Sphingobacteriia	p__Bacteroidetes;c__Sphingobacteriia;	0.000185	0.000195	0.000185	6.00E-01	8.34E-01	1	9.22E-01	1.00	1	1.06	1	0.95
Sphingobacteriales	p__Bacteroidetes;c__Sphingobacteriia;o__Sphingobacteriales;	0.000185	0.000195	0.000185	5.57E-01	8.59E-01	1	8.71E-01	1.00	1	1.06	8.71E-01	0.95
Chitinophagaceae	p__Bacteroidetes;c__Sphingobacteriia;o__Sphingobacteriales;f__Chitinophagaceae;	0.000103	4.11E-05	4.11E-05	2.28E-03	2.51E-03	1.68E-01	2.86E-02	2.50	1	1.00	1	2.50
Flavisolibacter	p__Bacteroidetes;c__Sphingobacteriia;o__Sphingobacteriales;f__Chitinophagaceae;g__Flavisolibacter;	5.14E-05	2.05E-05	4.11E-05	4.40E-01	9.40E-01	0	1	1.25	NA	0.50	NA	2.50
Terrimonas	p__Bacteroidetes;c__Sphingobacteriia;o__Sphingobacteriales;f__Chitinophagaceae;g__Terrimonas;	8.22E-05	4.11E-05	6.16E-05	1.51E-01	1.96E-01	1	1	1.33	1	0.67	9.89E-01	2.00
Saprospiraceae	p__Bacteroidetes;c__Sphingobacteriia;o__Sphingobacteriales;f__Saprospiraceae;	2.05E-05	0.002403	3.08E-05	2.84E-01	8.97E-01	0	1	0.67	NA	78.02	NA	0.01
Sphingobacteriaceae	p__Bacteroidetes;c__Sphingobacteriia;o__Sphingobacteriales;f__Sphingobacteriaceae;	8.22E-05	0.000205	0.000144	1.74E-01	8.65E-02	1	4.85E-01	0.57	1	1.43	8.55E-01	0.40
Nubsella	p__Bacteroidetes;c__Sphingobacteriia;o__Sphingobacteriales;f__Sphingobacteriaceae;g__Nubsella;	2.05E-05	2.05E-05	0.000154	4.35E-02	3.63E-02	9.32E-02	1	0.13	1	0.13	1	1.00

Sphingobacterium	p__Bacteroidetes;c__Sphingobacteriia;o__Sphingobacteriales;f__Sphingobacteriaceae;g__Sphingobacterium;	6.16E-05	0.000205	0.000154	2.37E-02	1.15E-02	1	5.32E-01	0.40	1	1.33	9.59E-01	0.30
WCHB1-69	p__Bacteroidetes;c__Sphingobacteriia;o__Sphingobacteriales;f__WCHB1-69;	4.11E-05	4.11E-05	6.16E-05	1.01E-01	8.19E-02	6.46E-01	1.45E-01	0.67	1	0.67	9.55E-01	1.00
unidentified WCHB1-69	p__Bacteroidetes;c__Sphingobacteriia;o__Sphingobacteriales;f__WCHB1-69;g__unidentified_WCHB1-69;	4.11E-05	4.11E-05	6.16E-05	1.04E-01	6.79E-02	1	8.07E-02	0.67	1	0.67	9.91E-01	1.00
Candidate division SR1	p__Candidate_division_SR1;	0.00154	0.002362	0.00151	2.57E-01	5.25E-01	1	8.81E-01	1.02	1	1.56	6.88E-02	0.65
unidentified Candidate division SR1	p__Candidate_division_SR1;c__unidentified_Candidate_division_SR1;	0.00154	0.002362	0.00151	2.97E-01	6.04E-01	1	9.22E-01	1.02	1	1.56	1.13E-01	0.65
unidentified Candidate division SR1	p__Candidate_division_SR1;c__unidentified_Candidate_division_SR1;o__unidentified_Candidate_division_SR1;	0.00154	0.002362	0.00151	2.76E-01	6.43E-01	1	9.29E-01	1.02	1	1.56	1.24E-01	0.65
unidentified Candidate division SR1	p__Candidate_division_SR1;c__unidentified_Candidate_division_SR1;o__unidentified_Candidate_division_SR1;f__u	0.00154	0.002362	0.00151	2.68E-01	6.46E-01	1	1	1.02	1	1.56	1.18E-01	0.65

	nidentified_Candidate_division_SR1;												
unidentified Candidate division SR1	p__Candidate_division_SR1;c__unidentified_Candidate_division_SR1;o__unidentified_Candidate_division_SR1;f__unidentified_Candidate_division_SR1;g__unidentified_Candidate_division_SR1;	0.00154	0.002362	0.00151	2.55E-01	5.72E-01	1	9.53E-01	1.02	1	1.56	1.15E-01	0.65
Chlorobi	p__Chlorobi;	4.11E-05	0.000298	4.11E-05	8.05E-01	6.22E-01	0	9.75E-01	1.00	3.35E-01	7.25	1	0.14
Chlorobia	p__Chlorobi;c__Chlorobia;	4.11E-05	0.000277	4.11E-05	9.09E-01	9.80E-01	0	1	1.00	1	6.75	1	0.15
Chlorobiales	p__Chlorobi;c__Chlorobia;o__Chlorobiales;	4.11E-05	0.000277	4.11E-05	9.37E-01	9.91E-01	0	1	1.00	1	6.75	1	0.15
Ignavibacteria	p__Chlorobi;c__Ignavibacteria;	2.05E-05	4.11E-05	0.000113	6.00E-01	5.00E-01	0	1	0.18	NA	0.36	NA	0.50
Ignavibacteriales	p__Chlorobi;c__Ignavibacteria;o__Ignavibacteriales;	2.05E-05	4.11E-05	0.000113	5.85E-01	4.63E-01	0	1	0.18	NA	0.36	NA	0.50
Chloroflexi	p__Chloroflexi;	8.22E-05	4.11E-05	4.11E-05	2.57E-01	4.26E-01	1.48E-03	8.81E-01	2.00	1	1.00	9.31E-02	2.00
Anaerolineae	p__Chloroflexi;c__Anaerolineae;	4.11E-05	2.05E-05	2.05E-05	6.00E-01	6.04E-01	1	1	2.00	1	1.00	9.24E-01	2.00
Anaerolineales	p__Chloroflexi;c__Anaerolineae;o__Anaerolineales;	4.11E-05	2.05E-05	2.05E-05	5.85E-01	6.28E-01	1	1	2.00	1	1.00	8.71E-01	2.00
Anaerolineaceae	p__Chloroflexi;c__Anaerolineae;o__Anaerolineales;f__Anaerolineaceae;	4.11E-05	2.05E-05	2.05E-05	5.90E-01	6.18E-01	1	1	2.00	1	1.00	8.35E-01	2.00

Chloroflexia	p__Chloroflexi;c__Chloroflexia;	9.24E-05	0	4.11E-05	6.00E-01	6.25E-01	0	9.96E-01	2.25	NA	NA	NA	NA
Chloroflexales	p__Chloroflexi;c__Chloroflexia;o__Chloroflexales;	8.22E-05	0	4.11E-05	6.43E-01	7.61E-01	0	1	2.00	NA	NA	NA	NA
Roseiflexaceae	p__Chloroflexi;c__Chloroflexia;o__Chloroflexales;f__Roseiflexaceae;	8.22E-05	0	4.11E-05	6.54E-01	7.37E-01	0	1	2.00	NA	NA	NA	NA
Roseiflexus	p__Chloroflexi;c__Chloroflexia;o__Chloroflexales;f__Roseiflexaceae;g__Roseiflexus;	8.22E-05	0	4.11E-05	6.53E-01	7.00E-01	0	1	2.00	NA	NA	NA	NA
JG37-AG-4	p__Chloroflexi;c__JG37-AG-4;	4.11E-05	3.08E-05	4.11E-05	2.79E-01	4.40E-01	1	9.95E-01	1.00	1	0.75	1	1.33
KD4-96	p__Chloroflexi;c__KD4-96;	5.14E-05	4.11E-05	5.14E-05	6.00E-01	9.80E-01	1	1	1.00	1	0.80	1	1.25
Ktedonobacteria	p__Chloroflexi;c__Ktedonobacteria;	4.11E-05	3.08E-05	0.000103	5.60E-01	8.83E-01	1	1	0.40	1	0.30	1	1.33
JG30-KF-AS9	p__Chloroflexi;c__Ktedonobacteria;o__JG30-KF-AS9;	6.16E-05	2.05E-05	9.24E-05	5.40E-01	8.92E-01	0	1	0.67	NA	0.22	NA	3.00
Ktedonobacterales	p__Chloroflexi;c__Ktedonobacteria;o__Ktedonobacterales;	2.05E-05	6.17E-05	2.05E-05	8.13E-01	8.32E-01	2.58E-01	1	1.00	7.43E-01	3.01	1	0.33
S085	p__Chloroflexi;c__S085;	4.11E-05	2.05E-05	9.24E-05	2.33E-01	2.78E-01	0	9.96E-01	0.44	NA	0.22	NA	2.00
TK10	p__Chloroflexi;c__TK10;	4.11E-05	3.08E-05	3.08E-05	6.00E-01	5.77E-01	1	1	1.33	9.92E-01	1.00	1	1.33
Cyanobacteria	p__Cyanobacteria;	0.000123	6.16E-05	6.16E-05	1.25E-01	1.22E-01	3.73E-01	1.15E-02	2.00	1	1.00	9.31E-02	2.00
Chloroplast	p__Cyanobacteria;c__Chloroplast;	8.22E-05	5.14E-05	6.16E-05	3.35E-01	2.46E-01	4.51E-01	2.33E-01	1.33	1	0.83	9.84E-01	1.60

unidentified Chloroplast	p__Cyanobacteria;c__Chloroplast;o__unidentified_Chloroplast;	8.22E-05	5.14E-05	6.16E-05	3.24E-01	2.66E-01	4.12E-01	2.41E-01	1.33	1	0.83	7.91E-01	1.60
unidentified Chloroplast	p__Cyanobacteria;c__Chloroplast;o__unidentified_Chloroplast;f__unidentified_Chloroplast;	8.22E-05	5.14E-05	6.16E-05	3.30E-01	2.80E-01	1	2.42E-01	1.33	1	0.83	7.06E-01	1.60
unidentified Chloroplast	p__Cyanobacteria;c__Chloroplast;o__unidentified_Chloroplast;f__unidentified_Chloroplast;g__unidentified_Chloroplast;	8.22E-05	5.14E-05	6.16E-05	3.14E-01	2.41E-01	1	2.42E-01	1.33	1	0.83	8.63E-01	1.60
Melainabacteria	p__Cyanobacteria;c__Melainabacteria;	4.11E-05	4.11E-05	4.11E-05	6.29E-01	6.14E-01	1	1	1.00	1	1.00	1	1.00
Obscuribacterales	p__Cyanobacteria;c__Melainabacteria;o__Obscuribacterales;	4.11E-05	5.14E-05	3.08E-05	4.29E-01	9.80E-01	1	1	1.33	1	1.67	7.91E-01	0.80
unidentified Cyanobacteria	p__Cyanobacteria;c__unidentified_Cyanobacteria;	0.000246	6.16E-05	0.000185	6.29E-01	9.80E-01	0	1	1.33	NA	0.33	NA	4.00
Deinococcus-Thermus	p__Deinococcus-Thermus;	4.11E-05	6.16E-05	4.11E-05	3.60E-01	5.25E-01	1	1.00E+00	1.00	1	1.50	7.27E-01	0.67
Deinococci	p__Deinococcus-Thermus;c__Deinococci;	4.11E-05	6.16E-05	4.11E-05	4.27E-01	6.04E-01	1	1	1.00	1	1.50	9.24E-01	0.67
Deinococcales	p__Deinococcus-Thermus;c__Deinococci;o__Deinococcales;	4.11E-05	6.16E-05	4.11E-05	3.18E-01	7.99E-01	1	1	1.00	9.60E-01	1.50	9.48E-01	0.67

Deinococcaceae	p__Deinococcus-Thermus;c__Deinococci;o__Deinococcales;f__Deinococcaceae;	4.11E-05	6.16E-05	2.05E-05	1.87E-01	8.67E-01	1	1.56E-01	2.00	1	3.00	9.74E-01	0.67
Deinococcus	p__Deinococcus-Thermus;c__Deinococci;o__Deinococcales;f__Deinococcaceae;g__Deinococcus;	4.11E-05	6.16E-05	2.05E-05	1.73E-01	8.56E-01	1	1.76E-01	2.00	1	3.00	9.91E-01	0.67
Fibrobacteres	p__Fibrobacteres;	0.000329	0	4.11E-05	8.77E-03	1.21E-02	0	1.46E-01	8.00	NA	NA	NA	NA
Fibrobacteria	p__Fibrobacteres;c__Fibrobacteria;	0.000329	0	4.11E-05	1.03E-02	1.37E-02	0	4.68E-01	8.00	NA	NA	NA	NA
Fibrobacterales	p__Fibrobacteres;c__Fibrobacteria;o__Fibrobacterales;	0.000329	0	4.11E-05	3.64E-02	5.70E-02	0	7.12E-01	8.00	NA	NA	NA	NA
Firmicutes	p__Firmicutes;	0.391098	0.314423	0.240521	1.41E-05	3.54E-06	1.75E-02	1.58E-03	1.63	7.01E-02	1.31	5.56E-01	1.24
Bacilli	p__Firmicutes;c__Bacilli;	0.187159	0.13431	0.133108	2.29E-02	9.43E-03	1	2.52E-01	1.41	1	1.01	5.49E-01	1.39
Bacillales	p__Firmicutes;c__Bacilli;o__Bacillales;	0.01561	0.009982	0.00876	6.59E-06	2.33E-06	4.60E-06	4.20E-04	1.78	7.43E-01	1.14	3.08E-01	1.56
Alicyclobacillaceae	p__Firmicutes;c__Bacilli;o__Bacillales;f__Alicyclobacillaceae;	4.11E-05	4.11E-05	5.14E-05	2.56E-01	3.73E-01	9.11E-01	1	0.80	1	0.80	1	1.00
Bacillaceae	p__Firmicutes;c__Bacilli;o__Bacillales;f__Bacillaceae;	0.003985	0.000657	0.000955	3.93E-08	1.19E-05	6.39E-10	5.76E-08	4.17	7.76E-01	0.69	2.38E-06	6.06
Anoxybacillus	p__Firmicutes;c__Bacilli;o__Bacillales;f__Bacillaceae;g__Anoxybacillus;	0.003451	0.000575	0.000811	7.42E-08	2.53E-05	1.39E-08	1.27E-04	4.25	5.54E-01	0.71	4.74E-05	6.00

Bacillus	p__Firmicutes;c__Bacilli;o__Bacillales;f__Bacillaceae;g__Bacillus;	0.000103	6.16E-05	6.16E-05	1.14E-01	5.51E-02	1	2.38E-01	1.67	1	1.00	1	1.67
Geobacillus	p__Firmicutes;c__Bacilli;o__Bacillales;f__Bacillaceae;g__Geobacillus;	0.000164	4.11E-05	6.16E-05	5.60E-08	3.20E-05	1.54E-06	4.48E-02	2.67	7.29E-01	0.67	8.04E-03	4.00
Family XI	p__Firmicutes;c__Bacilli;o__Bacillales;f__Family_XI;	0.007743	0.007168	0.005494	5.49E-02	3.17E-02	1	2.85E-01	1.41	8.43E-01	1.30	1	1.08
Gemella	p__Firmicutes;c__Bacilli;o__Bacillales;f__Family_XI;g__Gemella;	0.007743	0.007168	0.005494	5.17E-02	2.75E-02	1	2.32E-01	1.41	7.68E-01	1.30	1	1.08
Family XII	p__Firmicutes;c__Bacilli;o__Bacillales;f__Family_XII;	4.11E-05	2.05E-05	6.16E-05	4.31E-02	7.07E-01	1	9.35E-01	0.67	9.49E-01	0.33	6.58E-01	2.00
Exiguobacterium	p__Firmicutes;c__Bacilli;o__Bacillales;f__Family_XII;g__Exiguobacterium;	4.11E-05	2.05E-05	6.16E-05	3.82E-02	6.58E-01	1	7.88E-01	0.67	9.47E-01	0.33	7.41E-01	2.00
Paenibacillaceae	p__Firmicutes;c__Bacilli;o__Bacillales;f__Paenibacillaceae;	0.001232	0.000164	0.000308	3.93E-08	1.95E-05	1.57E-11	1.62E-08	4.00	2.69E-01	0.53	1.75E-05	7.50
Brevibacillus	p__Firmicutes;c__Bacilli;o__Bacillales;f__Paenibacillaceae;g__Brevibacillus;	0.000452	8.22E-05	0.000103	5.87E-08	7.88E-06	1.13E-06	1.81E-13	4.40	9.96E-01	0.80	1.73E-09	5.50
Paenibacillus	p__Firmicutes;c__Bacilli;o__Bacillales;f__Paenibacillaceae;g__Paenibacillus;	0.000698	8.22E-05	0.000246	6.32E-08	3.67E-04	8.65E-12	1.66E-04	2.83	6.94E-03	0.33	2.88E-04	8.50

Planococcaceae	p__Firmicutes;c__Bacilli;o__Bacillales;f__Planococcaceae;	9.24E-05	0.000277	6.16E-05	3.28E-01	2.80E-01	1	1	1.50	1	4.50	1	0.33
Chryseomicrobium	p__Firmicutes;c__Bacilli;o__Bacillales;f__Planococcaceae;g__Chryseomicrobium;	4.11E-05	4.11E-05	4.11E-05	9.61E-01	9.81E-01	1	1	1.00	1	1.00	1	1.00
Kurthia	p__Firmicutes;c__Bacilli;o__Bacillales;f__Planococcaceae;g__Kurthia;	9.24E-05	0.000616	4.11E-05	3.13E-01	2.67E-01	2.58E-01	1	2.25	1	14.99	1	0.15
Sporosarcina	p__Firmicutes;c__Bacilli;o__Bacillales;f__Planococcaceae;g__Sporosarcina;	4.11E-05	2.05E-05	4.11E-05	3.39E-01	4.55E-01	1	1	1.00	7.68E-01	0.50	1	2.00
Sporolactobacillaceae	p__Firmicutes;c__Bacilli;o__Bacillales;f__Sporolactobacillaceae;	8.22E-05	4.11E-05	6.16E-05	1.31E-01	9.83E-01	3.02E-01	8.06E-01	1.33	1	0.67	1	2.00
Sporolactobacillus	p__Firmicutes;c__Bacilli;o__Bacillales;f__Sporolactobacillaceae;g__Sporolactobacillus;	4.11E-05	4.11E-05	7.19E-05	1.59E-01	4.66E-01	3.03E-01	9.61E-01	0.57	1	0.57	1	1.00
Staphylococcaceae	p__Firmicutes;c__Bacilli;o__Bacillales;f__Staphylococcaceae;	0.000164	0.000103	0.000103	2.68E-02	1.49E-02	1	5.92E-02	1.60	1	1.00	9.74E-01	1.60
Staphylococcus	p__Firmicutes;c__Bacilli;o__Bacillales;f__Staphylococcaceae;g__Staphylococcus;	0.000144	0.000103	8.22E-05	2.91E-02	1.47E-02	1	4.78E-02	1.75	1	1.25	1	1.40
Lactobacillales	p__Firmicutes;c__Bacilli;o__Lactobacillales;	0.163291	0.111572	0.119357	3.66E-02	2.51E-02	1	3.64E-01	1.37	1	0.93	3.87E-01	1.46

Aerococcaceae	p__Firmicutes;c__Bacilli;o__Lactobacillales;f__Aerococcaceae;	0.001397	0.001766	0.000883	3.90E-03	9.14E-02	9.64E-01	2.92E-01	1.58	5.29E-01	2.00	7.92E-01	0.79
Abiotrophia	p__Firmicutes;c__Bacilli;o__Lactobacillales;f__Aerococcaceae;g__Abiotrophia;	0.001397	0.001766	0.000883	3.65E-03	7.14E-02	9.40E-01	3.40E-01	1.58	5.16E-01	2.00	8.45E-01	0.79
Carnobacteriaceae	p__Firmicutes;c__Bacilli;o__Lactobacillales;f__Carnobacteriaceae;	0.017315	0.011626	0.011102	3.46E-04	1.57E-04	5.43E-01	1.76E-02	1.56	1	1.05	5.60E-01	1.49
Atopostipes	p__Firmicutes;c__Bacilli;o__Lactobacillales;f__Carnobacteriaceae;g__Atopostipes;	4.11E-05	2.05E-05	2.05E-05	9.61E-01	8.69E-01	1	2.38E-01	2.00	1	1.00	1	2.00
Granulicatella	p__Firmicutes;c__Bacilli;o__Lactobacillales;f__Carnobacteriaceae;g__Granulicatella;	0.017274	0.011626	0.01105	3.61E-04	1.98E-04	3.03E-01	3.28E-02	1.56	1	1.05	6.23E-01	1.49
Enterococcaceae	p__Firmicutes;c__Bacilli;o__Lactobacillales;f__Enterococcaceae;	4.11E-05	2.05E-05	6.16E-05	1.06E-01	8.31E-01	4.07E-01	5.62E-01	0.67	1	0.33	1	2.00
Enterococcus	p__Firmicutes;c__Bacilli;o__Lactobacillales;f__Enterococcaceae;g__Enterococcus;	4.11E-05	2.05E-05	6.16E-05	1.02E-01	8.02E-01	7.27E-01	5.31E-01	0.67	1	0.33	1	2.00
Lactobacillaceae	p__Firmicutes;c__Bacilli;o__Lactobacillales;f__Lactobacillaceae;	0.000226	0.000123	0.000195	3.78E-02	6.64E-01	1	1	1.16	1	0.63	1.98E-01	1.83

Lactobacillus	p__Firmicutes;c__Bacilli;o__Lactobacillales;f__Lactobacillaceae;g__Lactobacillus;	0.000205	0.000123	0.000195	3.62E-02	5.94E-01	1	8.89E-01	1.05	1	0.63	1.39E-01	1.67
Leuconostocaceae	p__Firmicutes;c__Bacilli;o__Lactobacillales;f__Leuconostocaceae;	0.000329	3.08E-05	0.001684	1.36E-02	2.59E-01	9.16E-10	1	0.20	1	0.02	4.06E-01	10.67
Leuconostoc	p__Firmicutes;c__Bacilli;o__Lactobacillales;f__Leuconostocaceae;g__Leuconostoc;	0.00951	2.05E-05	0.000185	5.22E-02	4.40E-01	0	1.70E-02	51.44	1	0.11	9.84E-01	463.90
Weissella	p__Firmicutes;c__Bacilli;o__Lactobacillales;f__Leuconostocaceae;g__Weissella;	0.00037	4.11E-05	0.001037	8.66E-02	1.69E-01	4.71E-07	3.87E-01	0.36	6.01E-01	0.04	7.41E-01	9.00
P5D1-392	p__Firmicutes;c__Bacilli;o__Lactobacillales;f__P5D1-392;	0.001869	0.002465	0.002331	3.07E-01	4.18E-01	1	1	0.80	1	1.06	1	0.76
Streptococcaceae	p__Firmicutes;c__Bacilli;o__Lactobacillales;f__Streptococcaceae;	0.139876	0.084028	0.087407	5.62E-02	3.17E-02	1	2.39E-01	1.60	1	0.96	3.62E-01	1.66
Lactococcus	p__Firmicutes;c__Bacilli;o__Lactobacillales;f__Streptococcaceae;g__Lactococcus;	4.11E-05	4.11E-05	4.11E-05	3.61E-01	2.25E-01	1	2.75E-01	1.00	1	1.00	9.19E-01	1.00
Streptococcus	p__Firmicutes;c__Bacilli;o__Lactobacillales;f__Streptococcaceae;g__Streptococcus;	0.139814	0.084028	0.087397	5.36E-02	2.75E-02	1	1.76E-01	1.60	1	0.96	4.72E-01	1.66
Clostridia	p__Firmicutes;c__Clostridia;	0.089102	0.044325	0.048833	1.70E-07	6.10E-07	8.22E-05	4.63E-03	1.82	1	0.91	1.13E-01	2.01
Clostridiales	p__Firmicutes;c__Clostridia;o__Clostridiales;	0.089102	0.044325	0.048833	2.40E-07	1.15E-06	8.85E-05	2.97E-03	1.82	1	0.91	1.44E-01	2.01

Christensenellaceae	p__Firmicutes;c__Clostridia; o__Clostridiales;f__Christensenellaceae;	4.11E-05	4.11E-05	4.11E-05	5.90E-02	1.10E-01	5.26E-02	8.06E-01	1.00	1	1.00	7.92E-01	1.00
Christensenellaceae R-7 group	p__Firmicutes;c__Clostridia; o__Clostridiales;f__Christensenellaceae;g__Christensenellaceae_R-7_group;	5.14E-05	4.11E-05	4.11E-05	3.09E-02	4.47E-02	5.57E-02	3.40E-01	1.25	1	1.00	9.14E-01	1.25
Clostridiaceae 1	p__Firmicutes;c__Clostridia; o__Clostridiales;f__Clostridiaceae_1;	6.16E-05	2.05E-05	6.16E-05	5.27E-02	6.98E-01	1.19E-03	1	1.00	1	0.33	1	3.00
Clostridium sensu stricto 1	p__Firmicutes;c__Clostridia; o__Clostridiales;f__Clostridiaceae_1;g__Clostridium_sensu_stricto_1;	4.11E-05	2.05E-05	4.11E-05	1.59E-01	6.47E-01	1.48E-01	6.75E-01	1.00	1	0.50	1	2.00
Clostridiales vadinBB60 group	p__Firmicutes;c__Clostridia; o__Clostridiales;f__Clostridiales_vadinBB60_group;	0.000185	4.11E-05	6.16E-05	6.69E-07	7.76E-05	5.04E-13	1.03E-02	3.00	1	0.67	1.83E-02	4.50
unidentified Clostridiales vadinBB60 group	p__Firmicutes;c__Clostridia; o__Clostridiales;f__Clostridiales_vadinBB60_group;g__unidentified_Clostridiales_vadinBB60_group;	0.000185	4.11E-05	6.16E-05	3.28E-06	4.28E-04	1.03E-12	4.59E-02	3.00	1	0.67	7.05E-03	4.50
Defluviitaleaceae	p__Firmicutes;c__Clostridia; o__Clostridiales;f__Defluviitaleaceae;	0.001356	0.000246	0.00076	8.58E-10	1.05E-02	4.83E-10	1.71E-02	1.78	4.33E-01	0.32	2.00E-03	5.50

Defluviitaleaceae UCG-011	p__Firmicutes;c__Clostridia; o__Clostridiales;f__Defluviitaleaceae;g__Defluviitaleaceae_UCG-011;	0.001356	0.000246	0.00076	1.42E-09	9.82E-03	1.27E-10	1.88E-02	1.78	5.16E-01	0.32	5.48E-04	5.50
Family XI-1	p__Firmicutes;c__Clostridia; o__Clostridiales;f__Family_XI;	0.002239	0.00076	0.001181	9.06E-07	5.45E-03	2.76E-02	1.60E-02	1.90	4.16E-01	0.64	6.08E-04	2.95
Parvimonas	p__Firmicutes;c__Clostridia; o__Clostridiales;f__Family_XI;g__Parvimonas;	0.002116	0.00076	0.00114	1.23E-06	6.16E-03	1.91E-02	3.26E-02	1.86	3.62E-01	0.67	1.30E-04	2.78
unidentified Family XI	p__Firmicutes;c__Clostridia; o__Clostridiales;f__Family_XI;g__unidentified_Family_XI;	5.14E-05	2.05E-05	4.11E-05	2.75E-01	5.14E-01	1	1	1.25	8.37E-01	0.50	1	2.50
Family XIII	p__Firmicutes;c__Clostridia; o__Clostridiales;f__Family_XIII;	0.007374	0.002999	0.003471	3.46E-08	1.05E-06	5.04E-04	6.17E-04	2.12	1	0.86	7.73E-03	2.46
Eubacterium brachy group	p__Firmicutes;c__Clostridia; o__Clostridiales;f__Family_XIII;g__Eubacterium_brachy_group;	0.000144	6.16E-05	0.000103	2.40E-04	3.54E-02	1	2.42E-01	1.40	4.88E-01	0.60	5.26E-03	2.33
Eubacterium nodatum group	p__Firmicutes;c__Clostridia; o__Clostridiales;f__Family_XIII;g__Eubacterium_nodatum_group;	0.001828	0.001315	0.000976	5.74E-06	1.58E-06	6.48E-04	4.17E-02	1.87	1	1.35	9.91E-01	1.39

Family XIII UCG-001	p__Firmicutes;c__Clostridia; o__Clostridiales;f__Family_ XIII;g__Family_XIII_UCG-001;	0.000267	0.000123	0.000123	5.60E-08	1.19E-07	6.78E-05	1.07E-03	2.17	7.54E-01	1.00	7.08E-02	2.17
Mogibacterium	p__Firmicutes;c__Clostridia; o__Clostridiales;f__Family_ XIII;g__Mogibacterium;	0.000493	0.000246	0.000205	1.31E-07	1.04E-07	7.35E-06	5.17E-02	2.40	1	1.20	4.01E-01	2.00
unidentified Family XIII	p__Firmicutes;c__Clostridia; o__Clostridiales;f__Family_ XIII;g__unidentified_Family_ XIII;	0.001972	0.000657	0.000822	2.20E-07	5.37E-06	1.44E-05	3.61E-02	2.40	8.51E-01	0.80	1.14E-01	3.00
Lachnospiracea e	p__Firmicutes;c__Clostridia; o__Clostridiales;f__Lachnosp iraceae;	0.053712	0.025962	0.025603	8.39E-07	7.62E-07	1.20E-04	9.33E-06	2.10	6.59E-01	1.01	2.82E-01	2.07
Anaerostipes	p__Firmicutes;c__Clostridia; o__Clostridiales;f__Lachnosp iraceae;g__Anaerostipes;	4.11E-05	2.05E-05	4.11E-05	2.99E-01	5.94E-01	1	8.47E-01	1.00	1	0.50	1	2.00
Blautia	p__Firmicutes;c__Clostridia; o__Clostridiales;f__Lachnosp iraceae;g__Blautia;	8.22E-05	3.08E-05	4.11E-05	1.11E-04	1.16E-02	3.37E-03	2.01E-02	2.00	1	0.75	7.00E-02	2.67
Butyrivibrio 2	p__Firmicutes;c__Clostridia; o__Clostridiales;f__Lachnosp iraceae;g__Butyrivibrio_2;	0.0023	0.002198	0.001684	1.22E-01	1.04E-01	1	3.95E-01	1.37	9.47E-01	1.30	1	1.05
Catonella	p__Firmicutes;c__Clostridia; o__Clostridiales;f__Lachnosp iraceae;g__Catonella;	0.002855	0.001602	0.00114	1.99E-07	5.81E-08	2.11E-08	3.62E-03	2.50	1.67E-01	1.41	6.19E-01	1.78

Coprococcus 2	p__Firmicutes;c__Clostridia; o__Clostridiales;f__Lachnospiraceae;g__Coprococcus_2;	0.000113	2.05E-05	6.16E-05	4.50E-01	6.99E-01	0	1	1.83	NA	0.33	NA	5.51
Eubacterium ruminantium group	p__Firmicutes;c__Clostridia; o__Clostridiales;f__Lachnospiraceae;g__Eubacterium_ruminantium_group;	0.000185	2.05E-05	4.11E-05	2.62E-04	1.30E-02	1	7.06E-02	4.50	7.68E-01	0.50	7.41E-01	9.02
Eubacterium ventriosum group	p__Firmicutes;c__Clostridia; o__Clostridiales;f__Lachnospiraceae;g__Eubacterium_ventriosum_group;	5.14E-05	2.05E-05	3.08E-05	1.83E-01	2.75E-01	0	6.85E-01	1.67	NA	0.67	NA	2.50
Johnsonella	p__Firmicutes;c__Clostridia; o__Clostridiales;f__Lachnospiraceae;g__Johnsonella;	0.000616	0.000349	0.000359	8.70E-03	6.19E-03	1	4.43E-02	1.71	1	0.97	1.14E-01	1.76
Lachnoanaerobaculum	p__Firmicutes;c__Clostridia; o__Clostridiales;f__Lachnospiraceae;g__Lachnoanaerobaculum;	0.002629	0.001746	0.001602	2.62E-03	1.03E-03	1	6.74E-02	1.64	1	1.09	3.59E-02	1.51
Lachnoclostridium	p__Firmicutes;c__Clostridia; o__Clostridiales;f__Lachnospiraceae;g__Lachnoclostridium;	4.11E-05	4.11E-05	2.05E-05	2.86E-01	2.75E-01	2.65E-01	7.08E-01	2.00	1	2.00	1	1.00
Lachnospira	p__Firmicutes;c__Clostridia; o__Clostridiales;f__Lachnospiraceae;g__Lachnospira;	8.22E-05	2.05E-05	0.000103	6.64E-03	6.61E-01	1	7.51E-01	0.80	1	0.20	1	4.01

Lachnospiraceae NK3A20 group	p__Firmicutes;c__Clostridia; o__Clostridiales;f__Lachnospiraceae;g__Lachnospiraceae_NK3A20_group;	0.000123	2.05E-05	3.08E-05	6.02E-02	5.06E-02	0	1	4.00	NA	0.67	NA	6.01
Lachnospiraceae NK4A136 group	p__Firmicutes;c__Clostridia; o__Clostridiales;f__Lachnospiraceae;g__Lachnospiraceae_NK4A136_group;	6.16E-05	4.11E-05	9.24E-05	3.46E-05	4.66E-02	1.92E-02	2.01E-02	0.67	3.62E-01	0.44	4.52E-01	1.50
Moryella	p__Firmicutes;c__Clostridia; o__Clostridiales;f__Lachnospiraceae;g__Moryella;	2.05E-05	3.08E-05	2.05E-05	7.88E-01	6.61E-01	1	1	1.00	1	1.50	1	0.67
Oribacterium	p__Firmicutes;c__Clostridia; o__Clostridiales;f__Lachnospiraceae;g__Oribacterium;	0.035945	0.014891	0.015076	1.95E-07	8.17E-07	4.06E-06	2.49E-03	2.38	1	0.99	1.07E-01	2.41
Pseudobutyrivibrio	p__Firmicutes;c__Clostridia; o__Clostridiales;f__Lachnospiraceae;g__Pseudobutyrivibrio;	6.16E-05	4.11E-05	6.16E-05	2.89E-02	6.24E-01	1	1	1.00	1	0.67	9.14E-01	1.50
Roseburia	p__Firmicutes;c__Clostridia; o__Clostridiales;f__Lachnospiraceae;g__Roseburia;	7.19E-05	6.16E-05	0.000113	2.12E-03	4.04E-02	5.44E-01	6.93E-01	0.64	1	0.55	1	1.17
Shuttleworthia	p__Firmicutes;c__Clostridia; o__Clostridiales;f__Lachnospiraceae;g__Shuttleworthia;	0.000123	8.22E-05	6.16E-05	1.95E-03	9.86E-04	1.22E-02	9.30E-02	2.00	4.48E-01	1.33	1.94E-01	1.50

Stomatobaculum	p__Firmicutes;c__Clostridia;o__Clostridiales;f__Lachnospiraceae;g__Stomatobaculum;	0.000965	0.000575	0.000288	1.73E-09	1.31E-10	1.29E-08	1.91E-05	3.36	8.60E-02	2.00	1.91E-01	1.68
unidentified Lachnospiraceae	p__Firmicutes;c__Clostridia;o__Clostridiales;f__Lachnospiraceae;g__unidentified_Lachnospiraceae;	6.16E-05	4.11E-05	5.14E-05	1.04E-01	5.79E-01	1	1	1.20	1	0.80	8.40E-01	1.50
Peptococcaceae	p__Firmicutes;c__Clostridia;o__Clostridiales;f__Peptococcaceae;	0.000288	0.000144	0.000246	8.20E-05	2.59E-01	1	1	1.17	7.76E-01	0.58	1.30E-01	2.00
Peptococcus	p__Firmicutes;c__Clostridia;o__Clostridiales;f__Peptococcaceae;g__Peptococcus;	0.000267	0.000144	0.000236	1.75E-04	2.75E-01	1	9.65E-01	1.13	7.27E-01	0.61	1.14E-01	1.86
Peptostreptococcaceae	p__Firmicutes;c__Clostridia;o__Clostridiales;f__Peptostreptococcaceae;	0.012283	0.004252	0.005648	1.60E-06	1.22E-04	3.81E-02	1.02E-01	2.17	7.33E-01	0.75	7.70E-02	2.89
Filifactor	p__Firmicutes;c__Clostridia;o__Clostridiales;f__Peptostreptococcaceae;g__Filifactor;	0.001602	0.000246	0.000709	1.84E-10	9.02E-02	1.50E-11	7.06E-02	2.26	4.24E-02	0.35	5.28E-03	6.50
Peptoclostridium	p__Firmicutes;c__Clostridia;o__Clostridiales;f__Peptostreptococcaceae;g__Peptoclostridium;	0.004272	0.001253	0.001561	6.16E-08	2.31E-05	2.60E-06	3.07E-03	2.74	1	0.80	1.98E-02	3.41
Peptostreptococcus	p__Firmicutes;c__Clostridia;o__Clostridiales;f__Peptostre	0.004416	0.002547	0.002455	5.99E-03	2.75E-03	1	6.43E-02	1.80	1	1.04	9.89E-01	1.73

	ptococcaceae;g__Peptostreptococcus;												
Romboutsia	p__Firmicutes;c__Clostridia;o__Clostridiales;f__Peptostreptococcaceae;g__Romboutsia;	4.11E-05	2.05E-05	4.11E-05	6.53E-01	9.87E-01	0	1	1.00	7.54E-01	0.50	1	2.00
Ruminococcaceae	p__Firmicutes;c__Clostridia;o__Clostridiales;f__Ruminococcaceae;	0.007867	0.004293	0.005587	1.40E-02	1.46E-02	1	5.05E-01	1.41	7.76E-01	0.77	9.67E-02	1.83
Eubacterium coprostanoligenes group	p__Firmicutes;c__Clostridia;o__Clostridiales;f__Ruminococcaceae;g__Eubacterium_coprostanoligenes_group;	5.14E-05	4.11E-05	4.11E-05	3.42E-02	1.14E-01	1	8.18E-01	1.25	1	1.00	9.14E-01	1.25
Faecalibacterium	p__Firmicutes;c__Clostridia;o__Clostridiales;f__Ruminococcaceae;g__Faecalibacterium;	7.19E-05	0.000103	6.16E-05	2.54E-01	5.55E-01	1.06E-06	5.96E-01	1.17	1	1.67	1	0.70
Ruminiclostridium 5	p__Firmicutes;c__Clostridia;o__Clostridiales;f__Ruminococcaceae;g__Ruminiclostridium_5;	0.000103	4.11E-05	2.05E-05	2.00E-01	1.23E-01	1	9.65E-01	5.01	9.29E-01	2.00	8.62E-01	2.50
Ruminiclostridium 6	p__Firmicutes;c__Clostridia;o__Clostridiales;f__Ruminococcaceae;g__Ruminiclostridium_6;	9.24E-05	2.05E-05	8.22E-05	6.74E-02	7.37E-01	1	1	1.12	6.42E-01	0.25	9.89E-01	4.51

Ruminiclostridium 9	p__Firmicutes;c__Clostridia; o__Clostridiales;f__Ruminococcaceae;g__Ruminiclostridium_9;	4.11E-05	4.11E-05	4.11E-05	8.29E-01	7.05E-01	0	1	1.00	1	1.00	1	1.00
Ruminococcaceae NK4A214 group	p__Firmicutes;c__Clostridia; o__Clostridiales;f__Ruminococcaceae;g__Ruminococcaceae_NK4A214_group;	8.22E-05	2.05E-05	4.11E-05	2.52E-02	4.43E-02	7.75E-01	1	2.00	1	0.50	9.58E-01	4.01
Ruminococcaceae UCG-002	p__Firmicutes;c__Clostridia; o__Clostridiales;f__Ruminococcaceae;g__Ruminococcaceae_UCG-002;	6.16E-05	4.11E-05	4.11E-05	1.28E-01	2.98E-01	1	1	1.50	1	1.00	8.93E-01	1.50
Ruminococcaceae UCG-005	p__Firmicutes;c__Clostridia; o__Clostridiales;f__Ruminococcaceae;g__Ruminococcaceae_UCG-005;	0.000123	2.05E-05	0.000103	2.36E-01	5.23E-01	0	1	1.20	NA	0.20	NA	6.01
Ruminococcaceae UCG-010	p__Firmicutes;c__Clostridia; o__Clostridiales;f__Ruminococcaceae;g__Ruminococcaceae_UCG-010;	0.000123	2.05E-05	2.05E-05	2.71E-02	1.71E-02	0	9.57E-01	6.01	NA	1.00	NA	6.01
Ruminococcaceae UCG-014	p__Firmicutes;c__Clostridia; o__Clostridiales;f__Ruminococcaceae;g__Ruminococcaceae_UCG-014;	0.007251	0.00417	0.005443	5.31E-02	3.48E-02	1	5.64E-01	1.33	7.54E-01	0.77	1.56E-01	1.74
Ruminococcus 1	p__Firmicutes;c__Clostridia; o__Clostridiales;f__Ruminoc	8.21E-05	2.05E-05	4.11E-05	9.05E-02	2.75E-01	0	9.65E-01	2.00	NA	0.50	NA	4.01

	occaceae;g__Ruminococcus_1;												
Ruminococcus 2	p__Firmicutes;c__Clostridia;o__Clostridiales;f__Ruminococcaceae;g__Ruminococcus_2;	6.16E-05	3.08E-05	4.11E-05	6.28E-03	1.72E-02	1	6.93E-01	1.50	1	0.75	1	2.00
Subdoligranulum	p__Firmicutes;c__Clostridia;o__Clostridiales;f__Ruminococcaceae;g__Subdoligranulum;	4.11E-05	2.05E-05	4.11E-05	1.66E-01	7.64E-01	1	1	1.00	1	0.50	9.14E-01	2.00
Erysipelotrichia	p__Firmicutes;c__Erysipelotrichia;	0.003286	0.00189	0.001797	5.24E-05	4.42E-05	1.19E-01	2.68E-02	1.83	1	1.05	1.17E-01	1.74
Erysipelotrichales	p__Firmicutes;c__Erysipelotrichia;o__Erysipelotrichales;	0.003286	0.00189	0.001797	5.81E-05	7.50E-05	1.12E-01	2.45E-02	1.83	1	1.05	1.24E-01	1.74
Erysipelotrichaceae	p__Firmicutes;c__Erysipelotrichia;o__Erysipelotrichales;f__Erysipelotrichaceae;	0.003286	0.00189	0.001797	5.79E-05	7.76E-05	2.46E-01	2.05E-02	1.83	1	1.05	6.62E-02	1.74
Bulleidia	p__Firmicutes;c__Erysipelotrichia;o__Erysipelotrichales;f__Erysipelotrichaceae;g__Bulleidia;	6.16E-05	4.11E-05	4.11E-05	1.66E-02	2.69E-02	1	3.04E-01	1.50	6.70E-01	1.00	9.59E-01	1.50
Eggerthia	p__Firmicutes;c__Erysipelotrichia;o__Erysipelotrichales;f__Erysipelotrichaceae;g__Eggerthia;	4.11E-05	4.11E-05	4.11E-05	8.48E-01	7.19E-01	1	1	1.00	1	1.00	1	1.00

Erysipelotrichaceae UCG-006	p__Firmicutes;c__Erysipelotrichia;o__Erysipelotrichales;f__Erysipelotrichaceae;g__Erysipelotrichaceae_UCG-006;	0.001089	0.00039	0.000493	4.04E-05	1.03E-03	1.20E-01	1.53E-02	2.21	6.67E-01	0.79	7.66E-04	2.79
Solobacterium	p__Firmicutes;c__Erysipelotrichia;o__Erysipelotrichales;f__Erysipelotrichaceae;g__Solobacterium;	0.001787	0.001356	0.001006	3.94E-04	1.40E-04	1.20E-01	4.76E-02	1.78	4.78E-01	1.35	7.74E-01	1.32
Negativicutes	p__Firmicutes;c__Negativicutes;	0.033624	0.063571	0.022214	1.40E-06	3.37E-02	1.67E-09	2.33E-01	1.51	8.60E-02	2.86	5.08E-01	0.53
Selenomonadales	p__Firmicutes;c__Negativicutes;o__Selenomonadales;	0.033624	0.063571	0.022214	2.31E-06	4.70E-02	6.16E-09	2.41E-01	1.51	8.10E-02	2.86	4.02E-01	0.53
Acidaminococcaceae	p__Firmicutes;c__Negativicutes;o__Selenomonadales;f__Acidaminococcaceae;	4.11E-05	2.05E-05	4.11E-05	2.41E-02	2.22E-01	1	1	1.00	1	0.50	7.92E-01	2.00
Veillonellaceae	p__Firmicutes;c__Negativicutes;o__Selenomonadales;f__Veillonellaceae;	0.033541	0.06355	0.022214	1.60E-06	4.18E-02	8.89E-08	1.63E-01	1.51	1.10E-01	2.86	4.17E-01	0.53
Anaeroglobus	p__Firmicutes;c__Negativicutes;o__Selenomonadales;f__Veillonellaceae;g__Anaeroglobus;	6.16E-05	4.11E-05	4.11E-05	4.43E-02	6.75E-02	1.04E-01	3.18E-01	1.50	1	1.00	7.41E-01	1.50
Dialister	p__Firmicutes;c__Negativicutes;o__Selenomonadales;f__Veillonellaceae;g__Dialister;	0.000883	0.00076	0.000668	1.52E-01	6.75E-02	1	4.83E-01	1.32	9.47E-01	1.14	1	1.16

Megasphaera	p__Firmicutes;c__Negativicutes;o__Selenomonadales;f__Veillonellaceae;g__Megasphaera;	0.000236	0.000246	0.000103	3.02E-04	4.08E-03	1.89E-06	4.00E-02	2.30	3.85E-02	2.40	1	0.96
Selenomonas	p__Firmicutes;c__Negativicutes;o__Selenomonadales;f__Veillonellaceae;g__Selenomonas;	0.003512	0.005936	0.002013	3.46E-05	4.04E-02	1.03E-05	7.44E-02	1.74	3.70E-03	2.95	1.12E-02	0.59
Selenomonas 3	p__Firmicutes;c__Negativicutes;o__Selenomonadales;f__Veillonellaceae;g__Selenomonas_3;	0.01103	0.019903	0.006953	1.07E-05	1.67E-01	3.68E-06	2.84E-01	1.59	2.35E-02	2.86	4.01E-01	0.55
Selenomonas 4	p__Firmicutes;c__Negativicutes;o__Selenomonadales;f__Veillonellaceae;g__Selenomonas_4;	0.002506	0.003656	0.000945	3.97E-09	3.63E-06	1.65E-11	4.05E-03	2.65	1.02E-02	3.87	3.18E-01	0.69
unidentified Veillonellaceae	p__Firmicutes;c__Negativicutes;o__Selenomonadales;f__Veillonellaceae;g__unidentified_Veillonellaceae;	7.19E-05	8.22E-05	6.16E-05	4.02E-01	4.30E-01	8.50E-04	1	1.17	5.16E-01	1.33	9.84E-01	0.87
Veillonella	p__Firmicutes;c__Negativicutes;o__Selenomonadales;f__Veillonellaceae;g__Veillonella;	0.013947	0.023703	0.007939	4.37E-07	1.03E-02	1.04E-08	4.57E-02	1.76	2.27E-02	2.99	1.84E-01	0.59
unidentified Firmicutes	p__Firmicutes;c__unidentified_Firmicutes;	0.000349	0.000123	0.000103	6.71E-07	2.24E-07	3.50E-05	3.38E-04	3.40	9.92E-01	1.20	8.24E-02	2.83

Clostridiales-1	p__Firmicutes;c__unidentified_Firmicutes;o__Clostridiales;	0.000349	0.000123	0.000103	1.04E-06	3.46E-07	3.52E-05	1.61E-03	3.40	7.14E-01	1.20	9.38E-02	2.83
Family XIII-1	p__Firmicutes;c__unidentified_Firmicutes;o__Clostridiales;f__Family_XIII;	0.000349	0.000103	8.22E-05	2.16E-07	1.69E-07	5.03E-05	3.21E-03	4.25	1	1.25	9.92E-02	3.40
Lachnospiraceae-1	p__Firmicutes;c__unidentified_Firmicutes;o__Clostridiales;f__Lachnospiraceae;	4.11E-05	4.11E-05	4.11E-05	7.81E-01	6.98E-01	1	1	1.00	1	1.00	1	1.00
Fusobacteria	p__Fusobacteria;	0.013823	0.007333	0.005597	6.80E-05	9.09E-06	1.30E-01	1.04E-03	2.47	7.01E-02	1.31	9.31E-02	1.89
Fusobacteriia	p__Fusobacteria;c__Fusobacteriia;	0.013823	0.007333	0.005597	7.87E-05	1.20E-05	6.13E-02	5.33E-04	2.47	1.47E-01	1.31	1.13E-01	1.89
Fusobacteriales	p__Fusobacteria;c__Fusobacteriia;o__Fusobacteriales;	0.013823	0.007333	0.005597	9.07E-05	2.02E-05	6.93E-02	2.67E-03	2.47	1.17E-01	1.31	1.24E-01	1.89
Fusobacteriaceae	p__Fusobacteria;c__Fusobacteriia;o__Fusobacteriales;f__Fusobacteriaceae;	0.009797	0.005053	0.004406	7.74E-05	3.37E-05	3.40E-01	1.27E-02	2.22	2.57E-01	1.15	1.55E-01	1.94
Cetobacterium	p__Fusobacteria;c__Fusobacteriia;o__Fusobacteriales;f__Fusobacteriaceae;g__Cetobacterium;	2.05E-05	6.16E-05	6.16E-05	2.39E-02	1.41E-02	0	7.54E-02	0.33	NA	1.00	NA	0.33
Fusobacterium	p__Fusobacteria;c__Fusobacteriia;o__Fusobacteriales;f__Fusobacteriaceae;g__Fusobacterium;	0.009797	0.005053	0.004406	7.14E-05	3.05E-05	1.48E-01	8.04E-03	2.22	1.55E-01	1.15	1.39E-01	1.94

Leptotrichiaceae	p__Fusobacteria;c__Fusobacteriia;o__Fusobacteriales;f__Leptotrichiaceae;	0.002547	0.002136	0.001089	2.33E-03	7.68E-04	5.63E-01	3.01E-03	2.34	8.29E-02	1.96	4.06E-01	1.19
Leptotrichia	p__Fusobacteria;c__Fusobacteriia;o__Fusobacteriales;f__Leptotrichiaceae;g__Leptotrichia;	0.002547	0.002136	0.001048	1.59E-03	5.10E-04	2.54E-01	6.72E-03	2.43	4.95E-02	2.04	7.41E-01	1.19
Streptobacillus	p__Fusobacteria;c__Fusobacteriia;o__Fusobacteriales;f__Leptotrichiaceae;g__Streptobacillus;	4.11E-05	4.11E-05	6.16E-05	2.51E-01	1.66E-01	3.50E-05	1	0.67	9.24E-01	0.67	9.66E-01	1.00
unidentified Leptotrichiaceae	p__Fusobacteria;c__Fusobacteriia;o__Fusobacteriales;f__Leptotrichiaceae;g__unidentified_Leptotrichiaceae;	6.16E-05	4.11E-05	4.11E-05	3.54E-01	2.25E-01	0	6.36E-01	1.50	1	1.00	1	1.50
Gemmatimonadetes	p__Gemmatimonadetes;	6.16E-05	4.11E-05	8.22E-05	3.27E-02	9.80E-01	8.18E-01	8.81E-01	0.75	1	0.50	8.47E-01	1.50
unidentified Gemmatimonadetes	p__Gemmatimonadetes;c__unidentified_Gemmatimonadetes;	6.16E-05	4.11E-05	8.22E-05	3.63E-02	9.80E-01	1	9.22E-01	0.75	1	0.50	9.84E-01	1.50
Gemmatimonadales	p__Gemmatimonadetes;c__unidentified_Gemmatimonadetes;o__Gemmatimonadales;	7.19E-05	4.11E-05	8.22E-05	3.73E-02	8.32E-01	1	7.12E-01	0.87	1	0.50	1	1.75
Gemmatimonadaceae	p__Gemmatimonadetes;c__unidentified_Gemmatimonadet	7.19E-05	4.11E-05	8.22E-05	3.94E-02	8.04E-01	1	7.46E-01	0.87	1	0.50	1	1.75

	es;o__Gemmatimonadales;f__Gemmatimonadaceae;												
Gemmatimonas	p__Gemmatimonadetes;c__unidentified_Gemmatimonadetes;o__Gemmatimonadales;f__Gemmatimonadaceae;g__Gemmatimonas;	6.16E-05	2.05E-05	6.16E-05	3.38E-01	7.31E-01	1	9.16E-01	1.00	8.96E-01	0.33	1	3.00
unidentified Gemmatimonadetes	p__Gemmatimonadetes;c__unidentified_Gemmatimonadetes;o__unidentified_Gemmatimonadetes;	4.11E-05	5.14E-05	0.000123	4.33E-01	4.00E-01	1	4.50E-01	0.33	6.70E-01	0.42	1	0.80
S0134 terrestrial group	p__Gemmatimonadetes;c__unidentified_Gemmatimonadetes;o__unidentified_Gemmatimonadetes;f__S0134_terrestrial_group;	4.11E-05	6.16E-05	0.000175	4.95E-01	5.11E-01	0	7.23E-01	0.24	7.76E-01	0.35	1	0.67
Gracilibacteria	p__Gracilibacteria;	0.000103	0.000113	0.000185	1.68E-01	8.84E-02	4.07E-02	1.29E-01	0.56	7.15E-01	0.61	1	0.91
unidentified Gracilibacteria	p__Gracilibacteria;c__unidentified_Gracilibacteria;	0.000103	0.000113	0.000185	2.05E-01	9.82E-02	1.75E-01	2.17E-01	0.56	9.92E-01	0.61	1	0.91
unidentified Gracilibacteria	p__Gracilibacteria;c__unidentified_Gracilibacteria;o__unidentified_Gracilibacteria;	0.000103	0.000113	0.000185	1.93E-01	1.22E-01	4.97E-01	2.24E-01	0.56	7.43E-01	0.61	1	0.91
unidentified Gracilibacteria	p__Gracilibacteria;c__unidentified_Gracilibacteria;o__unidentified_Gracilibacteria;f__unidentified_Gracilibacteria;	0.000103	0.000113	0.000185	1.86E-01	1.12E-01	7.90E-01	2.17E-01	0.56	8.14E-01	0.61	1	0.91

unidentified Gracilibacteria	p__Gracilibacteria;c__unidentified_Gracilibacteria;o__unidentified_Gracilibacteria;f__unidentified_Gracilibacteria;g__unidentified_Gracilibacteria;	0.000103	0.000113	0.000185	1.70E-01	9.25E-02	1	2.46E-01	0.56	8.96E-01	0.61	1	0.91
Latescibacteria	p__Latescibacteria;	4.11E-05	0	8.22E-05	6.30E-01	6.73E-01	0	8.83E-01	0.50	NA	NA	NA	NA
Nitrospirae	p__Nitrospirae;	6.16E-05	3.08E-05	8.22E-05	9.58E-02	9.42E-01	1.17E-01	8.83E-01	0.75	1	0.37	1	2.00
Nitrospira	p__Nitrospirae;c__Nitrospira;	6.16E-05	3.08E-05	8.22E-05	1.11E-01	9.78E-01	8.35E-01	9.95E-01	0.75	1	0.37	1	2.00
Nitrospirales	p__Nitrospirae;c__Nitrospira;o__Nitrospirales;	6.16E-05	3.08E-05	8.22E-05	1.05E-01	9.80E-01	1	1	0.75	1	0.37	1	2.00
0319-6A21	p__Nitrospirae;c__Nitrospira;o__Nitrospirales;f__0319-6A21;	6.16E-05	2.05E-05	4.11E-05	3.58E-01	4.32E-01	0	6.63E-02	1.50	1	0.50	9.74E-01	3.00
Nitrospiraceae	p__Nitrospirae;c__Nitrospira;o__Nitrospirales;f__Nitrospiraceae;	6.16E-05	3.08E-05	4.11E-05	3.54E-01	9.69E-01	1	1	1.50	1	0.75	7.06E-01	2.00
unidentified Nitrospiraceae	p__Nitrospirae;c__Nitrospira;o__Nitrospirales;f__Nitrospiraceae;g__unidentified_Nitrospiraceae;	6.16E-05	2.05E-05	9.24E-05	2.99E-01	8.19E-01	0	1	0.67	8.96E-01	0.22	8.62E-01	3.00
Planctomycetes	p__Planctomycetes;	6.16E-05	0.000205	6.16E-05	9.14E-01	7.86E-01	7.54E-02	8.81E-01	1.00	1	3.33	2.73E-01	0.30
OM190	p__Planctomycetes;c__OM190;	4.11E-05	2.05E-05	4.11E-05	5.39E-01	6.04E-01	0	9.22E-01	1.00	NA	0.50	NA	2.00
Planctomycetacia	p__Planctomycetes;c__Planctomycetacia;	6.16E-05	0.000164	9.24E-05	8.29E-01	7.38E-01	1	9.22E-01	0.67	1	1.78	2.65E-01	0.37

Planctomycetales	p__Planctomycetes;c__Planctomycetacia;o__Planctomycetales;	6.16E-05	0.000164	9.24E-05	9.37E-01	8.95E-01	1	1	0.67	1	1.78	4.02E-01	0.37
Planctomycetaceae	p__Planctomycetes;c__Planctomycetacia;o__Planctomycetales;f__Planctomycetaceae;	6.16E-05	0.000164	9.24E-05	9.37E-01	8.67E-01	1	1	0.67	1	1.78	2.33E-01	0.37
Proteobacteria	p__Proteobacteria;	0.39346	0.440681	0.591114	2.86E-05	9.09E-06	3.60E-03	3.73E-03	0.67	7.01E-02	0.75	1	0.89
Alphaproteobacteria	p__Proteobacteria;c__Alphaproteobacteria;	0.0038	0.003677	0.005967	1.28E-01	8.85E-02	7.43E-01	2.39E-01	0.64	9.92E-01	0.62	1	1.03
Caulobacterales	p__Proteobacteria;c__Alphaproteobacteria;o__Caulobacterales;	0.000637	0.000596	0.002537	1.08E-05	1.02E-04	9.28E-06	2.11E-01	0.25	4.45E-01	0.23	8.20E-01	1.07
Caulobacteraceae	p__Proteobacteria;c__Alphaproteobacteria;o__Caulobacterales;f__Caulobacteraceae;	0.000637	0.000575	0.002444	9.48E-06	1.20E-04	3.42E-06	2.65E-01	0.26	6.59E-01	0.24	7.06E-01	1.11
Brevundimonas	p__Proteobacteria;c__Alphaproteobacteria;o__Caulobacterales;f__Caulobacteraceae;g__Brevundimonas;	0.000431	0.000452	0.002342	8.12E-07	2.69E-06	1.40E-07	1.55E-01	0.18	5.16E-01	0.19	9.91E-01	0.95
Caulobacter	p__Proteobacteria;c__Alphaproteobacteria;o__Caulobacterales;f__Caulobacteraceae;g__Caulobacter;	8.22E-05	6.16E-05	4.11E-05	7.36E-03	2.65E-03	9.03E-06	2.39E-01	2.00	6.01E-01	1.50	6.92E-01	1.33
Phenylobacterium	p__Proteobacteria;c__Alphaproteobacteria;o__Caulobacter	6.16E-05	4.11E-05	6.16E-05	5.78E-01	7.05E-01	1	1	1.00	8.37E-01	0.67	9.69E-01	1.50

	ales;f__Caulobacteraceae;g__Phenylobacterium;												
Hyphomonadaceae	p__Proteobacteria;c__Alphaproteobacteria;o__Caulobacterales;f__Hyphomonadaceae;	2.05E-05	2.05E-05	4.11E-05	5.77E-01	5.14E-01	1	1	0.50	1	0.50	1	1.00
Rhizobiales	p__Proteobacteria;c__Alphaproteobacteria;o__Rhizobiales;	0.00113	0.002198	0.000883	4.52E-03	9.95E-01	3.22E-05	9.20E-01	1.28	7.49E-02	2.49	2.51E-01	0.51
Bartonellaceae	p__Proteobacteria;c__Alphaproteobacteria;o__Rhizobiales;f__Bartonellaceae;	0.000123	0	6.16E-05	5.82E-02	8.50E-02	0	5.38E-01	2.00	NA	NA	NA	NA
Bartonella	p__Proteobacteria;c__Alphaproteobacteria;o__Rhizobiales;f__Bartonellaceae;g__Bartonella;	0.000123	0	6.16E-05	5.60E-02	6.75E-02	0	4.18E-01	2.00	NA	NA	NA	NA
Bradyrhizobiaceae	p__Proteobacteria;c__Alphaproteobacteria;o__Rhizobiales;f__Bradyrhizobiaceae;	0.000123	6.16E-05	0.000103	6.02E-02	7.07E-01	1	1	1.20	5.29E-01	0.60	1	2.00
Bosea	p__Proteobacteria;c__Alphaproteobacteria;o__Rhizobiales;f__Bradyrhizobiaceae;g__Bosea;	6.16E-05	3.08E-05	4.11E-05	2.05E-01	2.56E-01	1	5.21E-01	1.50	9.96E-01	0.75	4.46E-01	2.00
Brucellaceae	p__Proteobacteria;c__Alphaproteobacteria;o__Rhizobiales;f__Brucellaceae;	4.11E-05	4.11E-05	6.16E-05	3.59E-01	3.06E-01	1	3.46E-01	0.67	8.13E-01	0.67	7.92E-01	1.00

Brucella	p__Proteobacteria;c__Alphaproteobacteria;o__Rhizobiales;f__Brucellaceae;g__Brucella;	4.11E-05	4.11E-05	6.16E-05	3.45E-01	2.69E-01	1	4.06E-01	0.67	7.68E-01	0.67	8.62E-01	1.00
Hyphomicrobiaceae	p__Proteobacteria;c__Alphaproteobacteria;o__Rhizobiales;f__Hyphomicrobiaceae;	8.22E-05	4.11E-05	0.000103	5.82E-02	7.11E-01	7.69E-01	8.06E-01	0.80	7.76E-01	0.40	3.70E-01	2.00
Devosia	p__Proteobacteria;c__Alphaproteobacteria;o__Rhizobiales;f__Hyphomicrobiaceae;g__Devosia;	4.11E-05	4.11E-05	0.000267	6.64E-03	4.16E-03	7.12E-03	6.13E-01	0.15	1	0.15	9.91E-01	1.00
Hyphomicrobium	p__Proteobacteria;c__Alphaproteobacteria;o__Rhizobiales;f__Hyphomicrobiaceae;g__Hyphomicrobium;	0.000123	4.11E-05	4.11E-05	6.39E-04	4.06E-04	7.57E-03	4.43E-02	3.00	1	1.00	1	3.00
Pedomicrobium	p__Proteobacteria;c__Alphaproteobacteria;o__Rhizobiales;f__Hyphomicrobiaceae;g__Pedomicrobium;	4.11E-05	2.05E-05	0.000411	6.02E-02	1.39E-01	0	7.71E-01	0.10	NA	0.05	NA	2.00
Methylobacteriaceae	p__Proteobacteria;c__Alphaproteobacteria;o__Rhizobiales;f__Methylobacteriaceae;	0.000431	0.001664	0.000288	8.58E-10	3.48E-01	6.35E-16	1	1.50	2.79E-02	5.79	6.01E-03	0.26
Methylobacterium	p__Proteobacteria;c__Alphaproteobacteria;o__Rhizobiales;f__Methylobacteriaceae;g__Methylobacterium;	0.00039	0.001664	0.000288	1.42E-09	3.66E-01	1.05E-14	9.65E-01	1.36	3.70E-03	5.79	4.02E-03	0.23

Microvirga	p__Proteobacteria;c__Alphaproteobacteria;o__Rhizobiales;f__Methylobacteriaceae;g__Microvirga;	4.11E-05	2.05E-05	4.11E-05	6.53E-01	9.29E-01	0	9.65E-01	1.00	NA	0.50	NA	2.00
Rhizobiaceae	p__Proteobacteria;c__Alphaproteobacteria;o__Rhizobiales;f__Rhizobiaceae;	6.16E-05	4.11E-05	6.16E-05	9.65E-02	2.76E-01	2.95E-02	1	1.00	1	0.67	7.06E-01	1.50
Ensifer	p__Proteobacteria;c__Alphaproteobacteria;o__Rhizobiales;f__Rhizobiaceae;g__Ensifer;	4.11E-05	2.05E-05	2.05E-05	7.77E-01	6.23E-01	1	1	2.00	1	1.00	1	2.00
Rhizobium	p__Proteobacteria;c__Alphaproteobacteria;o__Rhizobiales;f__Rhizobiaceae;g__Rhizobium;	4.11E-05	4.11E-05	6.16E-05	1.59E-01	1.27E-01	4.52E-02	1	0.67	1	0.67	1	1.00
unidentified Rhizobiales	p__Proteobacteria;c__Alphaproteobacteria;o__Rhizobiales;f__unidentified_Rhizobiales;	4.11E-05	2.05E-05	4.11E-05	2.99E-01	3.04E-01	1	7.46E-01	1.00	1	0.50	1	2.00
Rhizomicrobium	p__Proteobacteria;c__Alphaproteobacteria;o__Rhizobiales;f__unidentified_Rhizobiales;g__Rhizomicrobium;	4.11E-05	2.05E-05	3.08E-05	4.40E-01	4.27E-01	1	6.36E-01	1.33	1	0.67	8.62E-01	2.00
Xanthobacteraceae	p__Proteobacteria;c__Alphaproteobacteria;o__Rhizobiales;f__Xanthobacteraceae;	6.16E-05	2.05E-05	4.11E-05	2.30E-01	9.18E-01	1	1	1.50	1	0.50	1	3.00
Pseudolabrys	p__Proteobacteria;c__Alphaproteobacteria;o__Rhizobiales;	4.11E-05	2.05E-05	0.000452	4.02E-01	6.73E-01	0	1.69E-01	0.09	NA	0.05	NA	2.00

	f__Xanthobacteraceae;g__Pseudolabrys;												
Rhodobacterales	p__Proteobacteria;c__Alphaproteobacteria;o__Rhodobacterales;	0.000164	6.16E-05	0.000277	1.63E-04	2.36E-01	4.12E-01	6.55E-01	0.59	6.24E-02	0.22	4.33E-02	2.67
Rhodobacteraceae	p__Proteobacteria;c__Alphaproteobacteria;o__Rhodobacterales;f__Rhodobacteraceae;	0.000164	6.16E-05	0.000277	1.64E-04	2.43E-01	2.40E-01	6.60E-01	0.59	2.79E-02	0.22	4.54E-02	2.67
Amaricoccus	p__Proteobacteria;c__Alphaproteobacteria;o__Rhodobacterales;f__Rhodobacteraceae;g__Amaricoccus;	4.11E-05	3.08E-05	0.000164	1.11E-02	1.72E-02	2.53E-02	3.87E-01	0.25	1	0.19	1	1.33
Falsirhodobacter	p__Proteobacteria;c__Alphaproteobacteria;o__Rhodobacterales;f__Rhodobacteraceae;g__Falsirhodobacter;	2.05E-05	2.05E-05	3.08E-05	9.71E-01	9.51E-01	1	1	0.67	1	0.67	1	1.00
Loktanella	p__Proteobacteria;c__Alphaproteobacteria;o__Rhodobacterales;f__Rhodobacteraceae;g__Loktanella;	4.11E-05	0	2.05E-05	2.36E-01	2.93E-01	0	3.13E-01	2.00	NA	NA	NA	NA
Marivita	p__Proteobacteria;c__Alphaproteobacteria;o__Rhodobacterales;f__Rhodobacteraceae;g__Marivita;	4.11E-05	0	4.11E-05	2.99E-01	3.66E-01	0	6.33E-01	1.00	NA	NA	NA	NA
Paracocccus	p__Proteobacteria;c__Alphaproteobacteria;o__Rhodobacter	6.16E-05	4.11E-05	6.16E-05	8.29E-01	9.90E-01	1	9.61E-01	1.00	9.47E-01	0.67	1	1.50

	ales;f__Rhodobacteraceae;g__Paracocccus;												
Rubellimicrobium	p__Proteobacteria;c__Alphaproteobacteria;o__Rhodobacterales;f__Rhodobacteraceae;g__Rubellimicrobium;	4.11E-05	2.05E-05	4.11E-05	3.07E-01	5.15E-01	1	7.31E-01	1.00	1	0.50	1	2.00
Rhodospirillales	p__Proteobacteria;c__Alphaproteobacteria;o__Rhodospirillales;	9.24E-05	0.000103	0.000113	3.18E-01	3.76E-01	1	4.50E-01	0.82	1	0.91	1	0.90
Acetobacteraceae	p__Proteobacteria;c__Alphaproteobacteria;o__Rhodospirillales;f__Acetobacteraceae;	4.11E-05	4.11E-05	0.000185	1.81E-03	5.45E-03	1.54E-02	5.71E-01	0.22	8.14E-01	0.22	1	1.00
Gluconobacter	p__Proteobacteria;c__Alphaproteobacteria;o__Rhodospirillales;f__Acetobacteraceae;g__Gluconobacter;	2.05E-05	4.11E-05	0.000555	2.39E-02	7.10E-03	4.07E-03	9.65E-01	0.04	4.82E-03	0.07	1	0.50
DA111	p__Proteobacteria;c__Alphaproteobacteria;o__Rhodospirillales;f__DA111;	8.22E-05	5.14E-05	4.11E-05	7.25E-03	2.51E-03	1.76E-02	7.23E-01	2.00	7.76E-01	1.25	3.55E-01	1.60
Rhodospirillaceae	p__Proteobacteria;c__Alphaproteobacteria;o__Rhodospirillales;f__Rhodospirillaceae;	4.11E-05	4.11E-05	5.14E-05	5.46E-01	8.79E-01	1	1	0.80	1	0.80	8.35E-01	1.00
Skermanella	p__Proteobacteria;c__Alphaproteobacteria;o__Rhodospirillales;f__Rhodospirillaceae;g__Skermanella;	4.11E-05	2.05E-05	4.11E-05	3.37E-01	9.78E-01	1	1	1.00	1	0.50	9.14E-01	2.00

unidentified Rhodospirillales	p__Proteobacteria;c__Alphaproteobacteria;o__Rhodospirillales;f__unidentified_Rhodospirillales;	4.11E-05	2.05E-05	6.16E-05	5.66E-01	9.44E-01	1	1	0.67	1	0.33	1	2.00
Reyranella	p__Proteobacteria;c__Alphaproteobacteria;o__Rhodospirillales;f__unidentified_Rhodospirillales;g__Reyranella;	4.11E-05	2.05E-05	6.16E-05	4.84E-01	8.42E-01	1	1	0.67	1	0.33	6.51E-01	2.00
Rickettsiales	p__Proteobacteria;c__Alphaproteobacteria;o__Rickettsiales;	8.22E-05	4.11E-05	7.19E-05	2.76E-01	6.00E-01	1	2.11E-01	1.14	7.63E-01	0.57	5.54E-01	2.00
Anaplasmataceae	p__Proteobacteria;c__Alphaproteobacteria;o__Rickettsiales;f__Anaplasmataceae;	4.11E-05	0	6.16E-05	3.07E-01	3.94E-01	0	1	0.67	NA	NA	NA	NA
Wolbachia	p__Proteobacteria;c__Alphaproteobacteria;o__Rickettsiales;f__Anaplasmataceae;g__Wolbachia;	4.11E-05	0	6.16E-05	2.99E-01	3.49E-01	0	1	0.67	NA	NA	NA	NA
Mitochondria	p__Proteobacteria;c__Alphaproteobacteria;o__Rickettsiales;f__Mitochondria;	4.11E-05	4.11E-05	6.16E-05	8.31E-01	8.37E-01	1	1	0.67	1	0.67	8.57E-01	1.00
unidentified Mitochondria	p__Proteobacteria;c__Alphaproteobacteria;o__Rickettsiales;f__Mitochondria;g__unidentified_Mitochondria;	4.11E-05	4.11E-05	6.16E-05	8.29E-01	8.25E-01	1	1	0.67	1	0.67	9.91E-01	1.00

Sphingomonadales	p__Proteobacteria;c__Alphaproteobacteria;o__Sphingomonadales;	0.000965	0.000246	0.000288	1.04E-06	1.49E-04	8.42E-09	6.04E-03	3.36	7.14E-01	0.86	3.86E-02	3.92
Erythrobacteraceae	p__Proteobacteria;c__Alphaproteobacteria;o__Sphingomonadales;f__Erythrobacteraceae;	4.11E-05	2.05E-05	4.11E-05	2.48E-01	8.04E-01	1	1	1.00	9.72E-01	0.50	8.76E-01	2.00
Altererythrobacter	p__Proteobacteria;c__Alphaproteobacteria;o__Sphingomonadales;f__Erythrobacteraceae;g__Altererythrobacter;	2.05E-05	2.05E-05	4.11E-05	2.06E-01	1.72E-01	1	1	0.50	1	0.50	1	1.00
Sphingomonadaceae	p__Proteobacteria;c__Alphaproteobacteria;o__Sphingomonadales;f__Sphingomonadaceae;	0.000832	0.000195	0.000288	1.60E-06	1.57E-04	5.45E-08	1.60E-02	2.89	7.76E-01	0.68	2.69E-02	4.26
Blastomonas	p__Proteobacteria;c__Alphaproteobacteria;o__Sphingomonadales;f__Sphingomonadaceae;g__Blastomonas;	0.00037	9.24E-05	0.000123	5.38E-07	5.01E-04	5.10E-10	1.76E-01	3.00	3.62E-01	0.75	7.08E-02	4.00
Novosphingobium	p__Proteobacteria;c__Alphaproteobacteria;o__Sphingomonadales;f__Sphingomonadaceae;g__Novosphingobium;	7.19E-05	3.08E-05	2.05E-05	2.10E-02	1.41E-02	1	5.21E-01	3.51	1	1.50	1	2.33
Sphingomonas	p__Proteobacteria;c__Alphaproteobacteria;o__Sphingomonadales;f__Sphingomonadaceae;g__Sphingomonas;	0.000144	0.000103	0.000123	1.37E-01	2.55E-01	1	4.83E-01	1.17	6.01E-01	0.83	3.08E-02	1.40

Sphingopyxis	p__Proteobacteria;c__Alphaproteobacteria;o__Sphingomonadales;f__Sphingomonadaceae;g__Sphingopyxis;	4.11E-05	4.11E-05	6.16E-05	1.17E-01	9.72E-02	1	5.21E-01	0.67	8.37E-01	0.67	1	1.00
Betaproteobacteria	p__Proteobacteria;c__Betaproteobacteria;	0.182558	0.128702	0.152662	2.95E-01	7.84E-01	1	5.34E-01	1.20	1	0.84	2.65E-01	1.42
Burkholderiales	p__Proteobacteria;c__Betaproteobacteria;o__Burkholderiales;	0.029762	0.01713	0.027657	3.40E-03	2.04E-01	1	8.30E-01	1.08	2.48E-01	0.62	1.34E-02	1.74
Alcaligenaceae	p__Proteobacteria;c__Betaproteobacteria;o__Burkholderiales;f__Alcaligenaceae;	0.000123	8.22E-05	0.000103	3.89E-01	5.49E-01	1	1	1.20	1	0.80	7.06E-01	1.50
Burkholderiaceae	p__Proteobacteria;c__Betaproteobacteria;o__Burkholderiales;f__Burkholderiaceae;	0.028612	0.013187	0.014809	1.21E-04	1.57E-04	2.67E-02	6.45E-02	1.93	1	0.89	2.02E-02	2.17
Burkholderia	p__Proteobacteria;c__Betaproteobacteria;o__Burkholderiales;f__Burkholderiaceae;g__Burkholderia;	4.11E-05	4.11E-05	4.11E-05	6.16E-01	6.21E-01	1	1	1.00	5.67E-01	1.00	1	1.00
Cupriavidus	p__Proteobacteria;c__Betaproteobacteria;o__Burkholderiales;f__Burkholderiaceae;g__Cupriavidus;	0.000616	0.000411	0.000205	4.48E-05	9.17E-06	6.64E-05	3.78E-03	3.00	2.35E-02	2.00	8.62E-01	1.50
Lautropia	p__Proteobacteria;c__Betaproteobacteria;o__Burkholderiale	0.027154	0.01292	0.014193	1.99E-04	3.35E-04	2.15E-02	3.61E-02	1.91	1	0.91	4.02E-03	2.10

	s;f__Burkholderiaceae;g__La utropia;												
Ralstonia	p__Proteobacteria;c__Betaproteobacteria;o__Burkholderiales;f__Burkholderiaceae;g__Ralstonia;	6.16E-05	2.05E-05	4.11E-05	2.57E-02	1.78E-01	1	2.97E-01	1.50	5.38E-01	0.50	1	3.00
Comamonadaceae	p__Proteobacteria;c__Betaproteobacteria;o__Burkholderiales;f__Comamonadaceae;	0.00113	0.00076	0.003019	9.00E-08	1.37E-05	2.74E-07	2.77E-03	0.37	6.59E-01	0.25	8.35E-01	1.49
Acidovorax	p__Proteobacteria;c__Betaproteobacteria;o__Burkholderiales;f__Comamonadaceae;g__Acidovorax;	0.000185	0.000164	0.001438	1.23E-06	3.32E-06	1.30E-07	4.22E-03	0.13	4.40E-01	0.11	1	1.13
Comamonas	p__Proteobacteria;c__Betaproteobacteria;o__Burkholderiales;f__Comamonadaceae;g__Comamonas;	8.22E-05	6.16E-05	0.000575	4.37E-07	3.47E-05	9.66E-08	7.44E-02	0.14	6.70E-01	0.11	1	1.33
Malikia	p__Proteobacteria;c__Betaproteobacteria;o__Burkholderiales;f__Comamonadaceae;g__Malikia;	4.11E-05	4.11E-05	5.14E-05	9.41E-01	9.16E-01	1	8.34E-01	0.80	1	0.80	1	1.00
Schlegelella	p__Proteobacteria;c__Betaproteobacteria;o__Burkholderiales;f__Comamonadaceae;g__Schlegelella;	4.11E-05	2.05E-05	4.11E-05	2.64E-01	5.72E-01	1	1	1.00	8.37E-01	0.50	1	2.00

Tepidimonas	p__Proteobacteria;c__Betaproteobacteria;o__Burkholderiales;f__Comamonadaceae;g__Tepidimonas;	6.16E-05	4.11E-05	6.16E-05	3.04E-02	4.66E-01	1.12E-02	4.86E-01	1.00	1	0.67	3.58E-01	1.50
Oxalobacteraceae	p__Proteobacteria;c__Betaproteobacteria;o__Burkholderiales;f__Oxalobacteraceae;	0.000164	0.000103	0.000842	1.09E-04	1.45E-03	1.52E-04	1.16E-01	0.20	1	0.12	1	1.60
Duganella	p__Proteobacteria;c__Betaproteobacteria;o__Burkholderiales;f__Oxalobacteraceae;g__Duganella;	4.11E-05	2.05E-05	0.000472	4.24E-03	1.41E-02	9.42E-02	4.59E-02	0.09	5.54E-01	0.04	1	2.00
Herbaspirillum	p__Proteobacteria;c__Betaproteobacteria;o__Burkholderiales;f__Oxalobacteraceae;g__Herbaspirillum;	4.11E-05	4.11E-05	0.000113	1.75E-02	1.17E-02	1.93E-06	1	0.36	4.78E-01	0.36	9.91E-01	1.00
Janthinobacterium	p__Proteobacteria;c__Betaproteobacteria;o__Burkholderiales;f__Oxalobacteraceae;g__Janthinobacterium;	4.11E-05	4.11E-05	0.000411	2.12E-04	3.48E-04	4.87E-01	6.13E-01	0.10	6.01E-01	0.10	1	1.00
Massilia	p__Proteobacteria;c__Betaproteobacteria;o__Burkholderiales;f__Oxalobacteraceae;g__Massilia;	0.000123	8.22E-05	9.24E-05	1.75E-02	1.14E-02	5.73E-03	8.89E-01	1.33	1	0.89	1	1.50
Hydrogenophilales	p__Proteobacteria;c__Betaproteobacteria;o__Hydrogenophilales;	4.11E-05	2.05E-05	4.11E-05	3.03E-01	9.41E-01	1	1	1.00	7.14E-01	0.50	7.91E-01	2.00

Hydrogenophilaceae	p__Proteobacteria;c__Betaproteobacteria;o__Hydrogenophilales;f__Hydrogenophilaceae;	4.11E-05	2.05E-05	4.11E-05	2.99E-01	9.01E-01	1	1	1.00	8.27E-01	0.50	7.72E-01	2.00
Hydrogenophilus	p__Proteobacteria;c__Betaproteobacteria;o__Hydrogenophilales;f__Hydrogenophilaceae;g__Hydrogenophilus;	4.11E-05	2.05E-05	4.11E-05	1.58E-01	7.19E-01	1	1	1.00	6.05E-01	0.50	8.63E-01	2.00
Methylophilales	p__Proteobacteria;c__Betaproteobacteria;o__Methylophilales;	4.11E-05	3.08E-05	4.11E-05	3.91E-01	2.67E-01	1	1	1.00	1	0.75	9.87E-01	1.33
Methylophilaceae	p__Proteobacteria;c__Betaproteobacteria;o__Methylophilales;f__Methylophilaceae;	4.11E-05	3.08E-05	4.11E-05	3.89E-01	2.83E-01	1	1	1.00	1	0.75	1	1.33
Neisseriales	p__Proteobacteria;c__Betaproteobacteria;o__Neisseriales;	0.119172	0.091833	0.102986	5.40E-01	8.59E-01	1	1	1.16	7.43E-01	0.89	3.18E-01	1.30
Neisseriaceae	p__Proteobacteria;c__Betaproteobacteria;o__Neisseriales;f__Neisseriaceae;	0.119172	0.091833	0.102986	5.57E-01	8.31E-01	2.90E-01	1	1.16	8.35E-01	0.89	2.82E-01	1.30
Alysiella	p__Proteobacteria;c__Betaproteobacteria;o__Neisseriales;f__Neisseriaceae;g__Alysiella;	8.22E-05	6.16E-05	0.000103	5.60E-02	3.48E-01	1	8.89E-01	0.80	5.54E-01	0.60	7.41E-01	1.33
Eikenella	p__Proteobacteria;c__Betaproteobacteria;o__Neisseriales;f__Neisseriaceae;g__Eikenella;	6.16E-05	0.000123	8.22E-05	6.64E-03	6.75E-02	8.99E-04	2.53E-01	0.75	7.54E-01	1.50	3.59E-02	0.50

Kingella	p__Proteobacteria;c__Betaproteobacteria;o__Neisseriales;f__Neisseriaceae;g__Kingella;	0.000185	0.000185	8.22E-05	4.61E-05	1.03E-03	1.36E-05	1.74E-04	2.25	1.32E-01	2.25	1	1.00
Neisseria	p__Proteobacteria;c__Betaproteobacteria;o__Neisseriales;f__Neisseriaceae;g__Neisseria;	0.114099	0.08904	0.094308	4.50E-01	5.08E-01	1	9.65E-01	1.21	6.01E-01	0.94	2.49E-01	1.28
Simonsiella	p__Proteobacteria;c__Betaproteobacteria;o__Neisseriales;f__Neisseriaceae;g__Simonsiella;	4.11E-05	2.05E-05	0.000144	3.75E-05	9.21E-04	5.30E-04	5.40E-02	0.29	4.88E-01	0.14	4.08E-01	2.00
Nitrosomonadales	p__Proteobacteria;c__Betaproteobacteria;o__Nitrosomonadales;	6.16E-05	2.05E-05	4.11E-05	5.35E-02	6.49E-01	1	6.28E-01	1.50	1	0.50	1	3.00
Nitrosomonadaceae	p__Proteobacteria;c__Betaproteobacteria;o__Nitrosomonadales;f__Nitrosomonadaceae;	6.16E-05	2.05E-05	4.11E-05	5.62E-02	6.64E-01	1	6.17E-01	1.50	1	0.50	1	3.00
unidentified Nitrosomonadaceae	p__Proteobacteria;c__Betaproteobacteria;o__Nitrosomonadales;f__Nitrosomonadaceae;g__unidentified_Nitrosomonadaceae;	4.11E-05	0.000164	3.08E-05	7.40E-01	9.68E-01	0	1	1.33	NA	5.34	NA	0.25
Rhodocyclales	p__Proteobacteria;c__Betaproteobacteria;o__Rhodocyclales;	0.000123	4.11E-05	8.22E-05	1.46E-04	5.15E-02	7.97E-02	6.40E-01	1.50	7.43E-01	0.50	1.44E-01	3.00

Rhodocyclaceae	p__Proteobacteria;c__Betaproteobacteria;o__Rhodocyclales;f__Rhodocyclaceae;	0.000123	4.11E-05	8.22E-05	1.37E-04	4.61E-02	1.35E-01	6.17E-01	1.50	8.14E-01	0.50	1.55E-01	3.00
Methyloversatilis	p__Proteobacteria;c__Betaproteobacteria;o__Rhodocyclales;f__Rhodocyclaceae;g__Methyloversatilis;	6.16E-05	4.11E-05	2.05E-05	4.83E-04	3.04E-04	6.76E-04	2.87E-02	3.00	9.47E-01	2.00	1	1.50
Propionivibrio	p__Proteobacteria;c__Betaproteobacteria;o__Rhodocyclales;f__Rhodocyclaceae;g__Propionivibrio;	6.16E-05	4.11E-05	8.22E-05	1.79E-02	8.91E-01	3.92E-01	9.63E-01	0.75	7.68E-01	0.50	3.59E-02	1.50
Thauera	p__Proteobacteria;c__Betaproteobacteria;o__Rhodocyclales;f__Rhodocyclaceae;g__Thauera;	2.05E-05	4.11E-05	4.11E-05	7.80E-01	5.94E-01	1	9.45E-01	0.50	1	1.00	1	0.50
Zoogloea	p__Proteobacteria;c__Betaproteobacteria;o__Rhodocyclales;f__Rhodocyclaceae;g__Zoogloea;	2.05E-05	0.016473	2.05E-05	2.65E-01	7.09E-01	0	1	1.00	NA	803.56	NA	0.00
SC-I-84	p__Proteobacteria;c__Betaproteobacteria;o__SC-I-84;	6.16E-05	2.05E-05	6.16E-05	3.03E-01	9.95E-01	1	1	1.00	1	0.33	1	3.00
TRA3-20	p__Proteobacteria;c__Betaproteobacteria;o__TRA3-20;	4.11E-05	2.05E-05	6.16E-05	3.03E-01	9.80E-01	1	1	0.67	1	0.33	1	2.00
Deltaproteobacteria	p__Proteobacteria;c__Deltaproteobacteria;	0.000318	0.000103	0.000164	1.91E-07	1.18E-05	2.02E-02	7.48E-03	1.94	1	0.63	4.41E-02	3.10

Bdellovibrionales	p__Proteobacteria;c__Deltaproteobacteria;o__Bdellovibrionales;	4.11E-05	4.11E-05	2.05E-05	9.59E-01	9.91E-01	9.54E-01	1	2.00	1	2.00	1	1.00
Desulfobacterales	p__Proteobacteria;c__Deltaproteobacteria;o__Desulfobacterales;	0.000144	6.16E-05	6.16E-05	1.65E-06	2.33E-06	1.92E-03	1.12E-04	2.33	1	1.00	1.24E-01	2.33
Desulfobulbaceae	p__Proteobacteria;c__Deltaproteobacteria;o__Desulfobacterales;f__Desulfobulbaceae;	0.000123	6.16E-05	6.16E-05	1.57E-06	1.56E-06	4.34E-02	2.02E-06	2.00	8.43E-01	1.00	1.39E-01	2.00
Desulfobulbus	p__Proteobacteria;c__Deltaproteobacteria;o__Desulfobacterales;f__Desulfobulbaceae;g__Desulfobulbus;	0.000123	6.16E-05	6.16E-05	1.45E-06	1.29E-06	9.65E-03	4.23E-04	2.00	7.54E-01	1.00	3.13E-01	2.00
Nitrospinaceae	p__Proteobacteria;c__Deltaproteobacteria;o__Desulfobacterales;f__Nitrospinaceae;	4.11E-05	2.05E-05	4.11E-05	5.90E-01	8.31E-01	0	1	1.00	NA	0.50	NA	2.00
Desulfovibrionales	p__Proteobacteria;c__Deltaproteobacteria;o__Desulfovibrionales;	8.22E-05	4.11E-05	6.16E-05	7.67E-03	2.05E-01	1	2.11E-01	1.33	7.14E-01	0.67	8.82E-01	2.00
Desulfovibrionaceae	p__Proteobacteria;c__Deltaproteobacteria;o__Desulfovibrionales;f__Desulfovibrionaceae;	8.22E-05	4.11E-05	6.16E-05	8.57E-03	5.70E-01	1	1	1.33	7.76E-01	0.67	7.92E-01	2.00
Desulfovibrio	p__Proteobacteria;c__Deltaproteobacteria;o__Desulfovibri	6.16E-05	4.11E-05	6.16E-05	7.26E-02	8.02E-01	1	1	1.00	7.63E-01	0.67	1	1.50

	onales;f__Desulfovibrionaceae;g__Desulfovibrio;												
Desulfuromonadales	p__Proteobacteria;c__Deltaproteobacteria;o__Desulfuromonadales;	3.08E-05	4.11E-05	4.11E-05	8.08E-01	7.24E-01	0	1	0.75	1	1.00	8.20E-01	0.75
GR-WP33-30	p__Proteobacteria;c__Deltaproteobacteria;o__GR-WP33-30;	4.11E-05	4.11E-05	4.11E-05	2.21E-01	8.80E-01	1	9.19E-01	1.00	1	1.00	6.19E-01	1.00
Myxococcales	p__Proteobacteria;c__Deltaproteobacteria;o__Myxococcales;	6.16E-05	4.11E-05	4.11E-05	4.47E-01	3.65E-01	8.67E-01	6.16E-01	1.50	8.87E-01	1.00	1	1.50
BIrii41	p__Proteobacteria;c__Deltaproteobacteria;o__Myxococcales;f__BIrii41;	2.05E-05	2.05E-05	0.000164	4.87E-02	6.18E-02	0	8.78E-01	0.12	NA	0.12	NA	1.00
Cystobacteraceae	p__Proteobacteria;c__Deltaproteobacteria;o__Myxococcales;f__Cystobacteraceae;	8.22E-05	4.11E-05	0.000103	8.52E-01	8.99E-01	0	1	0.80	NA	0.40	NA	2.00
Anaeromyxobacter	p__Proteobacteria;c__Deltaproteobacteria;o__Myxococcales;f__Cystobacteraceae;g__Anaeromyxobacter;	8.22E-05	4.11E-05	8.22E-05	8.52E-01	8.91E-01	0	1	1.00	NA	0.50	NA	2.00
Haliangiaceae	p__Proteobacteria;c__Deltaproteobacteria;o__Myxococcales;f__Haliangiaceae;	5.14E-05	2.05E-05	4.11E-05	5.81E-01	8.33E-01	0	1	1.25	NA	0.50	NA	2.50
Haliangium	p__Proteobacteria;c__Deltaproteobacteria;o__Myxococcale	5.14E-05	2.05E-05	4.11E-05	5.78E-01	8.19E-01	0	9.65E-01	1.25	NA	0.50	NA	2.50

	s;f__Haliangiaceae;g__Haliangium;												
Polyangiaceae	p__Proteobacteria;c__Deltaproteobacteria;o__Myxococcales;f__Polyangiaceae;	4.11E-05	3.08E-05	4.11E-05	8.31E-01	8.32E-01	1	1	1.00	1	0.75	1	1.33
Sorangium	p__Proteobacteria;c__Deltaproteobacteria;o__Myxococcales;f__Polyangiaceae;g__Sorangium;	6.16E-05	3.08E-05	4.11E-05	7.80E-01	9.78E-01	1	1	1.50	1	0.75	1	2.00
Sandaracinaceae	p__Proteobacteria;c__Deltaproteobacteria;o__Myxococcales;f__Sandaracinaceae;	4.11E-05	2.05E-05	4.11E-05	5.81E-01	7.89E-01	0	1	1.00	NA	0.50	NA	2.00
Oligoflexales	p__Proteobacteria;c__Deltaproteobacteria;o__Oligoflexales;	6.16E-05	0.000308	4.11E-05	3.17E-01	7.29E-01	0	9.50E-01	1.50	NA	7.50	NA	0.20
Syntrophobacterales	p__Proteobacteria;c__Deltaproteobacteria;o__Syntrophobacterales;	4.11E-05	4.11E-05	4.11E-05	8.08E-01	7.18E-01	0	1	1.00	NA	1.00	NA	1.00
Syntrophobacteraceae	p__Proteobacteria;c__Deltaproteobacteria;o__Syntrophobacterales;f__Syntrophobacteraceae;	4.11E-05	0	4.11E-05	7.62E-01	8.28E-01	0	1	1.00	NA	NA	NA	NA
Epsilonproteobacteria	p__Proteobacteria;c__Epsilonproteobacteria;	0.005731	0.006141	0.003297	8.41E-04	8.03E-04	2.56E-03	2.52E-02	1.74	1.73E-01	1.86	1	0.93

Campylobacterales	p__Proteobacteria;c__Epsilonproteobacteria;o__Campylobacterales;	0.005731	0.006141	0.003297	8.89E-04	9.46E-04	3.17E-03	2.53E-02	1.74	1.17E-01	1.86	1	0.93
Campylobacteraceae	p__Proteobacteria;c__Epsilonproteobacteria;o__Campylobacterales;f__Campylobacteraceae;	0.005731	0.006141	0.003245	8.32E-04	9.85E-04	1.49E-02	1.40E-02	1.77	2.11E-01	1.89	1	0.93
Campylobacter	p__Proteobacteria;c__Epsilonproteobacteria;o__Campylobacterales;f__Campylobacteraceae;g__Campylobacter;	0.005731	0.006141	0.003245	8.46E-04	1.01E-03	8.90E-03	3.61E-02	1.77	2.01E-01	1.89	1	0.93
Helicobacteraceae	p__Proteobacteria;c__Epsilonproteobacteria;o__Campylobacterales;f__Helicobacteraceae;	4.11E-05	3.08E-05	5.14E-05	3.27E-01	6.98E-01	1	1	0.80	9.72E-01	0.60	1	1.33
Helicobacter	p__Proteobacteria;c__Epsilonproteobacteria;o__Campylobacterales;f__Helicobacteraceae;g__Helicobacter;	4.11E-05	2.05E-05	6.16E-05	1.17E-01	2.25E-01	0	9.61E-01	0.67	NA	0.33	NA	2.00
Wolinella	p__Proteobacteria;c__Epsilonproteobacteria;o__Campylobacterales;f__Helicobacteraceae;g__Wolinella;	4.11E-05	4.11E-05	4.11E-05	3.62E-01	2.55E-01	1	1	1.00	1	1.00	1	1.00
Gammaproteobacteria	p__Proteobacteria;c__Gammaproteobacteria;	0.133118	0.157396	0.254971	4.78E-06	1.19E-06	8.40E-04	8.09E-02	0.52	1.85E-01	0.62	8.89E-01	0.85

Aeromonadales	p__Proteobacteria;c__Gammaproteobacteria;o__Aeromonadales;	0.000103	0.000113	6.16E-05	4.70E-03	7.83E-03	5.12E-01	2.11E-01	1.67	6.75E-02	1.83	1	0.91
Aeromonadaceae	p__Proteobacteria;c__Gammaproteobacteria;o__Aeromonadales;f__Aeromonadaceae;	6.16E-05	0.000113	4.11E-05	2.96E-03	4.01E-01	2.15E-01	7.46E-01	1.50	1.67E-01	2.75	5.37E-01	0.55
Aeromonas	p__Proteobacteria;c__Gammaproteobacteria;o__Aeromonadales;f__Aeromonadaceae;g__Aeromonas;	6.16E-05	0.000113	4.11E-05	4.24E-03	4.66E-01	1.79E-01	9.80E-01	1.50	6.48E-01	2.75	6.12E-01	0.55
Succinivibrionaceae	p__Proteobacteria;c__Gammaproteobacteria;o__Aeromonadales;f__Succinivibrionaceae;	6.16E-05	0	3.08E-05	1.74E-02	3.08E-02	0	1	2.00	NA	NA	NA	NA
Succinivibrionaceae UCG-002	p__Proteobacteria;c__Gammaproteobacteria;o__Aeromonadales;f__Succinivibrionaceae;g__Succinivibrionaceae_UCG-002;	0.000472	0	2.05E-05	2.57E-04	6.10E-04	0	3.95E-01	23.04	NA	NA	NA	NA
Alteromonadales	p__Proteobacteria;c__Gammaproteobacteria;o__Alteromonadales;	0.001068	0.000195	0.000616	1.73E-05	8.85E-02	3.64E-09	2.11E-01	1.73	1.17E-01	0.32	7.53E-04	5.47
Shewanellaceae	p__Proteobacteria;c__Gammaproteobacteria;o__Alteromonadales;f__Shewanellaceae;	0.001048	0.000195	0.000616	8.74E-06	6.18E-02	4.52E-09	6.45E-02	1.70	2.69E-01	0.32	5.68E-04	5.37
Shewanella	p__Proteobacteria;c__Gammaproteobacteria;o__Alteromon	0.001048	0.000195	0.000616	9.09E-06	4.90E-02	1.68E-08	7.28E-02	1.70	3.07E-01	0.32	5.48E-04	5.37

	adales;f__Shewanellaceae;g__Shewanella;												
Cardiobacteriales	p__Proteobacteria;c__Gammaproteobacteria;o__Cardiobacteriales;	0.000965	0.000719	0.001171	1.18E-01	2.33E-01	1	1	0.82	2.62E-01	0.61	7.91E-01	1.34
Cardiobacteriaceae	p__Proteobacteria;c__Gammaproteobacteria;o__Cardiobacteriales;f__Cardiobacteriaceae;	0.000965	0.000719	0.001171	1.09E-01	2.35E-01	1	1	0.82	4.22E-01	0.61	7.92E-01	1.34
Cardiobacterium	p__Proteobacteria;c__Gammaproteobacteria;o__Cardiobacteriales;f__Cardiobacteriaceae;g__Cardiobacterium;	0.000945	0.000719	0.001171	1.02E-01	1.87E-01	1	1	0.81	4.48E-01	0.61	9.27E-01	1.31
Cellvibrionales	p__Proteobacteria;c__Gammaproteobacteria;o__Cellvibrionales;	3.08E-05	6.16E-05	4.11E-05	2.21E-01	7.29E-01	1	9.29E-01	0.75	6.31E-01	1.50	7.91E-01	0.50
Chromatiales	p__Proteobacteria;c__Gammaproteobacteria;o__Chromatiales;	4.11E-05	3.08E-05	0.000144	3.47E-03	2.97E-03	7.75E-06	1	0.29	2.00E-01	0.21	1	1.33
Chromatiaceae	p__Proteobacteria;c__Gammaproteobacteria;o__Chromatiales;f__Chromatiaceae;	4.11E-05	2.05E-05	0.000144	1.20E-02	1.49E-02	1.67E-05	3.46E-01	0.29	3.93E-01	0.14	1	2.00
Rheinheimera	p__Proteobacteria;c__Gammaproteobacteria;o__Chromatiales;f__Chromatiaceae;g__Rheinheimera;	4.11E-05	2.05E-05	0.000144	2.46E-02	2.93E-02	1.92E-05	9.20E-02	0.29	3.62E-01	0.14	1	2.00

Enterobacteriales	p__Proteobacteria;c__Gammaproteobacteria;o__Enterobacteriales;	0.007702	0.005648	0.018609	3.41E-05	1.52E-03	2.47E-03	4.72E-03	0.41	7.16E-02	0.30	4.90E-01	1.36
Enterobacteriaceae	p__Proteobacteria;c__Gammaproteobacteria;o__Enterobacteriales;f__Enterobacteriaceae;	0.007702	0.005648	0.018609	3.31E-05	1.57E-03	1.36E-03	1.27E-02	0.41	2.79E-02	0.30	4.18E-01	1.36
Arsenophonus	p__Proteobacteria;c__Gammaproteobacteria;o__Enterobacteriales;f__Enterobacteriaceae;g__Arsenophonus;	6.16E-05	0	4.11E-05	2.99E-01	3.49E-01	0	1	1.50	NA	NA	NA	NA
Buchnera	p__Proteobacteria;c__Gammaproteobacteria;o__Enterobacteriales;f__Enterobacteriaceae;g__Buchnera;	7.19E-05	2.05E-05	4.11E-05	2.89E-02	6.75E-02	2.00E-06	5.57E-02	1.75	1	0.50	1	3.51
Escherichia-Shigella	p__Proteobacteria;c__Gammaproteobacteria;o__Enterobacteriales;f__Enterobacteriaceae;g__Escherichia-Shigella;	0.000606	0.00037	0.000452	8.00E-01	8.86E-01	1	1	1.34	1	0.82	1	1.64
Yersinia	p__Proteobacteria;c__Gammaproteobacteria;o__Enterobacteriales;f__Enterobacteriaceae;g__Yersinia;	0.000267	8.22E-05	0.001006	5.60E-08	1.85E-02	1.81E-09	1.61E-01	0.27	9.81E-02	0.08	9.14E-01	3.25
Legionellales	p__Proteobacteria;c__Gammaproteobacteria;o__Legionellales;	4.11E-05	4.11E-05	4.11E-05	9.82E-01	9.61E-01	1	1	1.00	1	1.00	8.20E-01	1.00

Coxiellaceae	p__Proteobacteria;c__Gammaproteobacteria;o__Legionellales;f__Coxiellaceae;	4.11E-05	4.11E-05	4.11E-05	8.15E-01	6.98E-01	1	1	1.00	1	1.00	1	1.00
Aquicella	p__Proteobacteria;c__Gammaproteobacteria;o__Legionellales;f__Coxiellaceae;g__Aquicella;	4.11E-05	4.11E-05	3.08E-05	9.03E-01	7.55E-01	1	9.54E-01	1.33	1	1.33	1	1.00
Oceanospirillales	p__Proteobacteria;c__Gammaproteobacteria;o__Oceanospirillales;	0.001725	0.00039	0.001109	3.72E-06	7.01E-02	3.05E-06	2.98E-01	1.56	4.15E-01	0.35	2.40E-03	4.42
Halomonadaceae	p__Proteobacteria;c__Gammaproteobacteria;o__Oceanospirillales;f__Halomonadaceae;	0.001725	0.000421	0.001109	3.39E-06	6.18E-02	1.94E-06	2.65E-01	1.56	7.17E-01	0.38	6.35E-03	4.10
Halomonas	p__Proteobacteria;c__Gammaproteobacteria;o__Oceanospirillales;f__Halomonadaceae;g__Halomonas;	0.001725	0.000421	0.001109	3.39E-06	5.02E-02	4.95E-06	2.05E-01	1.56	5.83E-01	0.38	4.06E-03	4.10
Oceanospirillaceae	p__Proteobacteria;c__Gammaproteobacteria;o__Oceanospirillales;f__Oceanospirillaceae;	4.11E-05	0.000883	3.08E-05	1.47E-01	3.70E-01	0	7.23E-01	1.33	NA	28.68	NA	0.05
Pasteurellales	p__Proteobacteria;c__Gammaproteobacteria;o__Pasteurellales;	0.040628	0.058025	0.048936	3.03E-01	5.17E-01	6.98E-01	2.11E-01	0.83	7.14E-01	1.19	3.18E-01	0.70
Pasteurellaceae	p__Proteobacteria;c__Gammaproteobacteria;o__Pasteurellales;f__Pasteurellaceae;	0.040628	0.058025	0.048936	2.99E-01	5.11E-01	1	1.91E-01	0.83	7.76E-01	1.19	2.19E-01	0.70

Actinobacillus	p__Proteobacteria;c__Gammaproteobacteria;o__Pasteurellales;f__Pasteurellaceae;g__Actinobacillus;	0.000965	0.001376	0.001931	2.39E-02	1.17E-02	6.39E-01	4.59E-02	0.50	1	0.71	5.26E-01	0.70
Aggregatibacter	p__Proteobacteria;c__Gammaproteobacteria;o__Pasteurellales;f__Pasteurellaceae;g__Aggregatibacter;	0.001746	0.001808	0.002834	4.10E-03	1.48E-03	2.50E-02	3.28E-02	0.62	1	0.64	1	0.97
Haemophilus	p__Proteobacteria;c__Gammaproteobacteria;o__Pasteurellales;f__Pasteurellaceae;g__Haemophilus;	0.000205	0.000349	0.000308	1.14E-02	1.49E-02	1.92E-01	3.86E-02	0.67	1	1.13	9.58E-01	0.59
Pseudomonadales	p__Proteobacteria;c__Gammaproteobacteria;o__Pseudomonadales;	0.035493	0.048063	0.103479	9.16E-04	2.86E-04	1.12E-01	2.11E-01	0.34	1.32E-01	0.46	8.03E-01	0.74
Moraxellaceae	p__Proteobacteria;c__Gammaproteobacteria;o__Pseudomonadales;f__Moraxellaceae;	0.022963	0.024422	0.043924	1.78E-02	1.30E-02	1	4.80E-01	0.52	7.76E-01	0.56	1	0.94
Acinetobacter	p__Proteobacteria;c__Gammaproteobacteria;o__Pseudomonadales;f__Moraxellaceae;g__Acinetobacter;	0.019533	0.021793	0.033839	1.15E-01	5.41E-02	1	1.55E-01	0.58	7.54E-01	0.64	1	0.90
Alkanindiges	p__Proteobacteria;c__Gammaproteobacteria;o__Pseudomonadales;f__Moraxellaceae;g__Alkanindiges;	0.000164	0.000329	4.11E-05	6.47E-05	4.47E-02	3.37E-23	2.32E-01	4.00	2.53E-03	8.00	3.13E-01	0.50

Moraxella	p__Proteobacteria;c__Gammaproteobacteria;o__Pseudomonadales;f__Moraxellaceae;g__Moraxella;	0.001664	0.000822	0.00266	2.54E-04	6.75E-02	4.64E-04	3.07E-01	0.63	3.90E-01	0.31	7.41E-01	2.03
Psychrobacter	p__Proteobacteria;c__Gammaproteobacteria;o__Pseudomonadales;f__Moraxellaceae;g__Psychrobacter;	0.000164	0.000431	0.000134	3.88E-02	3.67E-01	2.39E-06	9.65E-01	1.23	1	3.23	2.41E-01	0.38
Pseudomonadaceae	p__Proteobacteria;c__Gammaproteobacteria;o__Pseudomonadales;f__Pseudomonadaceae;	0.001869	0.015446	0.030368	3.56E-11	5.41E-12	2.15E-15	7.58E-06	0.06	6.59E-01	0.51	6.62E-02	0.12
Pseudomonas	p__Proteobacteria;c__Gammaproteobacteria;o__Pseudomonadales;f__Pseudomonadaceae;g__Pseudomonas;	0.001869	0.015446	0.030368	5.88E-11	8.94E-12	3.09E-15	1.91E-05	0.06	5.69E-01	0.51	6.75E-02	0.12
Thiotrichales	p__Proteobacteria;c__Gammaproteobacteria;o__Thiotrichales;	4.11E-05	0	6.16E-05	2.03E-01	2.66E-01	0	4.94E-01	0.67	NA	NA	NA	NA
Piscirickettsiaceae	p__Proteobacteria;c__Gammaproteobacteria;o__Thiotrichales;f__Piscirickettsiaceae;	4.11E-05	0	6.16E-05	1.81E-01	2.49E-01	0	5.71E-01	0.67	NA	NA	NA	NA
Methylophaga	p__Proteobacteria;c__Gammaproteobacteria;o__Thiotrichales;f__Piscirickettsiaceae;g__Methylophaga;	4.11E-05	0	6.16E-05	1.59E-01	1.96E-01	0	3.99E-01	0.67	NA	NA	NA	NA

Vibrionales	p__Proteobacteria;c__Gammaproteobacteria;o__Vibrionales;	6.16E-05	2.05E-05	4.11E-05	4.15E-02	2.67E-01	2.28E-04	5.14E-01	1.50	NA	0.50	NA	3.00
Vibrionaceae	p__Proteobacteria;c__Gammaproteobacteria;o__Vibrionales;f__Vibrionaceae;	6.16E-05	2.05E-05	4.11E-05	4.35E-02	2.83E-01	4.61E-06	4.96E-01	1.50	NA	0.50	NA	3.00
Vibrio	p__Proteobacteria;c__Gammaproteobacteria;o__Vibrionales;f__Vibrionaceae;g__Vibrio;	6.16E-05	2.05E-05	4.11E-05	1.14E-01	3.22E-01	0	3.87E-01	1.50	NA	0.50	NA	3.00
Xanthomonadales	p__Proteobacteria;c__Gammaproteobacteria;o__Xanthomonadales;	0.003533	0.002342	0.005125	1.70E-04	1.16E-01	1.71E-01	3.17E-01	0.69	1.17E-01	0.46	8.71E-01	1.51
Nevskiaceae	p__Proteobacteria;c__Gammaproteobacteria;o__Xanthomonadales;f__Nevskiaceae;	4.11E-05	2.05E-05	4.11E-05	5.82E-02	3.33E-01	1	1	1.00	9.43E-01	0.50	3.17E-01	2.00
Nevskia	p__Proteobacteria;c__Gammaproteobacteria;o__Xanthomonadales;f__Nevskiaceae;g__Nevskia;	4.11E-05	2.05E-05	3.08E-05	1.82E-01	2.49E-01	0	1	1.33	NA	0.67	NA	2.00
unidentified Xanthomonadales	p__Proteobacteria;c__Gammaproteobacteria;o__Xanthomonadales;f__unidentified_Xanthomonadales;	6.16E-05	4.11E-05	5.14E-05	1.11E-01	4.78E-01	1	7.46E-01	1.20	1	0.80	7.92E-01	1.50
Acidibacter	p__Proteobacteria;c__Gammaproteobacteria;o__Xanthomo	6.16E-05	4.11E-05	4.11E-05	2.55E-01	4.44E-01	1	5.60E-01	1.50	1	1.00	1	1.50

	nadales;f__unidentified_Xanthomonadales;g__Acidibacter;												
Steroidobacter	p__Proteobacteria;c__Gammaproteobacteria;o__Xanthomonadales;f__unidentified_Xanthomonadales;g__Steroidobacter;	4.11E-05	4.11E-05	4.11E-05	8.87E-01	9.29E-01	0	1	1.00	1	1.00	9.91E-01	1.00
Xanthomonadaceae	p__Proteobacteria;c__Gammaproteobacteria;o__Xanthomonadales;f__Xanthomonadaceae;	0.003286	0.002177	0.004909	2.37E-04	9.25E-02	6.37E-02	5.64E-01	0.67	2.69E-01	0.44	7.92E-01	1.51
Arenimonas	p__Proteobacteria;c__Gammaproteobacteria;o__Xanthomonadales;f__Xanthomonadaceae;g__Arenimonas;	4.11E-05	2.05E-05	4.11E-05	7.80E-01	9.87E-01	1	1	1.00	1	0.50	1	2.00
Pseudoxanthomonas	p__Proteobacteria;c__Gammaproteobacteria;o__Xanthomonadales;f__Xanthomonadaceae;g__Pseudoxanthomonas;	4.11E-05	4.11E-05	0.000462	8.03E-02	4.10E-02	1.10E-02	5.51E-01	0.09	1	0.09	1	1.00
Rhodanobacter	p__Proteobacteria;c__Gammaproteobacteria;o__Xanthomonadales;f__Xanthomonadaceae;g__Rhodanobacter;	2.05E-05	9.24E-05	0.000164	7.26E-02	3.36E-02	4.50E-03	9.65E-01	0.12	1	0.56	1	0.22
Silanimonas	p__Proteobacteria;c__Gammaproteobacteria;o__Xanthomo	0.002033	0.000986	0.000616	6.32E-08	1.20E-08	4.28E-07	1.27E-04	3.30	4.88E-01	1.60	2.85E-01	2.06

	nadales;f__Xanthomonadaceae;g__Silanimonas;												
Stenotrophomonas	p__Proteobacteria;c__Gammaproteobacteria;o__Xanthomonadales;f__Xanthomonadaceae;g__Stenotrophomonas;	0.000288	0.000431	0.003615	1.35E-07	5.15E-07	1.40E-07	1.87E-02	0.08	1.67E-01	0.12	9.84E-01	0.67
Saccharibacteria	p__Saccharibacteria;	0.013823	0.011584	0.00913	4.38E-01	2.56E-01	1	5.30E-01	1.51	1	1.27	1	1.19
unidentified Saccharibacteria	p__Saccharibacteria;c__unidentified_Saccharibacteria;	0.012283	0.009777	0.007836	4.73E-01	2.74E-01	1	8.08E-01	1.57	1	1.25	1	1.26
unidentified Saccharibacteria	p__Saccharibacteria;c__unidentified_Saccharibacteria;o__unidentified_Saccharibacteria;	0.012283	0.009777	0.007836	4.23E-01	2.66E-01	1	7.08E-01	1.57	1	1.25	1	1.26
unidentified Saccharibacteria	p__Saccharibacteria;c__unidentified_Saccharibacteria;o__unidentified_Saccharibacteria;f__unidentified_Saccharibacteria;	0.012283	0.009777	0.007836	4.25E-01	2.80E-01	1	6.86E-01	1.57	1	1.25	1	1.26
Candidatus Saccharimonas	p__Saccharibacteria;c__unidentified_Saccharibacteria;o__unidentified_Saccharibacteria;f__unidentified_Saccharibacteria;g__Candidatus_Saccharimonas;	0.002075	0.002033	0.002177	8.54E-01	7.01E-01	1	1	0.95	1	0.93	1	1.02
unidentified Saccharibacteria	p__Saccharibacteria;c__unidentified_Saccharibacteria;o__unidentified_Saccharibacteria;f	0.009222	0.007107	0.005844	3.92E-01	2.19E-01	1	5.21E-01	1.58	1	1.22	1	1.30

	__unidentified_Saccharibacteria;g__unidentified_Saccharibacteria;												
Spirochaetes	p__Spirochaetes;	0.000863	0.000144	0.000246	3.33E-13	2.60E-07	3.42E-12	2.79E-03	3.50	1	0.58	2.42E-02	6.00
unidentified Spirochaetes	p__Spirochaetes;c__unidentified_Spirochaetes;	0.000863	0.000144	0.000246	7.05E-13	2.24E-07	2.62E-11	7.48E-03	3.50	1	0.58	4.83E-02	6.00
Spirochaetales	p__Spirochaetes;c__unidentified_Spirochaetes;o__Spirochaetales;	0.000863	0.000144	0.000246	1.33E-12	3.46E-07	4.79E-11	2.97E-03	3.50	1	0.58	3.05E-02	6.00
Spirochaetaceae	p__Spirochaetes;c__unidentified_Spirochaetes;o__Spirochaetales;f__Spirochaetaceae;	0.000873	0.000144	0.000246	4.23E-13	2.37E-07	8.53E-11	4.28E-03	3.54	1	0.58	2.02E-02	6.07
Sphaerochaeta	p__Spirochaetes;c__unidentified_Spirochaetes;o__Spirochaetales;f__Spirochaetaceae;g__Sphaerochaeta;	6.16E-05	4.11E-05	2.05E-05	1.01E-01	4.66E-02	5.62E-10	6.26E-01	3.00	9.29E-01	2.00	1	1.50
Treponema 2	p__Spirochaetes;c__unidentified_Spirochaetes;o__Spirochaetales;f__Spirochaetaceae;g__Treponema_2;	0.000832	0.000144	0.000226	2.01E-12	3.18E-07	1.49E-10	1.07E-03	3.68	1	0.64	4.02E-03	5.79
Synergistetes	p__Synergistetes;	0.000832	0.00039	0.000442	9.87E-05	1.41E-03	3.73E-01	6.55E-02	1.88	1	0.88	1.33E-01	2.13
Synergistia	p__Synergistetes;c__Synergistia;	0.000832	0.00039	0.000442	1.22E-04	1.64E-03	5.52E-01	1.95E-01	1.88	1	0.88	3.19E-01	2.13
Synergistales	p__Synergistetes;c__Synergistia;o__Synergistales;	0.000832	0.00039	0.000442	1.46E-04	1.89E-03	7.87E-01	2.11E-01	1.88	1	0.88	3.18E-01	2.13

Synergistaceae	p__Synergistetes;c__Synergistia;o__Synergistales;f__Synergistaceae;	0.000832	0.00039	0.000442	1.33E-04	1.99E-03	1	1.22E-01	1.88	1	0.88	2.82E-01	2.13
Fretibacterium	p__Synergistetes;c__Synergistia;o__Synergistales;f__Synergistaceae;g__Fretibacterium;	0.000832	0.00039	0.000442	1.61E-04	2.06E-03	1	1.55E-01	1.88	1	0.88	3.41E-01	2.13
Tenericutes	p__Tenericutes;	0.001849	0.00039	0.001232	1.34E-06	3.87E-02	7.02E-07	7.34E-02	1.50	3.09E-01	0.32	2.85E-03	4.74
Mollicutes	p__Tenericutes;c__Mollicutes;	0.001849	0.00039	0.001232	9.60E-07	4.11E-02	1.96E-05	1.10E-01	1.50	4.94E-01	0.32	2.22E-04	4.74
Acholeplasmatales	p__Tenericutes;c__Mollicutes;o__Acholeplasmatales;	6.16E-05	4.11E-05	6.16E-05	8.62E-02	7.29E-01	1	1	1.00	6.79E-01	0.67	3.58E-01	1.50
Acholeplasmataceae	p__Tenericutes;c__Mollicutes;o__Acholeplasmatales;f__Acholeplasmataceae;	6.16E-05	4.11E-05	6.16E-05	8.11E-02	7.10E-01	1	1	1.00	7.76E-01	0.67	3.36E-01	1.50
Acholeplasma	p__Tenericutes;c__Mollicutes;o__Acholeplasmatales;f__Acholeplasmataceae;g__Acholeplasma;	6.16E-05	4.11E-05	6.16E-05	7.79E-02	6.61E-01	1	1	1.00	6.70E-01	0.67	4.74E-01	1.50
Anaeroplasmatales	p__Tenericutes;c__Mollicutes;o__Anaeroplasmatales;	6.16E-05	0	4.11E-05	1.82E-01	2.34E-01	0	8.03E-01	1.50	NA	NA	NA	NA
Anaeroplasmataceae	p__Tenericutes;c__Mollicutes;o__Anaeroplasmatales;f__Anaeroplasmataceae;	6.16E-05	0	4.11E-05	1.74E-01	2.39E-01	0	8.67E-01	1.50	NA	NA	NA	NA
Anaeroplasma	p__Tenericutes;c__Mollicutes;o__Anaeroplasmatales;f__A	6.16E-05	0	4.11E-05	1.59E-01	1.91E-01	0	6.87E-01	1.50	NA	NA	NA	NA

	naeroplasmataceae;g__Anaeroplasma;												
Mollicutes RF9	p__Tenericutes;c__Mollicutes;o__Mollicutes_RF9;	0.001171	0.000329	0.000739	1.63E-04	2.66E-01	7.95E-03	6.10E-01	1.58	2.26E-01	0.44	7.53E-04	3.56
unidentified Mollicutes RF9	p__Tenericutes;c__Mollicutes;o__Mollicutes_RF9;f__unidentified_Mollicutes_RF9;	0.000205	6.16E-05	0.000164	6.99E-06	1.06E-01	2.76E-02	5.64E-01	1.25	1.39E-02	0.37	2.43E-02	3.33
unidentified Mollicutes RF9	p__Tenericutes;c__Mollicutes;o__Mollicutes_RF9;f__unidentified_Mollicutes_RF9;g__unidentified_Mollicutes_RF9;	0.000205	6.16E-05	0.000164	7.06E-06	8.60E-02	2.53E-02	4.98E-01	1.25	6.95E-03	0.37	6.30E-02	3.33
Mycoplasmatales	p__Tenericutes;c__Mollicutes;o__Mycoplasmatales;	0.000226	4.11E-05	9.24E-05	1.05E-09	9.48E-05	1.45E-06	2.67E-03	2.44	8.87E-01	0.44	7.53E-04	5.50
Mycoplasmataceae	p__Tenericutes;c__Mollicutes;o__Mycoplasmatales;f__Mycoplasmataceae;	0.000226	4.11E-05	9.24E-05	8.58E-10	1.10E-04	5.48E-07	6.52E-04	2.44	9.70E-01	0.44	5.17E-04	5.50
Mycoplasma	p__Tenericutes;c__Mollicutes;o__Mycoplasmatales;f__Mycoplasmataceae;g__Mycoplasma;	0.000226	4.11E-05	9.24E-05	2.31E-09	8.58E-05	3.60E-07	2.06E-03	2.44	9.02E-01	0.44	1.47E-03	5.50
NB1-n	p__Tenericutes;c__Mollicutes;o__NB1-n;	6.16E-05	4.11E-05	4.11E-05	3.28E-02	1.14E-01	1.20E-02	2.11E-01	1.50	8.87E-01	1.00	9.58E-01	1.50
Thermomicrobia	p__Thermomicrobia;	0.000103	2.05E-05	2.05E-05	1.25E-01	9.54E-02	0	8.83E-01	5.01	NA	1.00	NA	5.01

unidentified Thermomicrobia	p__Thermomicrobia;c__unidentified_Thermomicrobia;	0.000103	2.05E-05	2.05E-05	1.49E-01	1.05E-01	0	1	5.01	NA	1.00	NA	5.01
JG30-KF-CM45	p__Thermomicrobia;c__unidentified_Thermomicrobia;o__JG30-KF-CM45;	0.000103	2.05E-05	2.05E-05	1.80E-01	1.51E-01	0	8.71E-01	5.01	NA	1.00	NA	5.01
Verrucomicrobia	p__Verrucomicrobia;	6.16E-05	4.11E-05	4.11E-05	5.24E-01	4.26E-01	1	8.83E-01	1.50	1	1.00	1	1.50
OPB35 soil group	p__Verrucomicrobia;c__OPB35_soil_group;	5.14E-05	8.22E-05	4.11E-05	7.60E-01	6.25E-01	1	9.96E-01	1.25	1	2.00	7.44E-01	0.62
unidentified OPB35 soil group	p__Verrucomicrobia;c__OPB35_soil_group;o__unidentified_OPB35_soil_group;	8.22E-05	2.05E-05	4.11E-05	3.55E-01	7.24E-01	0	7.12E-01	2.00	NA	0.50	NA	4.01
unidentified OPB35 soil group	p__Verrucomicrobia;c__OPB35_soil_group;o__unidentified_OPB35_soil_group;f__unidentified_OPB35_soil_group;	8.22E-05	2.05E-05	4.11E-05	3.58E-01	6.98E-01	0	8.06E-01	2.00	NA	0.50	NA	4.01
Opitutae	p__Verrucomicrobia;c__Opitutae;	6.16E-05	0.00039	4.11E-05	3.74E-01	5.41E-01	0	1	1.50	NA	9.50	NA	0.16
Spartobacteria	p__Verrucomicrobia;c__Spartobacteria;	4.11E-05	4.11E-05	4.11E-05	6.00E-01	9.43E-01	1	1	1.00	1	1.00	1	1.00
Chthoniobacterales	p__Verrucomicrobia;c__Spartobacteria;o__Chthoniobacterales;	4.11E-05	4.11E-05	4.11E-05	5.99E-01	9.61E-01	1	1	1.00	1	1.00	8.82E-01	1.00

Chthoniobacteraceae	p__Verrucomicrobia;c__Spartobacteria;o__Chthoniobacterales;f__Chthoniobacteraceae;	5.14E-05	2.05E-05	2.05E-05	5.24E-01	5.81E-01	0	1	2.50	1	1.00	1	2.50
Chthoniobacter	p__Verrucomicrobia;c__Spartobacteria;o__Chthoniobacterales;f__Chthoniobacteraceae;g__Chthoniobacter;	5.14E-05	2.05E-05	2.05E-05	5.01E-01	5.15E-01	0	1	2.50	1	1.00	1	2.50
DA101 soil group	p__Verrucomicrobia;c__Spartobacteria;o__Chthoniobacterales;f__DA101_soil_group;	4.11E-05	2.05E-05	0.000205	7.49E-02	6.98E-01	1	4.56E-01	0.20	1	0.10	1	2.00
Verrucomicrobiae	p__Verrucomicrobia;c__Verrucomicrobiae;	3.08E-05	6.17E-05	4.11E-05	8.29E-01	8.83E-01	1	9.22E-01	0.75	1	1.50	1	0.50
Verrucomicrobiales	p__Verrucomicrobia;c__Verrucomicrobiae;o__Verrucomicrobiales;	3.08E-05	6.17E-05	4.11E-05	8.56E-01	8.92E-01	1	1	0.75	1	1.50	1	0.50
Verrucomicrobiaceae	p__Verrucomicrobia;c__Verrucomicrobiae;o__Verrucomicrobiales;f__Verrucomicrobiaceae;	3.08E-05	6.17E-05	4.11E-05	8.52E-01	9.18E-01	1	1	0.75	1	1.50	1	0.50

[1]False discovery rate adjusted *q*-values were calculated based on *p*-values estimated by Kruskal-Wallis tests.

[2]False discovery rate adjusted *q*-values were calculated based on *p*-values estimated by Jonckheere-Terpstra tests.

[3]Holm adjusted *q*-values were calculated based on *p*-values estimated by ANCOM-BC, adjusting for age, sex and the education level.

[4]False discovery rate adjusted *q*-values were calculated based on *p*-values estimated by quantile regression, adjusting for age, sex and the education level.

Supplementary Table 2. The correlation network of salivary microbiota with symptoms of two disease statuses.

Symptoms	Taxa	Phylum	r	*p* -value	Comparison
Affective flattening	f| Peptococcaceae	Firmicutes	-0.305	0.021	FES vs HC
Affective flattening	g| Peptococcus	Firmicutes	-0.265	0.046	FES vs HC
Affective flattening	f| Bradyrhizobiaceae	Proteobacteria	-0.302	0.044	FES vs HC
Affective flattening	f| Comamonadaceae	Proteobacteria	-0.283	0.031	FES vs HC
Alogia	f| Peptococcaceae	Firmicutes	-0.370	0.005	FES vs HC
Alogia	g| Peptococcus	Firmicutes	-0.335	0.011	FES vs HC
Anhedonia	f| Micrococcaceae	Actinobacteria	-0.270	0.040	FES vs HC
Anhedonia	g| Rothia	Actinobacteria	-0.272	0.039	FES vs HC
Anhedonia	f| Sphingobacteriaceae	Bacteroidetes	0.327	0.028	FES vs HC
Anhedonia	g| Brevibacillus	Firmicutes	-0.284	0.039	FES vs HC
Anhedonia	g| Eubacterium coprostanoligenes group	Firmicutes	-0.379	0.047	FES vs HC
Anhedonia	g| Paenibacillus	Firmicutes	-0.264	0.045	FES vs HC
Anhedonia	f| Leptotrichiaceae	Fusobacteria	-0.289	0.028	FES vs HC
Anhedonia	g| Leptotrichia	Fusobacteria	-0.273	0.038	FES vs HC
Anhedonia	f| Rhodospirillaceae	Proteobacteria	0.359	0.040	FES vs HC
Attention	c| Thermoleophilia	Actinobacteria	0.335	0.018	FES vs HC
Attention	g| Lactococcus	Firmicutes	0.400	0.026	FES vs HC
Attention	f| Rhodospirillaceae	Proteobacteria	0.555	0.001	FES vs HC
Attention	g| Moraxella	Proteobacteria	-0.347	0.008	FES vs HC
Attention	g| Rhizobium	Proteobacteria	0.362	0.050	FES vs HC
Attention	g| Stenotrophomonas	Proteobacteria	-0.279	0.036	FES vs HC
Attention	o| Rhodospirillales	Proteobacteria	0.387	0.005	FES vs HC
Avolition	c| Thermoleophilia	Actinobacteria	0.304	0.032	FES vs HC
Avolition	o| Gaiellales	Actinobacteria	0.327	0.048	FES vs HC
Avolition	f| Clostridiaceae 1	Firmicutes	0.352	0.044	FES vs HC
Avolition	g| Lactococcus	Firmicutes	0.376	0.037	FES vs HC
Avolition	g| unidentified Veillonellaceae	Firmicutes	0.431	0.004	FES vs HC
Avolition	f| Leptotrichiaceae	Fusobacteria	-0.297	0.024	FES vs HC
Avolition	g| Leptotrichia	Fusobacteria	-0.285	0.030	FES vs HC
Avolition	f| Gemmatimonadaceae	Gemmatimonadetes	0.335	0.040	FES vs HC
Avolition	o| Gemmatimonadales	Gemmatimonadetes	0.335	0.040	FES vs HC
Avolition	f| Rhodospirillaceae	Proteobacteria	0.470	0.006	FES vs HC
BPRS	c| Flavobacteriia	Bacteroidetes	-0.269	0.041	FES vs HC
BPRS	f| Flavobacteriaceae	Bacteroidetes	-0.270	0.040	FES vs HC
BPRS	g| Chryseobacterium	Bacteroidetes	-0.291	0.035	FES vs HC
BPRS	o| Flavobacteriales	Bacteroidetes	-0.269	0.041	FES vs HC
BPRS	f| Fusobacteriaceae	Fusobacteria	-0.261	0.048	FES vs HC
BPRS	g| Fusobacterium	Fusobacteria	-0.259	0.050	FES vs HC
BPRS	c| Epsilonproteobacteria	Proteobacteria	-0.269	0.041	FES vs HC
BPRS	f| Campylobacteraceae	Proteobacteria	-0.271	0.040	FES vs HC
BPRS	g| Actinobacillus	Proteobacteria	-0.280	0.033	FES vs HC
BPRS	g| Campylobacter	Proteobacteria	-0.269	0.041	FES vs HC
BPRS	g| Haemophilus	Proteobacteria	-0.338	0.009	FES vs HC
BPRS	g| Stenotrophomonas	Proteobacteria	-0.272	0.040	FES vs HC
BPRS	o| Campylobacterales	Proteobacteria	-0.269	0.041	FES vs HC
BPRS	g| Candidatus Saccharimonas	Saccharibacteria	-0.338	0.009	FES vs HC
SANS	f| Peptococcaceae	Firmicutes	-0.277	0.037	FES vs HC
SANS	f| Rhodospirillaceae	Proteobacteria	0.430	0.012	FES vs HC
SANS	o| Aeromonadales	Proteobacteria	0.355	0.039	FES vs HC
CGI	c| unidentified Acidobacteria	Acidobacteria	0.250	0.029	FES vs HC
CGI	p| Acidobacteria	Acidobacteria	0.233	0.043	FES vs HC
CGI	g| Micrococcus	Actinobacteria	0.281	0.042	FES vs HC
CGI	g| Alloprevotella	Bacteroidetes	-0.261	0.022	FES vs HC
CGI	c| Chloroplast	Cyanobacteria	0.252	0.048	FES vs HC
CGI	p| Cyanobacteria	Cyanobacteria	0.289	0.021	FES vs HC
CGI	f| Clostridiaceae 1	Firmicutes	0.330	0.033	FES vs HC
CGI	g| Clostridium sensu stricto 1	Firmicutes	0.362	0.025	FES vs HC
CGI	c| Deltaproteobacteria	Proteobacteria	0.245	0.035	FES vs HC
CGI	f| Hyphomicrobiaceae	Proteobacteria	0.279	0.038	FES vs HC
CGI	f| Moraxellaceae	Proteobacteria	-0.228	0.046	FES vs HC
CGI	f| Pasteurellaceae	Proteobacteria	-0.267	0.019	FES vs HC
CGI	g| Kingella	Proteobacteria	-0.275	0.018	FES vs HC
CGI	g| Paracocccus	Proteobacteria	0.288	0.043	FES vs HC
CGI	g| Sphingomonas	Proteobacteria	0.246	0.032	FES vs HC

CGI	o| Pasteurellales	Proteobacteria	-0.267	0.019	FES vs HC
DS	g| Corynebacterium	Actinobacteria	-0.314	0.049	CHR vs HC
DS	c| unidentified Candidate division SR1	Candidate division SR1	0.412	0.008	CHR vs HC
DS	f| unidentified Candidate division SR1	Candidate division SR1	0.412	0.008	CHR vs HC
DS	g| unidentified Candidate division SR1	Candidate division SR1	0.412	0.008	CHR vs HC
DS	o| unidentified Candidate division SR1	Candidate division SR1	0.412	0.008	CHR vs HC
DS	p| Candidate division SR1	Candidate division SR1	0.412	0.008	CHR vs HC
DS	g| Johnsonella	Firmicutes	0.320	0.044	CHR vs HC
DS	g| Solobacterium	Firmicutes	0.321	0.043	CHR vs HC
DS	g| unidentified Lachnospiraceae	Firmicutes	-0.538	0.001	CHR vs HC
DS	f| Bradyrhizobiaceae	Proteobacteria	0.394	0.035	CHR vs HC
DS	g| Mycoplasma	Tenericutes	-0.411	0.030	CHR vs HC
GS	c| Sphingobacteriia	Bacteroidetes	0.399	0.018	CHR vs HC
GS	f| Sphingobacteriaceae	Bacteroidetes	0.362	0.045	CHR vs HC
GS	g| Sphingobacterium	Bacteroidetes	0.468	0.016	CHR vs HC
GS	o| Sphingobacteriales	Bacteroidetes	0.399	0.018	CHR vs HC
GS	f| Family XIII-1	Firmicutes	0.360	0.037	CHR vs HC
GS	f| Planococcaceae	Firmicutes	0.486	0.014	CHR vs HC
GS	f| DA111	Proteobacteria	0.481	0.011	CHR vs HC
GS	g| Mycoplasma	Tenericutes	-0.410	0.030	CHR vs HC
NS	p| Cyanobacteria	Cyanobacteria	0.455	0.033	CHR vs HC
NS	c| Deinococci	Deinococcus-Thermus	-0.465	0.039	CHR vs HC
NS	p| Deinococcus-Thermus	Deinococcus-Thermus	-0.465	0.039	CHR vs HC
NS	g| Brevibacillus	Firmicutes	0.364	0.034	CHR vs HC
NS	g| Faecalibacterium	Firmicutes	-0.335	0.037	CHR vs HC
NS	f| Rhodocyclaceae	Proteobacteria	0.422	0.040	CHR vs HC
NS	o| Rhodocyclales	Proteobacteria	0.422	0.040	CHR vs HC
NS	c| unidentified Spirochaetes	Spirochaetes	0.327	0.045	CHR vs HC
NS	f| Spirochaetaceae	Spirochaetes	0.358	0.027	CHR vs HC
NS	g| Treponema 2	Spirochaetes	0.344	0.035	CHR vs HC
NS	o| Spirochaetales	Spirochaetes	0.327	0.045	CHR vs HC
NS	p| Spirochaetes	Spirochaetes	0.327	0.045	CHR vs HC
PS	g| Shuttleworthia	Firmicutes	0.461	0.005	CHR vs HC
PS	o| Bacillales	Firmicutes	0.323	0.042	CHR vs HC
PS	p| Firmicutes	Firmicutes	0.317	0.046	CHR vs HC
SIPS	g| Tannerella	Bacteroidetes	0.329	0.041	CHR vs HC
SIPS	g| Brevibacillus	Firmicutes	0.360	0.036	CHR vs HC
SIPS	f| Bradyrhizobiaceae	Proteobacteria	0.388	0.037	CHR vs HC
SIPS	c| unidentified Spirochaetes	Spirochaetes	0.411	0.010	CHR vs HC
SIPS	f| Spirochaetaceae	Spirochaetes	0.445	0.005	CHR vs HC
SIPS	g| Treponema 2	Spirochaetes	0.433	0.007	CHR vs HC
SIPS	o| Spirochaetales	Spirochaetes	0.411	0.010	CHR vs HC
SIPS	p| Spirochaetes	Spirochaetes	0.411	0.010	CHR vs HC

Supplementary Table 3. The distinct relationships between salivary taxa and blood markers in the FES and HC groups, respectively.

Blood markers	Taxa	Phylum	r	*p* -value	Group
CRP	f| Carnobacteriaceae	Firmicutes	-0.313	0.032	HC
CRP	g| Granulicatella	Firmicutes	-0.312	0.033	HC
CRP	c| unidentified Actinobacteria	Actinobacteria	-0.351	0.016	HC
CRP	o| Micrococcales	Actinobacteria	-0.384	0.008	HC
CRP	f| Micrococcaceae	Actinobacteria	-0.386	0.007	HC
CRP	g| Rothia	Actinobacteria	-0.381	0.008	HC
IFNγ	g| Silanimonas	Proteobacteria	0.297	0.043	HC
IL 1β	c| Clostridia	Firmicutes	0.339	0.020	HC
IL 1β	o| Clostridiales	Firmicutes	0.339	0.020	HC
IL 1β	f| Family XIII	Firmicutes	0.331	0.023	HC
IL 1β	f| Lachnospiraceae	Firmicutes	0.290	0.048	HC
IL 1β	g| Johnsonella	Firmicutes	0.310	0.034	HC
IL 1β	g| Lachnospiraceae NK4A136 group	Firmicutes	-0.341	0.034	HC
IL 1β	g| Mogibacterium	Firmicutes	0.355	0.014	HC
IL 1β	g| Peptoclostridium	Firmicutes	0.333	0.022	HC
IL 1β	g| Stomatobaculum	Firmicutes	0.297	0.043	HC
IL 1β	g| unidentified Family XIII	Firmicutes	0.307	0.036	HC
IL 1β	p| Bacteroidetes	Bacteroidetes	0.312	0.033	HC
IL 1β	c| Bacteroidia	Bacteroidetes	0.295	0.044	HC
IL 1β	o| Bacteroidales	Bacteroidetes	0.295	0.044	HC
IL 1β	f| Porphyromonadaceae	Bacteroidetes	0.298	0.042	HC
IL 1β	g| Porphyromonas	Bacteroidetes	0.298	0.042	HC
IL 1β	g| Brachybacterium	Actinobacteria	0.869	0.025	HC
IL 1β	g| Mobiluncus	Actinobacteria	0.325	0.029	HC
IL 8	f| Bacillaceae	Firmicutes	0.293	0.045	HC
IL 8	g| Anoxybacillus	Firmicutes	0.311	0.033	HC
IL 8	g| Geobacillus	Firmicutes	0.404	0.022	HC
IL 8	c| Deltaproteobacteria	Proteobacteria	0.356	0.014	HC
IL 8	o| Sphingomonadales	Proteobacteria	0.294	0.045	HC
IL 8	f| Comamonadaceae	Proteobacteria	-0.299	0.041	HC
IL 8	g| Silanimonas	Proteobacteria	0.394	0.006	HC
S100B	p| Chloroflexi	Chloroflexi	-0.533	0.007	HC
S100B	g| Brachybacterium	Actinobacteria	0.852	0.031	HC
Thioredioxin	g| Prevotellaceae UCG-004	Bacteroidetes	0.599	0.039	HC
Thioredioxin	g| Acidovorax	Proteobacteria	-0.307	0.038	HC
Thioredioxin	g| Pseudomonas	Proteobacteria	-0.373	0.010	HC
TNFα	f| Bacillaceae	Firmicutes	0.328	0.025	HC
TNFα	g| Anoxybacillus	Firmicutes	0.361	0.013	HC
TNFα	g| Geobacillus	Firmicutes	0.366	0.039	HC
TNFα	c| unidentified Spirochaetes	Spirochaetes	0.361	0.014	HC
TNFα	o| Spirochaetales	Spirochaetes	0.361	0.014	HC
TNFα	f| Spirochaetaceae	Spirochaetes	0.361	0.014	HC
TNFα	g| Brachybacterium	Actinobacteria	0.860	0.028	HC
TNFα	o| Sphingomonadales	Proteobacteria	0.318	0.030	HC
TNFα	f| Sphingomonadaceae	Proteobacteria	0.310	0.034	HC
TNFα	g| Silanimonas	Proteobacteria	0.420	0.003	HC
CRP	p| Firmicutes	Firmicutes	0.334	0.026	FES
CRP	f| Carnobacteriaceae	Firmicutes	0.308	0.042	FES
CRP	g| Granulicatella	Firmicutes	0.308	0.042	FES
CRP	p| Thermomicrobia	Thermomicrobia	-0.161	0.017	FES
CRP	p| Bacteroidetes	Bacteroidetes	-0.326	0.031	FES
CRP	o| Propionibacteriales	Actinobacteria	0.481	0.002	FES
CRP	f| Propionibacteriaceae	Actinobacteria	0.360	0.026	FES
CRP	g| Haemophilus	Proteobacteria	-0.384	0.010	FES
IFNγ	g| Megasphaera	Firmicutes	-0.382	0.011	FES
IFNγ	g| Veillonella	Firmicutes	-0.352	0.019	FES
IFNγ	c| Epsilonproteobacteria	Proteobacteria	-0.300	0.048	FES
IFNγ	o| Campylobacterales	Proteobacteria	-0.300	0.048	FES
IFNγ	f| Campylobacteraceae	Proteobacteria	-0.307	0.043	FES
IFNγ	g| Campylobacter	Proteobacteria	-0.307	0.042	FES
IFNγ	g| Haemophilus	Proteobacteria	-0.375	0.012	FES
IL 1β	f| Porphyromonadaceae	Bacteroidetes	0.320	0.034	FES

IL 1β	g| Porphyromonas	Bacteroidetes	0.315	0.037	FES
IL 1β	g| Brachybacterium	Actinobacteria	0.651	0.030	FES
IL 8	g| Megasphaera	Firmicutes	-0.459	0.002	FES
IL 8	g| Veillonella	Firmicutes	-0.325	0.031	FES
IL 8	p| Saccharibacteria	Saccharibacteria	-0.328	0.030	FES
IL 8	p| Bacteroidetes	Bacteroidetes	-0.326	0.031	FES
IL 8	g| Haemophilus	Proteobacteria	-0.324	0.032	FES
S100B	f| Spirochaetaceae	Spirochaetes	0.309	0.047	FES
S100B	g| Treponema 2	Spirochaetes	0.323	0.037	FES
S100B	o| Mycoplasmatales	Tenericutes	0.390	0.011	FES
S100B	f| Mycoplasmataceae	Tenericutes	0.390	0.011	FES
S100B	g| Mycoplasma	Tenericutes	0.385	0.012	FES
S100B	g| Prevotellaceae UCG-004	Bacteroidetes	0.477	0.039	FES
S100B	g| Lautropia	Proteobacteria	-0.311	0.040	FES
Thioredioxin	o| Bacillales	Firmicutes	-0.319	0.035	FES
Thioredioxin	g| Parvimonas	Firmicutes	-0.342	0.023	FES
Thioredioxin	c| unidentified Acidobacteria	Acidobacteria	-0.307	0.043	FES
Thioredioxin	p| Actinobacteria	Actinobacteria	-0.376	0.012	FES
Thioredioxin	c| unidentified Actinobacteria	Actinobacteria	-0.382	0.011	FES
Thioredioxin	o| Micrococcales	Actinobacteria	-0.393	0.008	FES
Thioredioxin	f| Micrococcaceae	Actinobacteria	-0.381	0.011	FES
Thioredioxin	g| Rothia	Actinobacteria	-0.381	0.011	FES
Thioredioxin	p| Proteobacteria	Proteobacteria	0.317	0.036	FES
TNFα	g| Catonella	Firmicutes	-0.308	0.042	FES
TNFα	g| Megasphaera	Firmicutes	-0.458	0.002	FES
TNFα	g| Selenomonas 4	Firmicutes	-0.394	0.008	FES
TNFα	g| Veillonella	Firmicutes	-0.379	0.011	FES
TNFα	f| Fusobacteriaceae	Fusobacteria	-0.331	0.028	FES
TNFα	g| Fusobacterium	Fusobacteria	-0.330	0.029	FES
TNFα	p| Saccharibacteria	Saccharibacteria	-0.453	0.002	FES
TNFα	p| Bacteroidetes	Bacteroidetes	-0.404	0.007	FES
TNFα	c| Bacteroidia	Bacteroidetes	-0.331	0.028	FES
TNFα	o| Bacteroidales	Bacteroidetes	-0.331	0.028	FES
TNFα	f| Prevotellaceae	Bacteroidetes	-0.305	0.044	FES
TNFα	o| Campylobacterales	Proteobacteria	-0.383	0.010	FES
TNFα	f| Campylobacteraceae	Proteobacteria	-0.382	0.011	FES
TNFα	g| Aggregatibacter	Proteobacteria	-0.319	0.035	FES
TNFα	g| Campylobacter	Proteobacteria	-0.382	0.011	FES
TNFα	g| Haemophilus	Proteobacteria	-0.434	0.003	FES

Supplementary Table 4. Differential KEGG pathways between FES and HC, CHR and HC, and FES and CHR, as detected in the DESeq2 package. All pathways with an FDR-adjusted *q* < 0.05 are included in the table.

KEGG pathway at level 3	KEGG pathway at level 2	KEGG pathway at level 1	baseMean	log2FoldChange	lfcSE	*p*-value	*q*-value	Comparison
Meiosis - yeast	Cell Growth and Death	Cellular Processes	4245.095	0.380204616	0.14314	0.007903	0.015282	FES vs HC
p53 signaling pathway	Cell Growth and Death	Cellular Processes	950.1711	-0.534363734	0.224109	0.017107	0.029558	FES vs HC
Bacterial motility proteins	Cell Motility	Cellular Processes	338845.3	-0.197360198	0.083265	0.017776	0.030532	FES vs HC
Peroxisome	Transport and Catabolism	Cellular Processes	70909.2	-0.128271677	0.04104	0.001775	0.004387	FES vs HC
Bacterial secretion system	Membrane Transport	Environmental Information Processing	229993.9	-0.077402017	0.028995	0.007596	0.014887	FES vs HC
Phosphotransferase system (PTS)	Membrane Transport	Environmental Information Processing	119366.7	0.296597801	0.107938	0.005999	0.012335	FES vs HC
Secretion system	Membrane Transport	Environmental Information Processing	520527.6	-0.131104298	0.036695	0.000353	0.001164	FES vs HC
Transporters	Membrane Transport	Environmental Information Processing	1566809	0.122010263	0.037125	0.001014	0.002768	FES vs HC
Two-component system	Signal Transduction	Environmental Information Processing	453914.2	-0.183719856	0.060837	0.002529	0.005814	FES vs HC
Bacterial toxins	Signaling Molecules and Interaction	Environmental Information Processing	26689.87	0.368023726	0.084751	1.41E-05	9.15E-05	FES vs HC
Cellular antigens	Signaling Molecules and Interaction	Environmental Information Processing	16086.02	-0.185322844	0.053935	0.00059	0.001777	FES vs HC
Ion channels	Signaling Molecules and Interaction	Environmental Information Processing	8958.685	0.209904383	0.093313	0.024483	0.040164	FES vs HC
Chaperones and folding catalysts	Folding	Genetic Information Processing	324011.1	-0.038355488	0.017383	0.027348	0.04412	FES vs HC
Proteasome	Folding	Genetic Information Processing	8316.549	0.210877358	0.068149	0.001972	0.004721	FES vs HC
Protein export	Folding	Genetic Information Processing	188349.9	0.12165316	0.030326	6.03E-05	0.000284	FES vs HC
RNA degradation	Folding	Genetic Information Processing	135082.7	0.051520545	0.017783	0.003765	0.008265	FES vs HC

Sulfur relay system	Folding	Genetic Information Processing	94637.73	-0.07152801	0.031203	0.021885	0.036183	FES vs HC
Base excision repair	Replication and Repair	Genetic Information Processing	139652.1	0.079931796	0.024471	0.001089	0.002918	FES vs HC
DNA repair and recombination proteins	Replication and Repair	Genetic Information Processing	829138.5	0.093264381	0.021734	1.78E-05	0.00011	FES vs HC
DNA replication	Replication and Repair	Genetic Information Processing	190060.6	0.119060079	0.02945	5.28E-05	0.000257	FES vs HC
DNA replication proteins	Replication and Repair	Genetic Information Processing	333105.5	0.10155941	0.029314	0.000531	0.001686	FES vs HC
Homologous recombination	Replication and Repair	Genetic Information Processing	269222.5	0.116244937	0.027672	2.66E-05	0.000152	FES vs HC
Mismatch repair	Replication and Repair	Genetic Information Processing	219966.8	0.114518181	0.027851	3.93E-05	0.00021	FES vs HC
Non-homologous end-joining	Replication and Repair	Genetic Information Processing	7120.519	-0.709819866	0.123078	8.06E-09	5.88E-07	FES vs HC
Nucleotide excision repair	Replication and Repair	Genetic Information Processing	104055.3	0.141872295	0.031249	5.62E-06	5.3E-05	FES vs HC
RNA polymerase	Transcription	Genetic Information Processing	50097.04	0.179560231	0.038499	3.1E-06	3.75E-05	FES vs HC
Transcription factors	Transcription	Genetic Information Processing	380812.1	-0.061748562	0.025557	0.015688	0.027267	FES vs HC
Transcription machinery	Transcription	Genetic Information Processing	203540.2	0.044511687	0.016306	0.006336	0.01276	FES vs HC
Aminoacyl-tRNA biosynthesis	Translation	Genetic Information Processing	345808.8	0.150975042	0.033802	7.95E-06	6.83E-05	FES vs HC
Ribosome	Translation	Genetic Information Processing	672565.7	0.157989182	0.036292	1.34E-05	9.15E-05	FES vs HC
Translation factors	Translation	Genetic Information Processing	151608.5	0.128517366	0.034729	0.000215	0.000766	FES vs HC
Colorectal cancer	Cancers	Human Diseases	911.4571	-0.587338332	0.221645	0.008051	0.015282	FES vs HC
Small cell lung cancer	Cancers	Human Diseases	913.3333	-0.58037368	0.221753	0.008865	0.016281	FES vs HC
Viral myocarditis	Cardiovascular Diseases	Human Diseases	912.7365	-0.586210722	0.221747	0.008203	0.015342	FES vs HC
African trypanosomiasis	Infectious Diseases	Human Diseases	3219.036	-0.636958658	0.155821	4.36E-05	0.000219	FES vs HC
Chagas disease (American trypanosomiasis)	Infectious Diseases	Human Diseases	2468.933	-0.376418564	0.148924	0.011485	0.020575	FES vs HC
Influenza A	Infectious Diseases	Human Diseases	914.1679	-0.584999308	0.221743	0.008335	0.015404	FES vs HC
Pertussis	Infectious Diseases	Human Diseases	11182.47	-0.700140275	0.16096	1.36E-05	9.15E-05	FES vs HC
Shigellosis	Infectious Diseases	Human Diseases	64.18368	-1.19650815	0.331773	0.00031	0.001042	FES vs HC

Staphylococcus aureus infection	Infectious Diseases	Human Diseases	12407.88	0.618358288	0.1626	0.000143	0.000567	FES vs HC
Toxoplasmosis	Infectious Diseases	Human Diseases	911.5348	-0.587077106	0.221655	0.008082	0.015282	FES vs HC
Tuberculosis	Infectious Diseases	Human Diseases	37803.36	0.091418069	0.030405	0.002641	0.005979	FES vs HC
Vibrio cholerae infection	Infectious Diseases	Human Diseases	462.4375	-0.769946041	0.219288	0.000446	0.001448	FES vs HC
Vibrio cholerae pathogenic cycle	Infectious Diseases	Human Diseases	24209.15	-0.14918128	0.054598	0.006288	0.012751	FES vs HC
Type I diabetes mellitus	Metabolic Diseases	Human Diseases	13663.68	0.180103695	0.03876	3.37E-06	3.75E-05	FES vs HC
Type II diabetes mellitus	Metabolic Diseases	Human Diseases	12838.71	0.191003342	0.072148	0.008112	0.015282	FES vs HC
Amyotrophic lateral sclerosis (ALS)	Neurodegenerative Diseases	Human Diseases	11836.29	-0.288597308	0.085891	0.000779	0.002276	FES vs HC
Huntington's disease	Neurodegenerative Diseases	Human Diseases	32149.97	-0.155471209	0.0513	0.002441	0.005656	FES vs HC
Parkinson's disease	Neurodegenerative Diseases	Human Diseases	15753.59	-0.25794094	0.105847	0.014813	0.026056	FES vs HC
Prion diseases	Neurodegenerative Diseases	Human Diseases	2894.77	-0.490315548	0.128734	0.00014	0.000567	FES vs HC
Alanine, aspartate and glutamate metabolism	Amino Acid Metabolism	Metabolism	264457.3	0.028744853	0.012544	0.021933	0.036183	FES vs HC
Amino acid related enzymes	Amino Acid Metabolism	Metabolism	423903.4	0.077189027	0.019821	9.84E-05	0.000436	FES vs HC
Arginine and proline metabolism	Amino Acid Metabolism	Metabolism	310776.7	-0.075280939	0.024052	0.001748	0.004387	FES vs HC
Cysteine and methionine metabolism	Amino Acid Metabolism	Metabolism	260043.6	0.05025873	0.018979	0.008095	0.015282	FES vs HC
Histidine metabolism	Amino Acid Metabolism	Metabolism	153704.4	-0.066690663	0.027312	0.014612	0.025859	FES vs HC
Lysine biosynthesis	Amino Acid Metabolism	Metabolism	192619.7	0.065758484	0.020799	0.001569	0.004053	FES vs HC
Lysine degradation	Amino Acid Metabolism	Metabolism	96883.31	-0.434022218	0.096559	6.96E-06	6.16E-05	FES vs HC
Phenylalanine metabolism	Amino Acid Metabolism	Metabolism	67728.5	-0.262434309	0.060082	1.25E-05	9.15E-05	FES vs HC
Tryptophan metabolism	Amino Acid Metabolism	Metabolism	117770.1	-0.374181603	0.086047	1.37E-05	9.15E-05	FES vs HC
Tyrosine metabolism	Amino Acid Metabolism	Metabolism	121881.9	-0.146342253	0.040124	0.000265	0.0009	FES vs HC

Valine, leucine and isoleucine biosynthesis	Amino Acid Metabolism	Metabolism	219792.6	0.057866092	0.022545	0.010266	0.018619	FES vs HC
Valine, leucine and isoleucine degradation	Amino Acid Metabolism	Metabolism	167075	-0.43529016	0.089548	1.17E-06	2.01E-05	FES vs HC
beta-Lactam resistance	Biosynthesis of Other Secondary Metabolites	Metabolism	6161.451	-0.283837707	0.099664	0.0044	0.009447	FES vs HC
Betalain biosynthesis	Biosynthesis of Other Secondary Metabolites	Metabolism	135.1716	-1.024684877	0.302543	0.000707	0.002106	FES vs HC
Butirosin and neomycin biosynthesis	Biosynthesis of Other Secondary Metabolites	Metabolism	12052.52	0.275181215	0.083216	0.000944	0.002624	FES vs HC
Caffeine metabolism	Biosynthesis of Other Secondary Metabolites	Metabolism	615.6156	0.806526437	0.209372	0.000117	0.000488	FES vs HC
Flavone and flavonol biosynthesis	Biosynthesis of Other Secondary Metabolites	Metabolism	741.4719	0.491467561	0.175014	0.004983	0.010467	FES vs HC
Flavonoid biosynthesis	Biosynthesis of Other Secondary Metabolites	Metabolism	1600.815	0.43034265	0.150263	0.004184	0.00905	FES vs HC
Isoflavonoid biosynthesis	Biosynthesis of Other Secondary Metabolites	Metabolism	98.84446	-1.285273203	0.314113	4.28E-05	0.000219	FES vs HC
Isoquinoline alkaloid biosynthesis	Biosynthesis of Other Secondary Metabolites	Metabolism	16799.79	-0.112299815	0.042146	0.00771	0.015009	FES vs HC
Stilbenoid, diarylheptanoid and gingerol biosynthesis	Biosynthesis of Other Secondary Metabolites	Metabolism	1566.539	0.476337084	0.169194	0.004873	0.01031	FES vs HC
Streptomycin biosynthesis	Biosynthesis of Other Secondary Metabolites	Metabolism	81896.03	0.145949227	0.046931	0.001872	0.004555	FES vs HC
Tropane, piperidine and pyridine alkaloid biosynthesis	Biosynthesis of Other Secondary Metabolites	Metabolism	34528.98	-0.167012521	0.043884	0.000141	0.000567	FES vs HC

Amino sugar and nucleotide sugar metabolism	Carbohydrate Metabolism	Metabolism	319258.3	0.213646683	0.046036	3.47E-06	3.75E-05	FES vs HC
Ascorbate and aldarate metabolism	Carbohydrate Metabolism	Metabolism	34376.51	-0.19565637	0.062642	0.001788	0.004387	FES vs HC
Butanoate metabolism	Carbohydrate Metabolism	Metabolism	246116.3	-0.141176789	0.040971	0.000569	0.00175	FES vs HC
C5-Branched dibasic acid metabolism	Carbohydrate Metabolism	Metabolism	91665.01	0.055805453	0.023677	0.018426	0.031464	FES vs HC
Fructose and mannose metabolism	Carbohydrate Metabolism	Metabolism	178658.9	0.220661145	0.050133	1.08E-05	8.13E-05	FES vs HC
Galactose metabolism	Carbohydrate Metabolism	Metabolism	127874.3	0.313719043	0.077588	5.27E-05	0.000257	FES vs HC
Glycolysis / Gluconeogenesis	Carbohydrate Metabolism	Metabolism	315172.8	0.101802219	0.026777	0.000144	0.000567	FES vs HC
Glyoxylate and dicarboxylate metabolism	Carbohydrate Metabolism	Metabolism	169909.4	-0.20924995	0.041409	4.34E-07	9.49E-06	FES vs HC
Inositol phosphate metabolism	Carbohydrate Metabolism	Metabolism	45895.97	-0.117304023	0.038884	0.002555	0.005829	FES vs HC
Pentose phosphate pathway	Carbohydrate Metabolism	Metabolism	204399.3	0.120743196	0.032184	0.000176	0.000658	FES vs HC
Propanoate metabolism	Carbohydrate Metabolism	Metabolism	226362	-0.187916575	0.044825	2.76E-05	0.000155	FES vs HC
Starch and sucrose metabolism	Carbohydrate Metabolism	Metabolism	172871.2	0.249523509	0.052458	1.97E-06	3.03E-05	FES vs HC
Carbon fixation in photosynthetic organisms	Energy Metabolism	Metabolism	152559.9	0.113339191	0.030442	0.000197	0.000727	FES vs HC
Methane metabolism	Energy Metabolism	Metabolism	292152	0.068590174	0.020431	0.000787	0.002276	FES vs HC
Nitrogen metabolism	Energy Metabolism	Metabolism	205748.6	-0.06823546	0.021509	0.001512	0.003977	FES vs HC
Photosynthesis	Energy Metabolism	Metabolism	102515.8	0.204166289	0.038065	8.15E-08	3.4E-06	FES vs HC
Photosynthesis proteins	Energy Metabolism	Metabolism	107024.6	0.174494454	0.033942	2.73E-07	8.87E-06	FES vs HC
Sulfur metabolism	Energy Metabolism	Metabolism	92128.66	-0.070609803	0.024625	0.004138	0.009017	FES vs HC
Cytochrome P450	Enzyme Families	Metabolism	27.65174	2.289385856	0.273396	5.57E-17	1.63E-14	FES vs HC

Peptidases	Enzyme Families	Metabolism	466530.6	0.100451372	0.023591	2.06E-05	0.000123	FES vs HC
Protein kinases	Enzyme Families	Metabolism	97016.09	-0.129755314	0.04631	0.005081	0.010597	FES vs HC
Glycosaminoglycan biosynthesis - chondroitin sulfate	Glycan Biosynthesis and Metabolism	Metabolism	34.40882	-0.652678621	0.208938	0.001785	0.004387	FES vs HC
Glycosaminoglycan degradation	Glycan Biosynthesis and Metabolism	Metabolism	6814.51	0.420306113	0.161713	0.009347	0.017058	FES vs HC
Glycosphingolipid biosynthesis - ganglio series	Glycan Biosynthesis and Metabolism	Metabolism	5210.228	0.379413789	0.164009	0.020702	0.034742	FES vs HC
Glycosphingolipid biosynthesis - globo series	Glycan Biosynthesis and Metabolism	Metabolism	11378.54	0.471643227	0.153352	0.002101	0.004988	FES vs HC
Glycosyltransferases	Glycan Biosynthesis and Metabolism	Metabolism	115625.3	0.086465467	0.027409	0.001607	0.004117	FES vs HC
Lipopolysaccharide biosynthesis	Glycan Biosynthesis and Metabolism	Metabolism	125595.7	-0.160112347	0.050591	0.001552	0.004045	FES vs HC
Lipopolysaccharide biosynthesis proteins	Glycan Biosynthesis and Metabolism	Metabolism	165222.5	-0.123483298	0.040511	0.002303	0.005379	FES vs HC
N-Glycan biosynthesis	Glycan Biosynthesis and Metabolism	Metabolism	6303.29	0.480230671	0.143741	0.000835	0.002367	FES vs HC
Other glycan degradation	Glycan Biosynthesis and Metabolism	Metabolism	22149.61	0.435410099	0.145975	0.002857	0.006367	FES vs HC
Peptidoglycan biosynthesis	Glycan Biosynthesis and Metabolism	Metabolism	242507.4	0.119857706	0.032371	0.000213	0.000766	FES vs HC
alpha-Linolenic acid metabolism	Lipid Metabolism	Metabolism	8564.818	-0.503170063	0.100734	5.88E-07	1.15E-05	FES vs HC
Arachidonic acid metabolism	Lipid Metabolism	Metabolism	18392.76	-0.22756765	0.057439	7.43E-05	0.000345	FES vs HC
Biosynthesis of unsaturated fatty acids	Lipid Metabolism	Metabolism	66748.87	-0.305514749	0.079285	0.000117	0.000488	FES vs HC

Fatty acid metabolism	Lipid Metabolism	Metabolism	149183.7	-0.408063095	0.086347	2.29E-06	3.19E-05	FES vs HC
Glycerolipid metabolism	Lipid Metabolism	Metabolism	99380.7	0.09329859	0.020342	4.51E-06	4.54E-05	FES vs HC
Linoleic acid metabolism	Lipid Metabolism	Metabolism	13971.34	-0.290724403	0.119759	0.0152	0.026577	FES vs HC
Lipid biosynthesis proteins	Lipid Metabolism	Metabolism	214732.2	-0.037480441	0.016819	0.025854	0.042176	FES vs HC
Primary bile acid biosynthesis	Lipid Metabolism	Metabolism	3009.205	-1.025465724	0.202628	4.17E-07	9.49E-06	FES vs HC
Secondary bile acid biosynthesis	Lipid Metabolism	Metabolism	900.8071	-1.004063512	0.228232	1.09E-05	8.13E-05	FES vs HC
Sphingolipid metabolism	Lipid Metabolism	Metabolism	17259.74	0.405624653	0.113566	0.000355	0.001164	FES vs HC
Steroid biosynthesis	Lipid Metabolism	Metabolism	620.1883	-0.523609779	0.225174	0.020053	0.033847	FES vs HC
Steroid hormone biosynthesis	Lipid Metabolism	Metabolism	2182.168	-0.49139461	0.177625	0.005667	0.011735	FES vs HC
Synthesis and degradation of ketone bodies	Lipid Metabolism	Metabolism	31949.74	-0.507812559	0.111371	5.12E-06	4.99E-05	FES vs HC
Lipoic acid metabolism	Metabolism of Cofactors and Vitamins	Metabolism	21487.73	0.077125976	0.033013	0.019481	0.033072	FES vs HC
One carbon pool by folate	Metabolism of Cofactors and Vitamins	Metabolism	168040	0.092689642	0.02468	0.000173	0.000658	FES vs HC
Pantothenate and CoA biosynthesis	Metabolism of Cofactors and Vitamins	Metabolism	173270.6	0.075941346	0.023935	0.001509	0.003977	FES vs HC
Retinol metabolism	Metabolism of Cofactors and Vitamins	Metabolism	26817.6	-0.242840718	0.060461	5.91E-05	0.000283	FES vs HC
Riboflavin metabolism	Metabolism of Cofactors and Vitamins	Metabolism	78460.11	-0.071036608	0.019312	0.000235	0.000816	FES vs HC
Thiamine metabolism	Metabolism of Cofactors and Vitamins	Metabolism	119074.1	0.092204972	0.027454	0.000784	0.002276	FES vs HC
Ubiquinone and other terpenoid-quinone biosynthesis	Metabolism of Cofactors and Vitamins	Metabolism	89695.59	-0.154685662	0.030622	4.39E-07	9.49E-06	FES vs HC

beta-Alanine metabolism	Metabolism of Other Amino Acids	Metabolism	91167.79	-0.392605986	0.084372	3.27E-06	3.75E-05	FES vs HC
D-Alanine metabolism	Metabolism of Other Amino Acids	Metabolism	32412.23	0.182972324	0.044391	3.76E-05	0.000207	FES vs HC
D-Glutamine and D-glutamate metabolism	Metabolism of Other Amino Acids	Metabolism	43655.84	0.068350208	0.024134	0.004624	0.009856	FES vs HC
Glutathione metabolism	Metabolism of Other Amino Acids	Metabolism	120332	-0.224796971	0.041149	4.68E-08	2.28E-06	FES vs HC
Selenocompound metabolism	Metabolism of Other Amino Acids	Metabolism	102507.8	0.112098654	0.026052	1.69E-05	0.000107	FES vs HC
Taurine and hypotaurine metabolism	Metabolism of Other Amino Acids	Metabolism	36970.21	0.07963222	0.025627	0.001887	0.004555	FES vs HC
Biosynthesis of ansamycins	Metabolism of Terpenoids and Polyketides	Metabolism	17157.81	0.168236935	0.040935	3.96E-05	0.00021	FES vs HC
Biosynthesis of vancomycin group antibiotics	Metabolism of Terpenoids and Polyketides	Metabolism	13780.99	0.238567611	0.063574	0.000175	0.000658	FES vs HC
Geraniol degradation	Metabolism of Terpenoids and Polyketides	Metabolism	68869.93	-0.712095402	0.140428	3.96E-07	9.49E-06	FES vs HC
Limonene and pinene degradation	Metabolism of Terpenoids and Polyketides	Metabolism	67942.31	-0.572319374	0.133627	1.84E-05	0.000112	FES vs HC
Polyketide sugar unit biosynthesis	Metabolism of Terpenoids and Polyketides	Metabolism	47869.91	0.169518819	0.055386	0.002208	0.0052	FES vs HC
Prenyltransferases	Metabolism of Terpenoids and Polyketides	Metabolism	92747.59	0.140045932	0.031685	9.87E-06	7.79E-05	FES vs HC
Terpenoid backbone biosynthesis	Metabolism of Terpenoids and Polyketides	Metabolism	160233.7	0.108330709	0.021992	8.4E-07	1.53E-05	FES vs HC

Zeatin biosynthesis	Metabolism of Terpenoids and Polyketides	Metabolism	13395.67	0.181893156	0.043067	2.41E-05	0.00014	FES vs HC
Purine metabolism	Nucleotide Metabolism	Metabolism	669683	0.097191039	0.022365	1.39E-05	9.15E-05	FES vs HC
Pyrimidine metabolism	Nucleotide Metabolism	Metabolism	496153.2	0.143929084	0.03255	9.79E-06	7.79E-05	FES vs HC
Aminobenzoate degradation	Xenobiotics Biodegradation and Metabolism	Metabolism	80896.95	-0.313835395	0.085392	0.000238	0.000816	FES vs HC
Atrazine degradation	Xenobiotics Biodegradation and Metabolism	Metabolism	12547.12	-0.407620849	0.135726	0.002671	0.006	FES vs HC
Benzoate degradation	Xenobiotics Biodegradation and Metabolism	Metabolism	112557.8	-0.356582537	0.096952	0.000235	0.000816	FES vs HC
Bisphenol degradation	Xenobiotics Biodegradation and Metabolism	Metabolism	22422.37	-0.324254857	0.122745	0.008249	0.015342	FES vs HC
Caprolactam degradation	Xenobiotics Biodegradation and Metabolism	Metabolism	44218.73	-0.639283237	0.139379	4.5E-06	4.54E-05	FES vs HC
Chloroalkane and chloroalkene degradation	Xenobiotics Biodegradation and Metabolism	Metabolism	60151.2	-0.260606882	0.058564	8.59E-06	7.17E-05	FES vs HC
Chlorocyclohexane and chlorobenzene degradation	Xenobiotics Biodegradation and Metabolism	Metabolism	15853.44	-0.357839154	0.13188	0.00666	0.013321	FES vs HC
Drug metabolism - cytochrome P450	Xenobiotics Biodegradation and Metabolism	Metabolism	48775.98	-0.425397125	0.084331	4.55E-07	9.49E-06	FES vs HC
Drug metabolism - other enzymes	Xenobiotics Biodegradation and Metabolism	Metabolism	74136.79	0.206742054	0.042826	1.38E-06	2.24E-05	FES vs HC
Ethylbenzene degradation	Xenobiotics Biodegradation and Metabolism	Metabolism	14173.21	-0.610894552	0.131223	3.23E-06	3.75E-05	FES vs HC
Fluorobenzoate degradation	Xenobiotics Biodegradation and Metabolism	Metabolism	9283.168	-0.620760515	0.18576	0.000833	0.002367	FES vs HC

Metabolism of xenobiotics by cytochrome P450	Xenobiotics Biodegradation and Metabolism	Metabolism	46784.02	-0.430787252	0.083094	2.17E-07	7.91E-06	FES vs HC
Naphthalene degradation	Xenobiotics Biodegradation and Metabolism	Metabolism	68498.67	-0.343583176	0.075847	5.9E-06	5.38E-05	FES vs HC
Styrene degradation	Xenobiotics Biodegradation and Metabolism	Metabolism	19536.43	-0.345224151	0.091811	0.00017	0.000658	FES vs HC
Cardiac muscle contraction	Circulatory System	Organismal Systems	14796.08	-0.237275984	0.106667	0.026118	0.042369	FES vs HC
Bile secretion	Digestive System	Organismal Systems	168.9367	-0.714280306	0.226635	0.001623	0.004122	FES vs HC
Mineral absorption	Digestive System	Organismal Systems	6858.067	-0.254013974	0.073655	0.000563	0.00175	FES vs HC
Insulin signaling pathway	Endocrine System	Organismal Systems	20968.03	0.132472214	0.032276	4.05E-05	0.000211	FES vs HC
PPAR signaling pathway	Endocrine System	Organismal Systems	41643.42	-0.145821934	0.059235	0.013826	0.024618	FES vs HC
Renin-angiotensin system	Endocrine System	Organismal Systems	742.9566	-0.843660802	0.214192	8.19E-05	0.000368	FES vs HC
Circadian rhythm - plant	Environmental Adaptation	Organismal Systems	751.2476	0.764470687	0.193324	7.67E-05	0.00035	FES vs HC
Cell motility and secretion	Cellular Processes and Signaling	Unclassified	69883.89	-0.126304254	0.046111	0.00616	0.012579	FES vs HC
Electron transfer carriers	Cellular Processes and Signaling	Unclassified	6777.965	0.433289005	0.131601	0.000993	0.002736	FES vs HC
Germination	Cellular Processes and Signaling	Unclassified	1072.463	1.184731816	0.199025	2.64E-09	2.57E-07	FES vs HC
Inorganic ion transport and metabolism	Cellular Processes and Signaling	Unclassified	97893.62	-0.20478792	0.043649	2.71E-06	3.6E-05	FES vs HC
Membrane and intracellular structural molecules	Cellular Processes and Signaling	Unclassified	216238.9	-0.215084998	0.037674	1.14E-08	6.63E-07	FES vs HC
Other ion-coupled transporters	Cellular Processes and Signaling	Unclassified	381053.9	-0.088689553	0.026701	0.000895	0.002513	FES vs HC

Other transporters	Cellular Processes and Signaling	Unclassified	83332.21	-0.069240505	0.025611	0.006861	0.013629	FES vs HC
Pores ion channels	Cellular Processes and Signaling	Unclassified	186507.8	-0.184958139	0.04982	0.000205	0.000749	FES vs HC
Signal transduction mechanisms	Cellular Processes and Signaling	Unclassified	125050.9	-0.050964648	0.020059	0.011063	0.01994	FES vs HC
Sporulation	Cellular Processes and Signaling	Unclassified	16937.76	0.903626242	0.143307	2.87E-10	4.19E-08	FES vs HC
Transcription related proteins	Genetic Information Processing	Unclassified	1934.966	-0.572608509	0.166433	0.000581	0.001766	FES vs HC
Translation proteins	Genetic Information Processing	Unclassified	287482	0.050893465	0.022053	0.021009	0.035055	FES vs HC
Biosynthesis and biodegradation of secondary metabolites	Metabolism	Unclassified	19026.4	-0.230492478	0.065835	0.000463	0.001487	FES vs HC
Carbohydrate metabolism	Metabolism	Unclassified	26553.63	0.246420463	0.071388	0.000557	0.001748	FES vs HC
Energy metabolism	Metabolism	Unclassified	238612.7	-0.092802322	0.024016	0.000111	0.000479	FES vs HC
Glycan biosynthesis and metabolism	Metabolism	Unclassified	21335.44	-0.231990439	0.070991	0.001084	0.002918	FES vs HC
Metabolism of cofactors and vitamins	Metabolism	Unclassified	52373.29	-0.115234502	0.029762	0.000108	0.000471	FES vs HC
Nucleotide metabolism	Metabolism	Unclassified	16030.04	0.288855448	0.097075	0.002924	0.006469	FES vs HC
Function unknown	Poorly Characterized	Unclassified	531215.1	-0.136451357	0.028816	2.19E-06	3.19E-05	FES vs HC
General function prediction only	Poorly Characterized	Unclassified	967939.5	-0.042842884	0.01589	0.007014	0.013839	FES vs HC
p53 signaling pathway	Cell Growth and Death	Cellular Processes	999.6633	-1.090656786	0.312076	0.000474	0.003748	CHR vs HC
Bacterial motility proteins	Cell Motility	Cellular Processes	351613	-0.302478406	0.10745	0.004877	0.016425	CHR vs HC
Endocytosis	Transport and Catabolism	Cellular Processes	628.2358	-1.022755914	0.370411	0.00576	0.018381	CHR vs HC

Two-component system	Signal Transduction	Environmental Information Processing	470295.8	-0.262949303	0.084721	0.001911	0.007887	CHR vs HC
Cellular antigens	Signaling Molecules and Interaction	Environmental Information Processing	16626.59	-0.273190815	0.076991	0.000388	0.003442	CHR vs HC
Ion channels	Signaling Molecules and Interaction	Environmental Information Processing	8646.602	0.41431089	0.1395	0.002978	0.01128	CHR vs HC
Protein export	Folding	Genetic Information Processing	185061.4	0.133334024	0.043021	0.00194	0.007894	CHR vs HC
RNA degradation	Folding	Genetic Information Processing	134511.8	0.071992455	0.025266	0.00438	0.014922	CHR vs HC
Sulfur relay system	Folding	Genetic Information Processing	96362.97	-0.099495414	0.041844	0.017417	0.043993	CHR vs HC
Chromosome	Replication and Repair	Genetic Information Processing	453179.6	0.060336851	0.024771	0.014861	0.039228	CHR vs HC
DNA repair and recombination proteins	Replication and Repair	Genetic Information Processing	816777	0.103492069	0.030458	0.000679	0.003837	CHR vs HC
DNA replication	Replication and Repair	Genetic Information Processing	186409.3	0.134577711	0.041633	0.001227	0.005758	CHR vs HC
DNA replication proteins	Replication and Repair	Genetic Information Processing	327166.9	0.113691825	0.041042	0.005604	0.018381	CHR vs HC
Homologous recombination	Replication and Repair	Genetic Information Processing	264878.7	0.143031757	0.038741	0.000222	0.002626	CHR vs HC
Mismatch repair	Replication and Repair	Genetic Information Processing	216191.3	0.136199429	0.039587	0.000581	0.003748	CHR vs HC
Non-homologous end-joining	Replication and Repair	Genetic Information Processing	7760.915	-0.758291632	0.174642	1.41E-05	0.000591	CHR vs HC
Nucleotide excision repair	Replication and Repair	Genetic Information Processing	101801.7	0.156303161	0.045194	0.000543	0.003748	CHR vs HC
RNA polymerase	Transcription	Genetic Information Processing	48507.87	0.18384889	0.053762	0.000627	0.003749	CHR vs HC
Aminoacyl-tRNA biosynthesis	Translation	Genetic Information Processing	337729.3	0.158657336	0.048587	0.001093	0.005521	CHR vs HC
mRNA surveillance pathway	Translation	Genetic Information Processing	8.069395	-3.101870577	0.728695	2.07E-05	0.000675	CHR vs HC
Ribosome	Translation	Genetic Information Processing	656702.4	0.179052447	0.051784	0.000545	0.003748	CHR vs HC
Translation factors	Translation	Genetic Information Processing	148416.8	0.148268588	0.049816	0.002917	0.011247	CHR vs HC
Colorectal cancer	Cancers	Human Diseases	975.8788	-1.083516816	0.315193	0.000587	0.003748	CHR vs HC
Small cell lung cancer	Cancers	Human Diseases	976.4718	-1.080264304	0.315305	0.000612	0.003748	CHR vs HC

Hypertrophic cardiomyopathy (HCM)	Cardiovascular Diseases	Human Diseases	333.9179	-2.127344345	0.330086	1.16E-10	3.39E-08	CHR vs HC
Viral myocarditis	Cardiovascular Diseases	Human Diseases	976.5525	-1.084783623	0.315184	0.000578	0.003748	CHR vs HC
Primary immunodeficiency	Immune System Diseases	Human Diseases	17442.5	0.117448821	0.048696	0.015871	0.041152	CHR vs HC
African trypanosomiasis	Infectious Diseases	Human Diseases	3540.098	-0.621961232	0.215165	0.003845	0.013572	CHR vs HC
Bacterial invasion of epithelial cells	Infectious Diseases	Human Diseases	520.5894	-0.76017863	0.243713	0.001814	0.007702	CHR vs HC
Chagas disease (American trypanosomiasis)	Infectious Diseases	Human Diseases	2507.895	-0.741561521	0.205616	0.00031	0.003096	CHR vs HC
Epithelial cell signaling in Helicobacter pylori infection	Infectious Diseases	Human Diseases	18935.53	0.242788892	0.057818	2.68E-05	0.000785	CHR vs HC
Influenza A	Infectious Diseases	Human Diseases	977.4291	-1.086268152	0.315106	0.000566	0.003748	CHR vs HC
Shigellosis	Infectious Diseases	Human Diseases	71.62971	-2.629425954	0.476856	3.51E-08	5.14E-06	CHR vs HC
Toxoplasmosis	Infectious Diseases	Human Diseases	976.201	-1.080075945	0.315315	0.000614	0.003748	CHR vs HC
Vibrio cholerae infection	Infectious Diseases	Human Diseases	467.8043	-1.163130707	0.31519	0.000224	0.002626	CHR vs HC
Type I diabetes mellitus	Metabolic Diseases	Human Diseases	13192.47	0.179090205	0.054929	0.001112	0.005525	CHR vs HC
Type II diabetes mellitus	Metabolic Diseases	Human Diseases	12609.09	0.275727705	0.115689	0.017156	0.043993	CHR vs HC
Alzheimer's disease	Neurodegenerative Diseases	Human Diseases	33670.87	-0.188275822	0.069284	0.006579	0.020505	CHR vs HC
Huntington's disease	Neurodegenerative Diseases	Human Diseases	32402.29	-0.282196065	0.070305	5.97E-05	0.001346	CHR vs HC
Parkinson's disease	Neurodegenerative Diseases	Human Diseases	15897.53	-0.449105918	0.147849	0.002385	0.009442	CHR vs HC
Prion diseases	Neurodegenerative Diseases	Human Diseases	3276.948	-0.451787686	0.177881	0.011091	0.030948	CHR vs HC
Alanine, aspartate and glutamate metabolism	Amino Acid Metabolism	Metabolism	266092.5	0.073321672	0.017646	3.25E-05	0.000866	CHR vs HC
Amino acid related enzymes	Amino Acid Metabolism	Metabolism	422263.5	0.121528173	0.028225	1.66E-05	0.00061	CHR vs HC

Cysteine and methionine metabolism	Amino Acid Metabolism	Metabolism		260052.6	0.106190477	0.027172	9.3E-05	0.00148	CHR vs HC
Lysine biosynthesis	Amino Acid Metabolism	Metabolism		191671.5	0.100457184	0.030857	0.001132	0.005526	CHR vs HC
Lysine degradation	Amino Acid Metabolism	Metabolism		106684.9	-0.444193502	0.141037	0.001636	0.007059	CHR vs HC
Phenylalanine, tyrosine and tryptophan biosynthesis	Amino Acid Metabolism	Metabolism		227816.7	0.132546524	0.02707	9.76E-07	7.15E-05	CHR vs HC
Tryptophan metabolism	Amino Acid Metabolism	Metabolism		127252.3	-0.40777963	0.126426	0.001258	0.005758	CHR vs HC
Valine, leucine and isoleucine degradation	Amino Acid Metabolism	Metabolism		182441.7	-0.47786205	0.127474	0.000178	0.002367	CHR vs HC
Indole alkaloid biosynthesis	Biosynthesis of Other Secondary Metabolites	Metabolism		25.56229	-1.308849144	0.404644	0.001218	0.005758	CHR vs HC
Novobiocin biosynthesis	Biosynthesis of Other Secondary Metabolites	Metabolism		38978.35	0.120562983	0.035169	0.000608	0.003748	CHR vs HC
Phenylpropanoid biosynthesis	Biosynthesis of Other Secondary Metabolites	Metabolism		13040.48	-0.469930639	0.151403	0.00191	0.007887	CHR vs HC
Amino sugar and nucleotide sugar metabolism	Carbohydrate Metabolism	Metabolism		309087.8	0.224306753	0.065953	0.000671	0.003837	CHR vs HC
Butanoate metabolism	Carbohydrate Metabolism	Metabolism		255600.4	-0.158634035	0.065023	0.014701	0.039158	CHR vs HC
C5-Branched dibasic acid metabolism	Carbohydrate Metabolism	Metabolism		91841.82	0.114598112	0.035627	0.001297	0.005847	CHR vs HC
Fructose and mannose metabolism	Carbohydrate Metabolism	Metabolism		173988.6	0.225842227	0.068651	0.001003	0.005247	CHR vs HC
Galactose metabolism	Carbohydrate Metabolism	Metabolism		120393	0.294487697	0.108786	0.006789	0.020938	CHR vs HC
Glyoxylate and dicarboxylate metabolism	Carbohydrate Metabolism	Metabolism		178314.8	-0.194534118	0.058501	0.000883	0.004792	CHR vs HC
Pentose phosphate pathway	Carbohydrate Metabolism	Metabolism		199735.4	0.125629051	0.045462	0.005721	0.018381	CHR vs HC

Propanoate metabolism	Carbohydrate Metabolism	Metabolism	235665	-0.221460024	0.068641	0.001254	0.005758	CHR vs HC
Starch and sucrose metabolism	Carbohydrate Metabolism	Metabolism	164049.8	0.227661181	0.07672	0.003003	0.01128	CHR vs HC
Carbon fixation in photosynthetic organisms	Energy Metabolism	Metabolism	150582.7	0.151175004	0.043694	0.000541	0.003748	CHR vs HC
Photosynthesis	Energy Metabolism	Metabolism	98503.9	0.182505095	0.050792	0.000327	0.003096	CHR vs HC
Photosynthesis proteins	Energy Metabolism	Metabolism	103507.6	0.149566844	0.044743	0.000829	0.004585	CHR vs HC
Protein kinases	Enzyme Families	Metabolism	99492.16	-0.182021927	0.068495	0.007874	0.023541	CHR vs HC
Glycosaminoglycan degradation	Glycan Biosynthesis and Metabolism	Metabolism	6446.345	0.528330329	0.227048	0.019968	0.049164	CHR vs HC
Glycosphingolipid biosynthesis - globo series	Glycan Biosynthesis and Metabolism	Metabolism	10470	0.533560393	0.219604	0.015114	0.039538	CHR vs HC
Glycosphingolipid biosynthesis - lacto and neolacto series	Glycan Biosynthesis and Metabolism	Metabolism	10.31518	-0.980030074	0.307939	0.00146	0.006481	CHR vs HC
Glycosyltransferases	Glycan Biosynthesis and Metabolism	Metabolism	115816	0.156862596	0.039943	8.6E-05	0.00148	CHR vs HC
Peptidoglycan biosynthesis	Glycan Biosynthesis and Metabolism	Metabolism	237202.6	0.117040349	0.044335	0.008292	0.024542	CHR vs HC
alpha-Linolenic acid metabolism	Lipid Metabolism	Metabolism	9275.722	-0.516305412	0.147561	0.000467	0.003748	CHR vs HC
Arachidonic acid metabolism	Lipid Metabolism	Metabolism	19419.39	-0.250967832	0.086068	0.003546	0.012696	CHR vs HC
Biosynthesis of unsaturated fatty acids	Lipid Metabolism	Metabolism	72074.06	-0.301132429	0.119166	0.011504	0.0318	CHR vs HC
Ether lipid metabolism	Lipid Metabolism	Metabolism	2971.507	-0.702932032	0.178657	8.34E-05	0.00148	CHR vs HC
Fatty acid metabolism	Lipid Metabolism	Metabolism	161288.2	-0.47124427	0.129489	0.000273	0.002941	CHR vs HC
Sphingolipid metabolism	Lipid Metabolism	Metabolism	16384.07	0.483463338	0.158647	0.002308	0.009264	CHR vs HC
Synthesis and degradation of ketone bodies	Lipid Metabolism	Metabolism	34891.72	-0.634663333	0.161644	8.63E-05	0.00148	CHR vs HC

One carbon pool by folate	Metabolism of Cofactors and Vitamins	Metabolism	167500.1	0.130783177	0.035824	0.000262	0.002941	CHR vs HC
Pantothenate and CoA biosynthesis	Metabolism of Cofactors and Vitamins	Metabolism	171798.9	0.112452927	0.034102	0.000975	0.005196	CHR vs HC
Porphyrin and chlorophyll metabolism	Metabolism of Cofactors and Vitamins	Metabolism	216449.1	0.125240824	0.046672	0.007287	0.02201	CHR vs HC
Retinol metabolism	Metabolism of Cofactors and Vitamins	Metabolism	27654.28	-0.251965068	0.088419	0.004376	0.014922	CHR vs HC
Thiamine metabolism	Metabolism of Cofactors and Vitamins	Metabolism	117691.8	0.109806027	0.037665	0.003553	0.012696	CHR vs HC
beta-Alanine metabolism	Metabolism of Other Amino Acids	Metabolism	100074.2	-0.354928089	0.120133	0.003132	0.011617	CHR vs HC
D-Alanine metabolism	Metabolism of Other Amino Acids	Metabolism	31208.1	0.151183638	0.059936	0.011656	0.031917	CHR vs HC
D-Glutamine and D-glutamate metabolism	Metabolism of Other Amino Acids	Metabolism	43543.47	0.120643983	0.035087	0.000585	0.003748	CHR vs HC
Glutathione metabolism	Metabolism of Other Amino Acids	Metabolism	125914	-0.214535658	0.055178	0.000101	0.00148	CHR vs HC
Selenocompound metabolism	Metabolism of Other Amino Acids	Metabolism	100587.5	0.09387508	0.036594	0.010308	0.029322	CHR vs HC
Biosynthesis of ansamycins	Metabolism of Terpenoids and Polyketides	Metabolism	16913.57	0.234337243	0.059321	7.81E-05	0.00148	CHR vs HC
Biosynthesis of vancomycin group antibiotics	Metabolism of Terpenoids and Polyketides	Metabolism	13271.55	0.261948163	0.105197	0.012771	0.03433	CHR vs HC
Geraniol degradation	Metabolism of Terpenoids and Polyketides	Metabolism	80047.87	-0.682027959	0.200768	0.000681	0.003837	CHR vs HC

Limonene and pinene degradation	Metabolism of Terpenoids and Polyketides	Metabolism	78073.11	-0.503952901	0.195714	0.010025	0.028799	CHR vs HC
Prenyltransferases	Metabolism of Terpenoids and Polyketides	Metabolism	91214.06	0.172777147	0.04553	0.000148	0.002062	CHR vs HC
Terpenoid backbone biosynthesis	Metabolism of Terpenoids and Polyketides	Metabolism	158599.4	0.119563036	0.030683	9.75E-05	0.00148	CHR vs HC
Zeatin biosynthesis	Metabolism of Terpenoids and Polyketides	Metabolism	13120.04	0.222948716	0.062597	0.000368	0.003374	CHR vs HC
Purine metabolism	Nucleotide Metabolism	Metabolism	661533.5	0.114783105	0.030809	0.000195	0.002482	CHR vs HC
Pyrimidine metabolism	Nucleotide Metabolism	Metabolism	485361.5	0.168497719	0.04639	0.000281	0.002941	CHR vs HC
Caprolactam degradation	Xenobiotics Biodegradation and Metabolism	Metabolism	50758.47	-0.666752535	0.203671	0.001062	0.005457	CHR vs HC
Chloroalkane and chloroalkene degradation	Xenobiotics Biodegradation and Metabolism	Metabolism	63580.22	-0.259999646	0.099877	0.009236	0.026792	CHR vs HC
Drug metabolism - cytochrome P450	Xenobiotics Biodegradation and Metabolism	Metabolism	52865.43	-0.4349296	0.125031	0.000504	0.003748	CHR vs HC
Drug metabolism - other enzymes	Xenobiotics Biodegradation and Metabolism	Metabolism	71143.16	0.206685519	0.057533	0.000328	0.003096	CHR vs HC
Metabolism of xenobiotics by cytochrome P450	Xenobiotics Biodegradation and Metabolism	Metabolism	50681.19	-0.441342882	0.125187	0.000423	0.003643	CHR vs HC
Styrene degradation	Xenobiotics Biodegradation and Metabolism	Metabolism	20732.88	-0.331173829	0.132985	0.012763	0.03433	CHR vs HC
Cardiac muscle contraction	Circulatory System	Organismal Systems	14892.89	-0.407028522	0.148284	0.006052	0.019068	CHR vs HC
Bile secretion	Digestive System	Organismal Systems	164.9374	-1.434069895	0.296473	1.32E-06	7.72E-05	CHR vs HC
GnRH signaling pathway	Endocrine System	Organismal Systems	628.2358	-1.022755914	0.370411	0.00576	0.018381	CHR vs HC
Insulin signaling pathway	Endocrine System	Organismal Systems	20921.34	0.212624492	0.046493	4.8E-06	0.000235	CHR vs HC

PPAR signaling pathway	Endocrine System	Organismal Systems	42826.6	-0.210196152	0.08838	0.017391	0.043993	CHR vs HC
Renin-angiotensin system	Endocrine System	Organismal Systems	787.4457	-1.559350805	0.305923	3.45E-07	3.37E-05	CHR vs HC
Fc gamma R-mediated phagocytosis	Immune System	Organismal Systems	628.2871	-1.022414599	0.370376	0.005772	0.018381	CHR vs HC
Germination	Cellular Processes and Signaling	Unclassified	1028.226	0.853748103	0.324151	0.008444	0.02474	CHR vs HC
Membrane and intracellular structural molecules	Cellular Processes and Signaling	Unclassified	228468.8	-0.130127351	0.045649	0.004364	0.014922	CHR vs HC
Signal transduction mechanisms	Cellular Processes and Signaling	Unclassified	125779.5	-0.089628421	0.028462	0.001638	0.007059	CHR vs HC
Sporulation	Cellular Processes and Signaling	Unclassified	14387.41	0.896956664	0.218908	4.18E-05	0.00102	CHR vs HC
Protein folding and associated processing	Genetic Information Processing	Unclassified	236661.5	0.040991615	0.016087	0.010832	0.030516	CHR vs HC
Translation proteins	Genetic Information Processing	Unclassified	286551.2	0.088213378	0.029489	0.002777	0.010849	CHR vs HC
Carbohydrate metabolism	Metabolism	Unclassified	25214.39	0.229210345	0.098025	0.019372	0.048103	CHR vs HC
Energy metabolism	Metabolism	Unclassified	243993.4	-0.084330552	0.031409	0.007255	0.02201	CHR vs HC
Metabolism of cofactors and vitamins	Metabolism	Unclassified	53931.33	-0.092470766	0.03907	0.017942	0.044933	CHR vs HC
Nucleotide metabolism	Metabolism	Unclassified	15364.61	0.387591923	0.131468	0.003196	0.011707	CHR vs HC
MAPK signaling pathway - yeast	Signal Transduction	Environmental Information Processing	12154.45	0.166128227	0.049675	0.000825	0.012536	FES vs CHR
Phosphatidylinositol signaling system	Signal Transduction	Environmental Information Processing	35844.93	0.150835009	0.051502	0.003404	0.041269	FES vs CHR
VEGF signaling pathway	Signal Transduction	Environmental Information Processing	2.390499	-4.996937173	1.746542	0.004222	0.043883	FES vs CHR

Bacterial invasion of epithelial cells	Infectious Diseases	Human Diseases	488.4277	0.710388172	0.223304	0.001466	0.02032	FES vs CHR
Epithelial cell signaling in Helicobacter pylori infection	Infectious Diseases	Human Diseases	18388.83	-0.156738868	0.056512	0.005545	0.048894	FES vs CHR
Leishmaniasis	Infectious Diseases	Human Diseases	2.390499	-4.996937173	1.746542	0.004222	0.043883	FES vs CHR
Pertussis	Infectious Diseases	Human Diseases	9697.254	-0.870444108	0.233923	0.000198	0.004123	FES vs CHR
Staphylococcus aureus infection	Infectious Diseases	Human Diseases	12651.87	0.578987005	0.204834	0.004704	0.047206	FES vs CHR
Glycine, serine and threonine metabolism	Amino Acid Metabolism	Metabolism	244869.2	-0.044178191	0.015926	0.005537	0.048894	FES vs CHR
Histidine metabolism	Amino Acid Metabolism	Metabolism	145908.4	-0.154132305	0.039551	9.74E-05	0.00218	FES vs CHR
Betalain biosynthesis	Biosynthesis of Other Secondary Metabolites	Metabolism	142.868	-2.324283576	0.444992	1.76E-07	1.71E-05	FES vs CHR
Isoquinoline alkaloid biosynthesis	Biosynthesis of Other Secondary Metabolites	Metabolism	16160.42	-0.284664725	0.066504	1.87E-05	0.000971	FES vs CHR
Novobiocin biosynthesis	Biosynthesis of Other Secondary Metabolites	Metabolism	36348.02	-0.107724219	0.030388	0.000393	0.007143	FES vs CHR
Tropane, piperidine and pyridine alkaloid biosynthesis	Biosynthesis of Other Secondary Metabolites	Metabolism	32401.18	-0.258287337	0.063773	5.12E-05	0.00149	FES vs CHR
Glyoxylate and dicarboxylate metabolism	Carbohydrate Metabolism	Metabolism	151843.5	-0.135746393	0.049538	0.006139	0.049499	FES vs CHR
Pyruvate metabolism	Carbohydrate Metabolism	Metabolism	307447.1	0.074287517	0.022294	0.000862	0.012536	FES vs CHR
Nitrogen metabolism	Energy Metabolism	Metabolism	193001.1	-0.102288503	0.028851	0.000392	0.007143	FES vs CHR
Cytochrome P450	Enzyme Families	Metabolism	30.58497	2.460731332	0.414448	2.9E-09	4.21E-07	FES vs CHR
Glycosphingolipid biosynthesis - lacto and neolacto series	Glycan Biosynthesis and Metabolism	Metabolism	8.603878	1.084054017	0.277071	9.13E-05	0.00218	FES vs CHR

Lipopolysaccharide biosynthesis proteins	Glycan Biosynthesis and Metabolism	Metabolism	156384.4	-0.178797857	0.059516	0.002663	0.034638	FES vs CHR
Fatty acid biosynthesis	Lipid Metabolism	Metabolism	153828.2	0.064939529	0.023353	0.005423	0.048894	FES vs CHR
Primary bile acid biosynthesis	Lipid Metabolism	Metabolism	2414.473	-1.083938663	0.317477	0.00064	0.010949	FES vs CHR
Steroid hormone biosynthesis	Lipid Metabolism	Metabolism	2053.516	-0.737645923	0.256042	0.003965	0.043883	FES vs CHR
Lipoic acid metabolism	Metabolism of Cofactors and Vitamins	Metabolism	20031.3	0.238135445	0.057181	3.12E-05	0.001135	FES vs CHR
Porphyrin and chlorophyll metabolism	Metabolism of Cofactors and Vitamins	Metabolism	201248.9	-0.254680139	0.054241	2.66E-06	0.000194	FES vs CHR
Riboflavin metabolism	Metabolism of Cofactors and Vitamins	Metabolism	74194.94	-0.118989172	0.028028	2.18E-05	0.000971	FES vs CHR
Ubiquinone and other terpenoid-quinone biosynthesis	Metabolism of Cofactors and Vitamins	Metabolism	82744.69	-0.124344915	0.044436	0.005137	0.048894	FES vs CHR
beta-Alanine metabolism	Metabolism of Other Amino Acids	Metabolism	78416.53	-0.318206926	0.116847	0.006464	0.049499	FES vs CHR
Biosynthesis of type II polyketide products	Metabolism of Terpenoids and Polyketides	Metabolism	185.5956	-2.1127369	0.499447	2.34E-05	0.000971	FES vs CHR
Tetracycline biosynthesis	Metabolism of Terpenoids and Polyketides	Metabolism	44358.99	0.21227003	0.052164	4.72E-05	0.00149	FES vs CHR
Atrazine degradation	Xenobiotics Biodegradation and Metabolism	Metabolism	11144.49	-0.548533693	0.199249	0.005905	0.049499	FES vs CHR
Fluorobenzoate degradation	Xenobiotics Biodegradation and Metabolism	Metabolism	8666.857	-1.146700593	0.285858	6.04E-05	0.001597	FES vs CHR
Nitrotoluene degradation	Xenobiotics Biodegradation and Metabolism	Metabolism	14230.71	-0.262758721	0.095649	0.006012	0.049499	FES vs CHR
Melanogenesis	Endocrine System	Organismal Systems	108.3263	-3.325476918	0.499124	2.69E-11	7.83E-09	FES vs CHR

Cell motility and secretion	Cellular Processes and Signaling	Unclassified	65616.84	-0.226066922	0.066871	0.000723	0.011691	FES vs CHR
Other ion-coupled transporters	Cellular Processes and Signaling	Unclassified	358555.8	-0.118413449	0.039527	0.002738	0.034638	FES vs CHR
Protein folding and associated processing	Genetic Information Processing	Unclassified	222606.5	-0.053113828	0.019503	0.006463	0.049499	FES vs CHR
Transcription related proteins	Genetic Information Processing	Unclassified	1678.188	-0.642385149	0.220674	0.003603	0.041934	FES vs CHR

Supplementary Figures

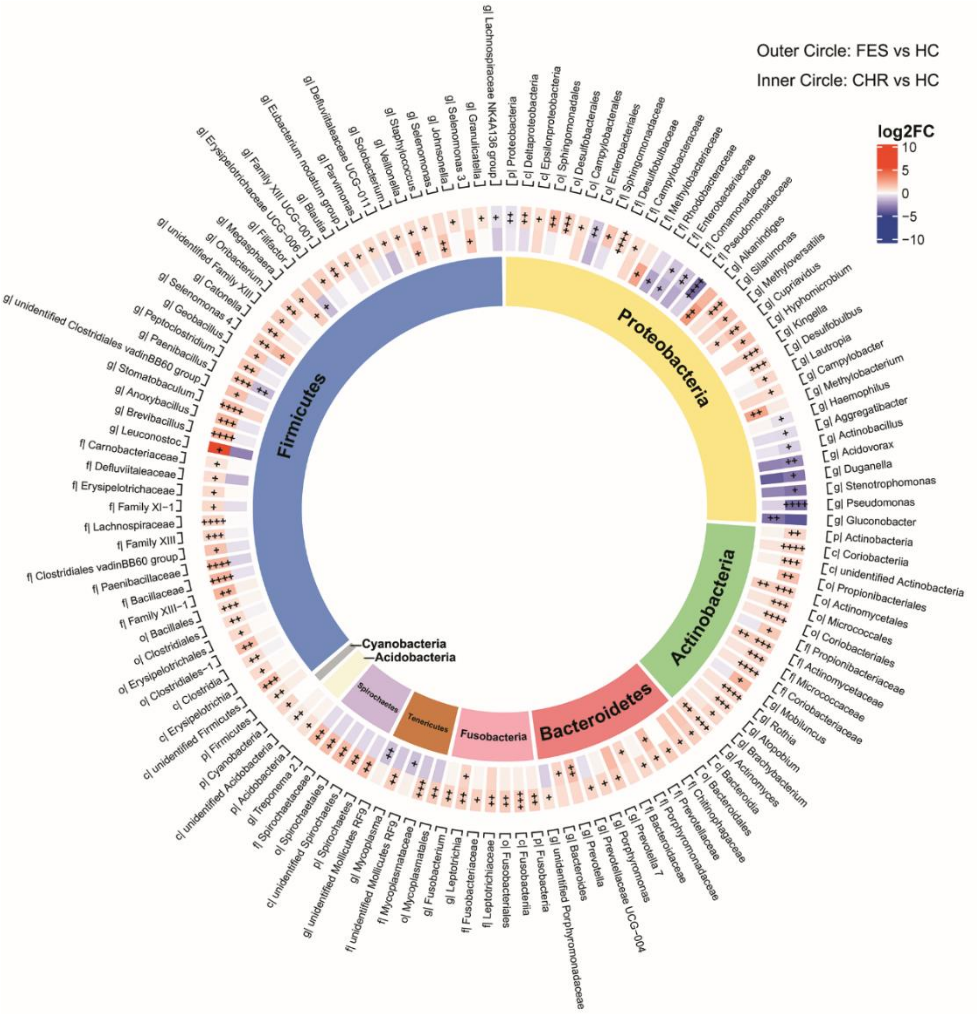

Supplementary Figure 1. Drastic alterations in the salivary microbiota were observed in the FES group rather than the CHR group. There were 114 differentially abundant taxa in the FES group relative to HCs and 24 altered taxa in the CHR group compare to HCs, among which ten taxa showing the same trend overlapped between the FES and CHR groups compared to HCs. FES, first-episode schizophrenia; CHR, clinical high risk for psychosis; HCs, healthy controls; p, phylum; c, class; o, order; f, family; g, genus. False discovery rate adjusted q-values were calculated based on p-values estimated by quantile regression, adjusting for age, sex and the education level. $^{+}$ $q < 0.05$; $^{++}$ $q < 0.01$; $^{+++}$ $q < 0.001$; $^{++++}$ $q < 0.0001$.

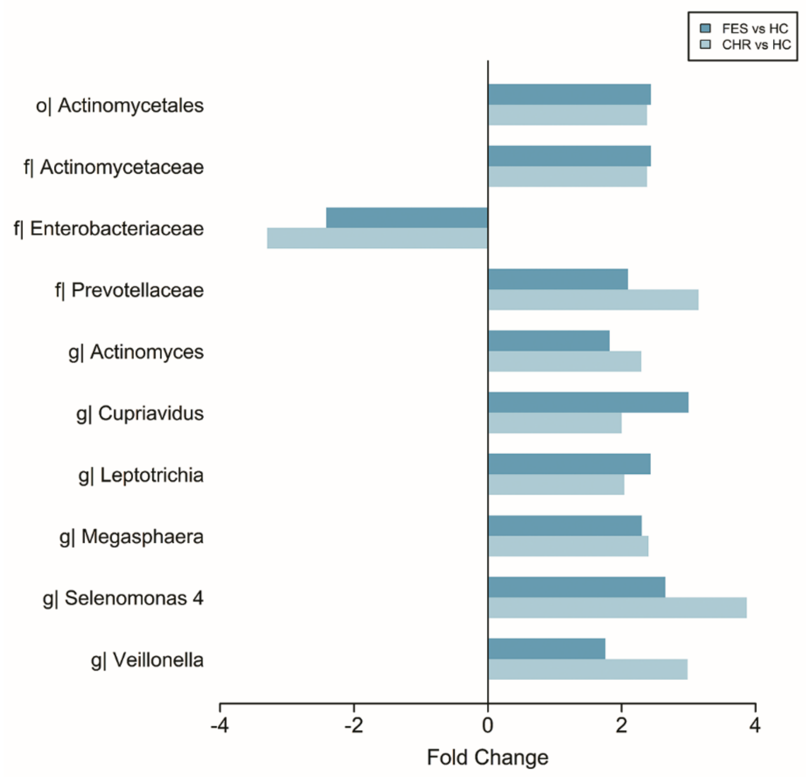

Supplementary Figure 2. Overlapping differentially abundant taxa in the FES and CHR groups relative to HCs. The order Actinomycetales, the families Actinomycetaceae and Prevotellaceae, and the genera *Actinomyces*, *Cupriavidus*, *Leptotrichia*, *Megasphaera*, *Selenomonas 4* and *Veillonella* were all enriched in the FES and CHR groups compared to HCs, while the family Enterobacteriaceae depleted. FES, first-episode schizophrenia; CHR, clinical high risk for psychosis; HCs, healthy controls; o, order; f, family; g, genus.

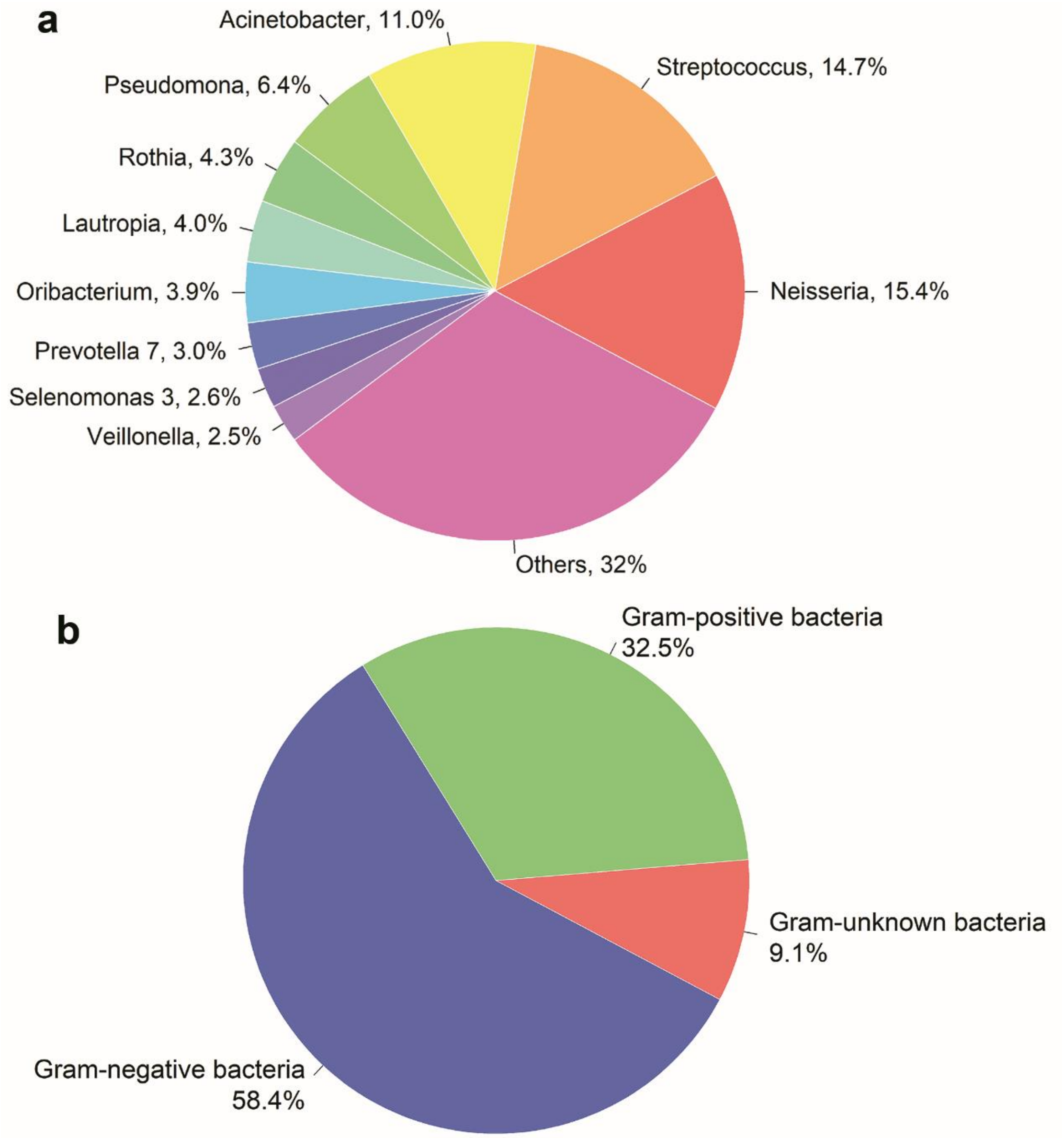

Supplementary Figure 3. Dominant genera in saliva. (a) The pie plot shows the 10 most abundant salivary genera in 208 subjects, where *Neisseria*, *Streptococcus* and *Acinetobacter* constituted more than 40% of the bacterial communities in saliva. (b) Relative abundances of gram-positive and gram-negative bacteria in saliva. The pie plot shows that gram-negative bacteria were more abundant than gram-negative bacteria in saliva.

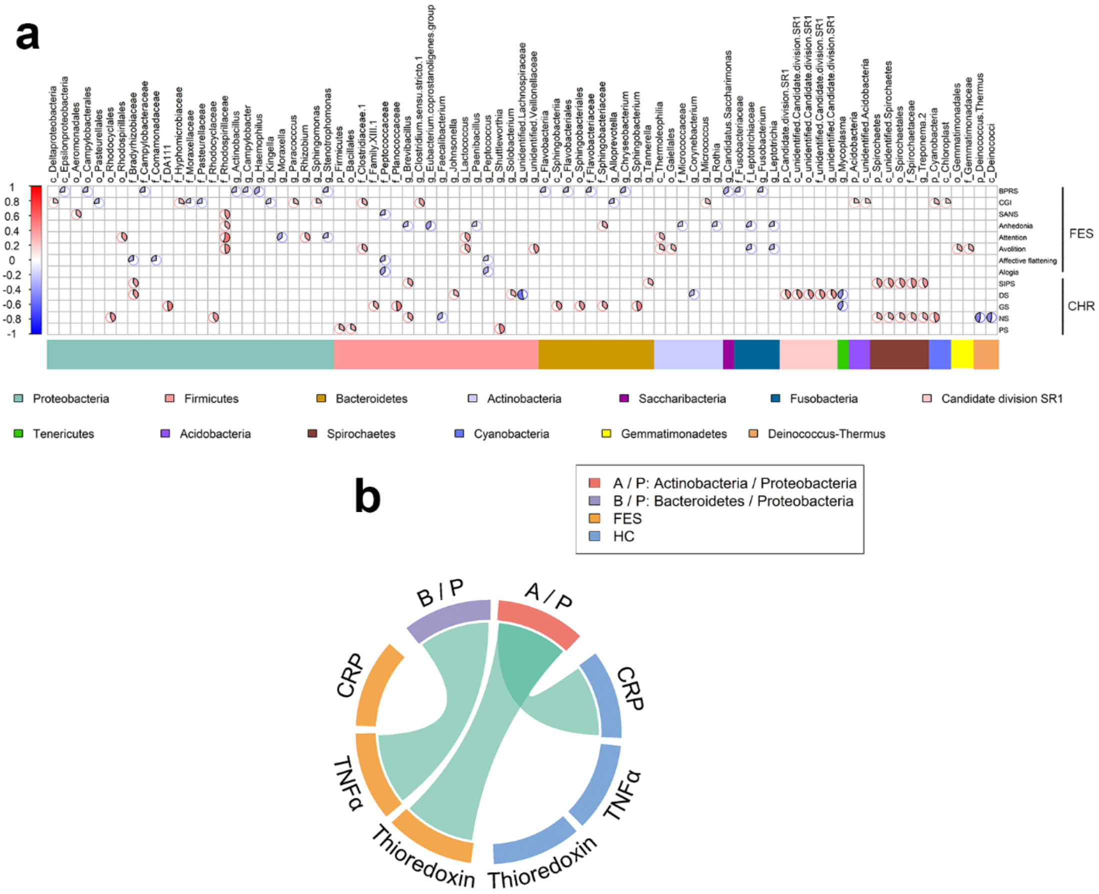

Supplementary Figure 4. The correlations between salivary taxa and the clinical characteristics of FES and CHR patients. (a) The heatmap shows the relationships between salivary microbiota and symptoms of two disease statuses. Only statistically significant correlations ($p < 0.05$) are shown. Red pies denote positive correlations, while blue pies denote negative correlations. BPRS, brief psychiatric rating scale; CGI-S, clinical global impressions severity scale; SANS, scale for the assessment of negative symptoms; SIPS, structured interview of prodrome syndromes; DS, disorganized symptom; GS, general symptom; NS, negative symptoms; PS, positive symptoms; p, phylum; c, class; o, order; f, family; g, genus. (b) The Bacteroidetes/Proteobacteria ratio was negatively correlated with TNFα in the FES group. The Actinobacteria/Proteobacteria ratio was inversely related to thioredoxin in the FES

group and negatively associated with CRP in HCs, respectively. Green curves represent negative correlations. A/P, Actinobacteria/Proteobacteria; B/P, Bacteroidetes/Proteobacteria; FES, first-episode schizophrenia; HCs, healthy controls; CRP, C-reactive protein.

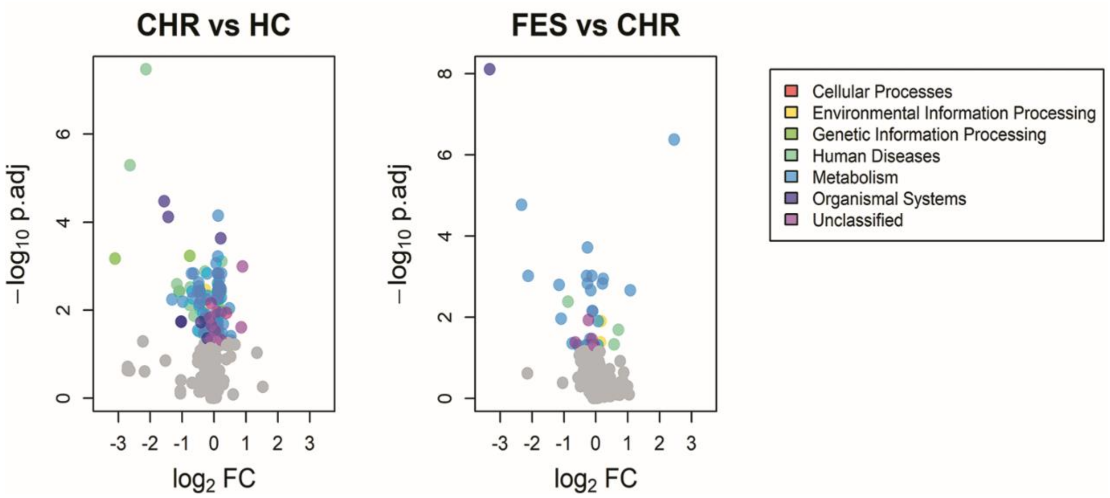

Supplementary Figure 5. A set of volcano plots shows the differentially abundant KEGG pathways in CHR vs HC (left) and FES vs CHR (right). KEGG, Kyoto Encyclopedia of Gene and Genomes; FES, first-episode schizophrenia; CHR, clinical high risk for psychosis; HCs, healthy controls; FC, Fold Change.

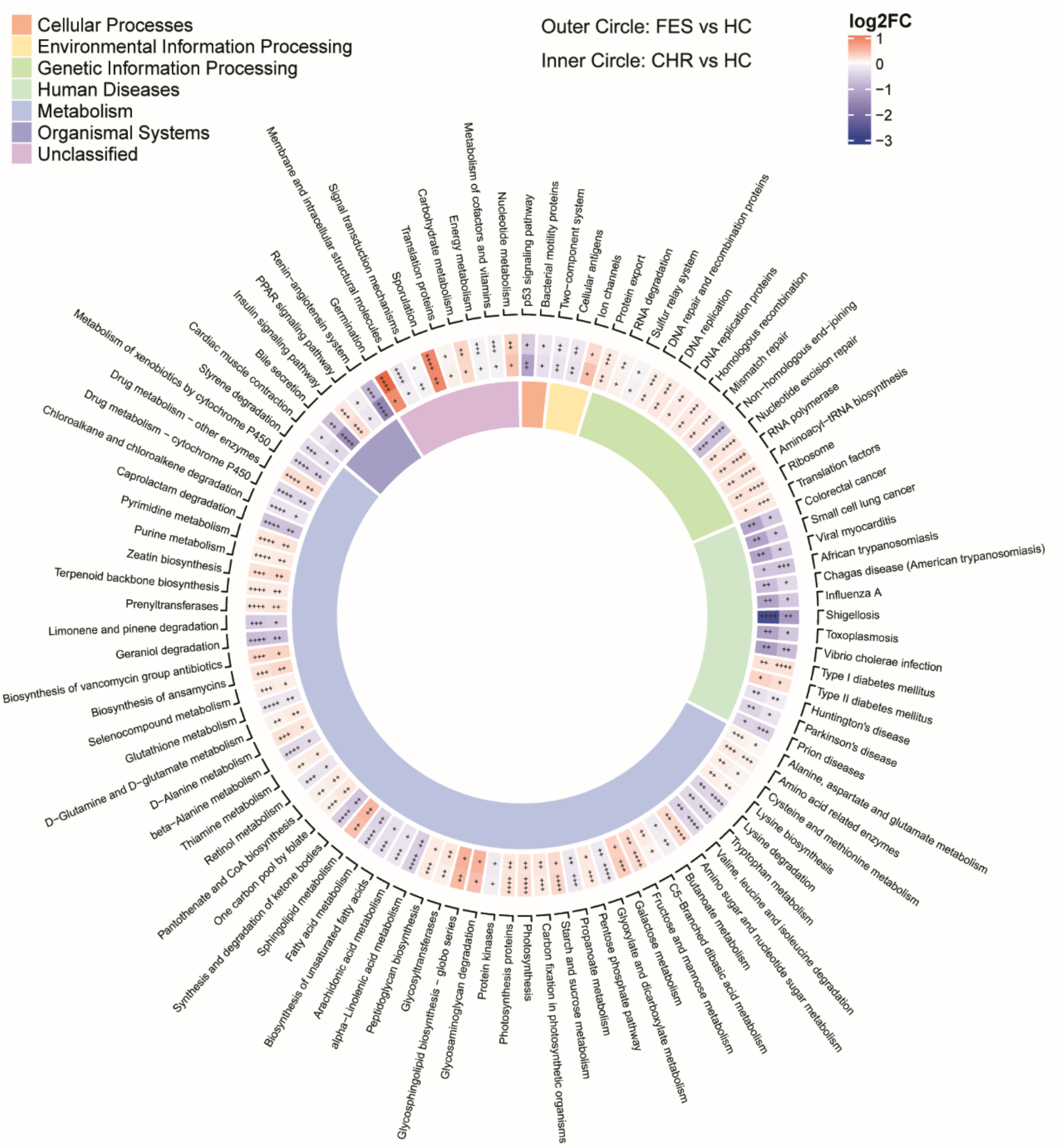

Supplementary Figure 6. The Circos heatmap shows 101 overlapping differentially abundant KEGG pathways that exhibited parallel alterations in both the FES and CHR groups relative to HCs, over 50% of which were metabolism-related pathways. KEGG, Kyoto Encyclopedia of Gene and Genomes; FES, first-episode schizophrenia; CHR, clinical high risk for psychosis; HCs, healthy controls, FC, Fold Change. False discovery rate adjusted *q*-values were calculated based on *p*-values estimated by DESeq2, adjusting for age, sex and the education level. $^{+}$ $q < 0.05$; $^{++}$ $q < 0.01$; $^{+++}$ $q < 0.001$; $^{++++}$ $q < 0.0001$.
