# Supplementary material for: Salivary microbiome profiling reveals a dysbiotic schizophrenia-associated microbiota
Source: NPJ Schizophr. 2021 Oct 28;7:51. doi: 10.1038/s41537-021-00180-1 (PMC8553823; doi:10.1038/s41537-021-00180-1)
Supplement: Supplementary file 2 — Reporting Summary [file 41537_2021_180_MOESM2_ESM.pdf]

nature portfolio

Corresponding author(s): Chunling Wan

Last updated by author(s): Aug 22, 2021

Reporting Summary

Nature Portfolio wishes to improve the reproducibility of the work that we publish. This form provides structure for consistency and transparency in reporting. For further information on Nature Portfolio policies, see our Editorial Policies and the Editorial Policy Checklist.

Statistics

For all statistical analyses, confirm that the following items are present in the figure legend, table legend, main text, or Methods section.

n/a	Confirmed	
☐	☒	The exact sample size (*n*) for each experimental group/condition, given as a discrete number and unit of measurement
☐	☒	A statement on whether measurements were taken from distinct samples or whether the same sample was measured repeatedly
☐	☒	The statistical test(s) used AND whether they are one- or two-sided<br>*Only common tests should be described solely by name; describe more complex techniques in the Methods section.*
☐	☒	A description of all covariates tested
☐	☒	A description of any assumptions or corrections, such as tests of normality and adjustment for multiple comparisons
☐	☒	A full description of the statistical parameters including central tendency (e.g. means) or other basic estimates (e.g. regression coefficient) AND variation (e.g. standard deviation) or associated estimates of uncertainty (e.g. confidence intervals)
☐	☒	For null hypothesis testing, the test statistic (e.g. *F*, *t*, *r*) with confidence intervals, effect sizes, degrees of freedom and *P* value noted<br>*Give P values as exact values whenever suitable.*
☒	☐	For Bayesian analysis, information on the choice of priors and Markov chain Monte Carlo settings
☒	☐	For hierarchical and complex designs, identification of the appropriate level for tests and full reporting of outcomes
☐	☒	Estimates of effect sizes (e.g. Cohen's *d*, Pearson's *r*), indicating how they were calculated

Our web collection on statistics for biologists contains articles on many of the points above.

Software and code

Policy information about availability of computer code

Data collection	The signals from the Customized Human Cytokine Antibody Array (RayBiotech, Norcross, GA, USA) were detected using a GenePix 4 000B (Axon Instruments, Foster City, CA, USA).
Data analysis	These following softwares were used: GenePix Pro 6.0, FLASH v1.2.7, QIIME v1.7.0, UCHIME v4.2, Uparse v7.0.1001, RDP classifier v2.7, Cytoscape v3.7.2, SPSS v24 and R v3.4.4.

For manuscripts utilizing custom algorithms or software that are central to the research but not yet described in published literature, software must be made available to editors and reviewers. We strongly encourage code deposition in a community repository (e.g. GitHub). See the Nature Portfolio guidelines for submitting code & software for further information.

Data

Policy information about availability of data

All manuscripts must include a data availability statement. This statement should provide the following information, where applicable:

- Accession codes, unique identifiers, or web links for publicly available datasets
- A description of any restrictions on data availability
- For clinical datasets or third party data, please ensure that the statement adheres to our policy

All sequencing data associated with this study are available in the NCBI SRA database (accession number PRJNA647054).

Field-specific reporting

Please select the one below that is the best fit for your research. If you are not sure, read the appropriate sections before making your selection.

☒ Life sciences ☐ Behavioural & social sciences ☐ Ecological, evolutionary & environmental sciences

For a reference copy of the document with all sections, see nature.com/documents/nr-reporting-summary-flat.pdf

Life sciences study design

All studies must disclose on these points even when the disclosure is negative.

Sample size	No sample size calculation was performed. All available samples from participants who met inclusion criteria were included.
Data exclusions	Data that were more than 1.5 IQR below Q1 or more than 1.5 IQR above Q3 in alpha indices and the ratios of taxa were excluded.
Replication	No replication was performed.
Randomization	Case control design, no randomization.
Blinding	16S rRNA gene sequencing in saliva samples and assessment of levels of C-reactive protein, and IFNγ, IL-1β, IL-8, TNFα, S100B and L-aspartate in plasma samples were blind to participant diagnosis.

Reporting for specific materials, systems and methods

We require information from authors about some types of materials, experimental systems and methods used in many studies. Here, indicate whether each material, system or method listed is relevant to your study. If you are not sure if a list item applies to your research, read the appropriate section before selecting a response.

Materials & experimental systems

n/a	Involved in the study	
☐	☒	Antibodies
☒	☐	Eukaryotic cell lines
☒	☐	Palaeontology and archaeology
☒	☐	Animals and other organisms
☐	☒	Human research participants
☐	☒	Clinical data
☒	☐	Dual use research of concern

Methods

n/a	Involved in the study	
☒	☐	ChIP-seq
☒	☐	Flow cytometry
☒	☐	MRI-based neuroimaging

Antibodies

Antibodies used	This statement was described in the Methods section.
Validation	The source of antibodies employed in the study were reported in the Methods section.

Human research participants

Policy information about studies involving human research participants

Population characteristics	Participants included individuals with schizophrenia or schizophreniform disorder confirmed by the Structured Clinical Interview for DSM-IVR, individuals at clinical high-risk for psychosis comfirmed by the Structured Interview of Prodrome Syndromes (SIPS), or individuals with no current psychiatric diagnosis as determined by the Structured Clinical Interview for DSM-IVR. Age range was 15-46. Both males and females included.
Recruitment	Patients were recruited from outpatient clinics specializing in psychotic disorders. Comparison participants were recruited through advertisements.
Ethics oversight	Research Ethics Committee at the Shanghai Mental Health Centre

Note that full information on the approval of the study protocol must also be provided in the manuscript.

Clinical data

Policy information about clinical studies

All manuscripts should comply with the ICMJE guidelines for publication of clinical research and a completed CONSORT checklist must be included with all submissions.

Clinical trial registration	This was not a clinical trial. It was a case control investigation.
Study protocol	Available from the corresponding author upon reasonable request.
Data collection	Participants for the current study were recruited between January 2013 and July 2015.
Outcomes	The symptomatic severities of patients with schizophrenia were assessed by the Brief Psychiatric Rating Scale (BPRS), Clinical Global Impressions Severity Scale (CGI-S), and Scale for the Assessment of Negative Symptoms (SANS) tests. The symptomatic severities of patients at clinical high-risk for psychosis were assessed by Structured Interview of Prodrome Syndromes (SIPS).
